# Formation of diverse polycyclic spirooxindoles via three-component reaction of isoquinolinium salts, isatins and malononitrile

**DOI:** 10.1038/srep41024

**Published:** 2017-01-20

**Authors:** Jing Sun, Guo-liang Shen, Ying Huang, Chao-Guo Yan

**Affiliations:** 1 College of Chemistry & Chemical Engineering, Yangzhou University, Yangzhou 225002, China

## Abstract

The triethylamine promoted three-component reaction of *N*-(4-nitrobenzyl), *N*-ethoxycarbonylmethylisoquinolinium bromide, isatins and malononitrile in ethanol afforded spiro[indoline-3,2′-pyrrolo[2,1-*a*]isoquinolines] in good yields and with high diastereoselectivity. The similar reaction of *N*-cyanomethylisoquinolinium chloride mainly gave complex indolo[2″,3″:2′,3′]pyrrolo[3′,4′:4,5]pyrrolo[2,1-a]isoquinoline derivatives. However, the three-component reaction of *N*-cyanomethylisoquinolinium chloride, isatins and ethyl cyanoacetate mainly resulted in functionalized **s**piro[indoline-3,8′-pyrido[2′,3′:4,5]pyrrolo[2,1-a]isoquinolines].

The cyclic nitrogen *N*-ylides such as pyridinium, thiazolium, quinolinium, isoquinolinium methylides are a special group of reactive azomethine ylides, which can be easily generated from the deprotonation of imidazolium, thiazolium, pyridinium salts and their benzo-fused analogs with reactive *N*-methyl group connecting with stronger electron-withdrawing groups^1^,^2^,^3^,^4^,^5^,^6^,^7^,^8^. Because of cyclic nitrogen *N*-ylides have heterocyclic aromatic character, basicity, electron-attracting positive nitrogen atom, and the strongly electron-withdrawing substituent like carbonyl, cyano, or nitro groups connecting with methylene group, they have been become one of practical potential synthons in synthetic reactions^9^,^10^,^11^,^12^,^13^,^14^,^15^,^16^,^17^,^18^. The most common reaction is 1,3-dipolar cycloaddition of cyclic nitrogen *N*-ylides such as pyridinium ylide with various electron-deficient acetylene and alkenes to give indolizine derivatives, in which the pyridyl ring is retained^19^,^20^,^21^,^22^,^23^,^24^,^25^,^26^. The second widely used reaction is the reaction of cyclic nitrogen *N*-ylides with alkenes bearing electron-withdrawing groups to give the corresponding cyclopropanes, 2,3-dihydrofurans and other heterocyclic compounds, in which the pyridyl unit iseliminated^27^,^28^,^29^,^30^,^31^,^32^,^33^,^34^. According to the structures of the substrates and the reaction conditions, the reaction of the cyclic nitrogen *N*-ylides showed very interesting molecular diversity^26^,^35^,^36^,^37^,^38^,^39^. In the past few years, we investigated the multicomponent reactions by employing easily generated cyclic nitrogen ylides as the main substrates and have successfully developed a number of highly efficient protocols for synthesis of some biologically important nitrogen-containing heterocyclic compounds^40^,^41^,^42^,^43^,^44^,^45^,^46^,^47^,^48^. Recently, we successfully found that the cycloaddition reaction of the cyclic nitrogen *N*-ylides with reactive 3-phenacylideneoxindoles resulted in diverse spirooxindole systems including spiro[indoline-3,1′-pyrrolo[2,1-a]isoquinolines], spiro[cyclopropane-1,3′-indolines], 3-furan-3(2 H)-ylidene)indolin-2-ones, spiro[benzo[d]pyrrolo[2,1-b]thiazole-3,3′-indolines], and complex cyclopentyl dispiroxindoles^49^,^50^,^51^,^52^,^53^,^54^,^55^,^56^,^57^. We also found that three-component reactions of *N*-benzylbenzimidazolium salts, isatins and malononitrile or ethyl cyanoacetate gave a series of the novel zwitterionic salts and the unexpected products with opening of the imidazole ring^58^. These results together with the previously reports^59^,^60^,^61^,^62^,^63^,^64^ indicated that the 1,3-dipolar cycloaddition reactions of cyclic nitrogen *N*-ylides with 3-methyleneoxindoles have fruitful chemistry. Due to the spirooxindole existing in a large number of naturally occurring and medicinally relevant substances, the development of efficient method for constructing the spirooxindole motif is of great importance in synthetic organic and medicinal chemistry^65^,^66^,^67^,^68^,^69^,^70^,^71^,^72^. Against this background and in continuation of our efforts to develop new efficient synthetic methods for complex spirooxindoles^73^,^74^,^75^,^76^,^77^,^78^,^79^,^80^,^81^,^82^,^83^, herein we wish to report the interesting results of three-component reactions of *N*-(4-nitrobenzyl), *N*-ethoxycarbonylmethyl and *N*-cyanomethylisoquinolinium salts with isatins and malononitrile as well as ethyl cyanoacetate.

## Results and Discussion

**Figure 1:**
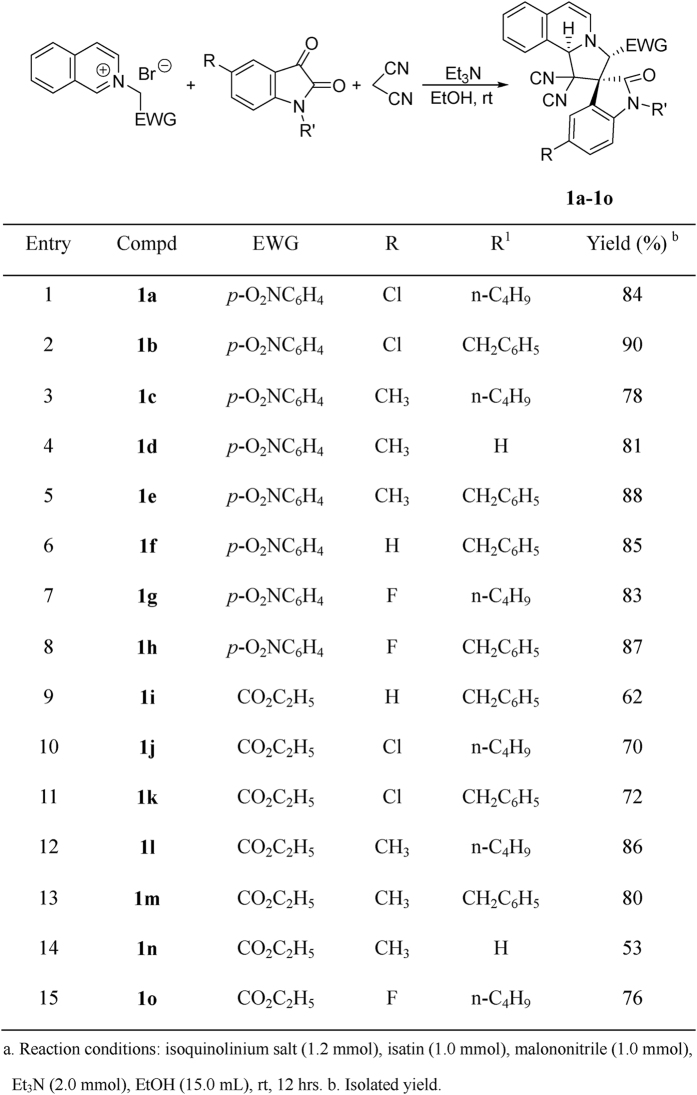
Synthesis of spiro[indoline-3,2′-pyrrolo[2,1-*a*]isoquinolines] 1a-1o^a^.

**Figure 2:**
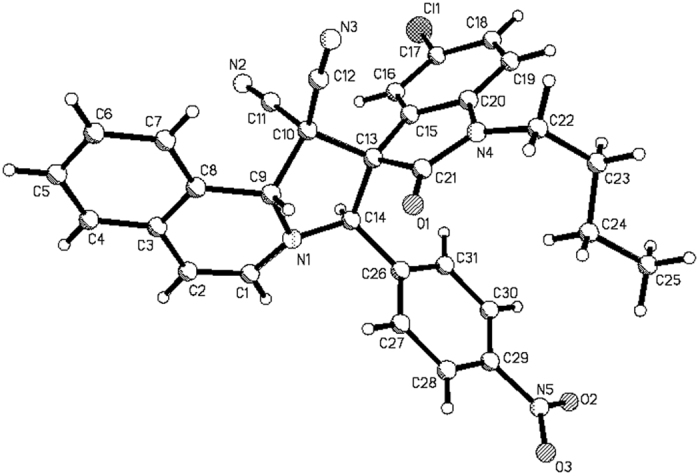
Molecular structure of spiro compound 1a.

According to our previously established reaction conditions^58^, an equivalent amount of *N*-(4-nitrobenzyl)isoquinolinium bromide, isatins and malononitrile in ethanol in the presence of triethylamine as base was stirred at room temperature overnight. The reaction was accomplished to give the expected spiro[indoline-3,2′-pyrrolo[2,1-*a*]isoquinolines] **1a-1h** in satisfactory yields (Fig. 1, entries 1–8). The pure products can be easily obtained after simple filtration of the resulting precipitates and washing with cold ethanol. ^1^H NMR and ^13^C NMR spectroscopy clearly indicated that only one isomer exists in the obtained products **1a-1h**. The single crystal structures of the three compounds **1a** (Fig. 2), **1c** (Fig. s1 in SI) and **1e** (Fig. s2 in SI) were successfully determined by X-ray diffraction. The three single crystal structures all showed that the *p*-nitrophenyl group and phenyl group of oxindoline moiety exist in *trans*-configuration. On the basis of spectroscopy and single crystal structures, it can be concluded that the thermodynamically stable *trans-*diastereoisomer of spiro[indoline-3,2′-pyrrolo[2,1*-a*]isoquinolines] was predominately produced in this base promoted three-component reaction. Moreover, when *N*-ethoxycarbonylmethylisoquinolinium bromide was employed under the same reaction conditions, the desired spiro products **1i-1o** were also prepared in good yields (Fig. 1, entries 9–15). The crystal structure of the compound **1j** (Fig. s3 in SI) indicated that it has the same configuration to that of compounds **1a, 1c** and **1e**. Thus, the relative *trans*-configuration of spiro products **1i-1o** were also elucidated on the basis of ^1^H NMR spectra and determination of single crystal structure of the compound **1j**. On the other hand, when *N*-phenacylisoquinolinum bromides were employed in the three-component reaction, we were very disappointed to find that the reaction resulted in complex mixtures, which were unable to be separated out. It has been known that *N*-phenacylisoquinolinum bromides usually have higher reactivity than that of *N-p*-nitrobenzyl- and *N*-ethoxycarbonylmethylisoquinolinium bromides^49^,^50^,^51^,^52^,^53^,^54^,^55^,^56^,^57^. In order to get good results, we carefully examined the reaction conditions for *N*-phenacylisoquinolinum bromides and did not get the expected products. This results might be due to the instability of the expected spiro[indoline-3,2′-pyrrolo[2,1-*a*]isoquinolines] with benzoyl groups.

**Figure 3:**
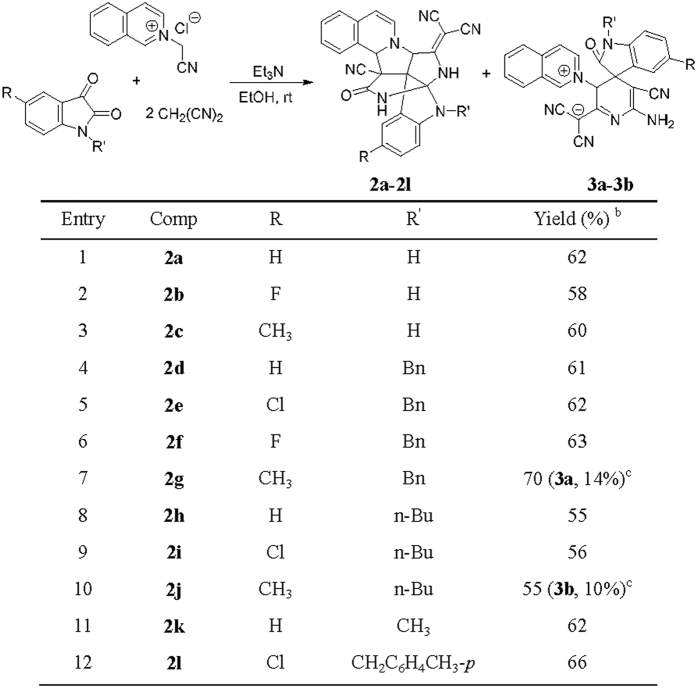
Synthesis of indolo[2″,3″:2′,3′]pyrrolo[3′,4′:4,5]pyrrolo[2,1-a]isoquinolines 2a-2l^a^.

**Figure 4:**
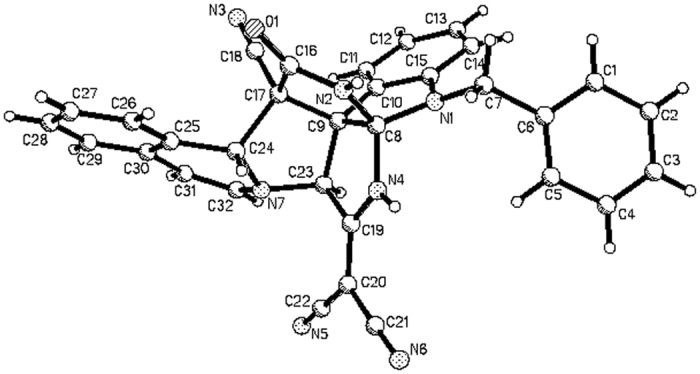
Molecular structure of spiro compound 2d.

**Figure 5:**
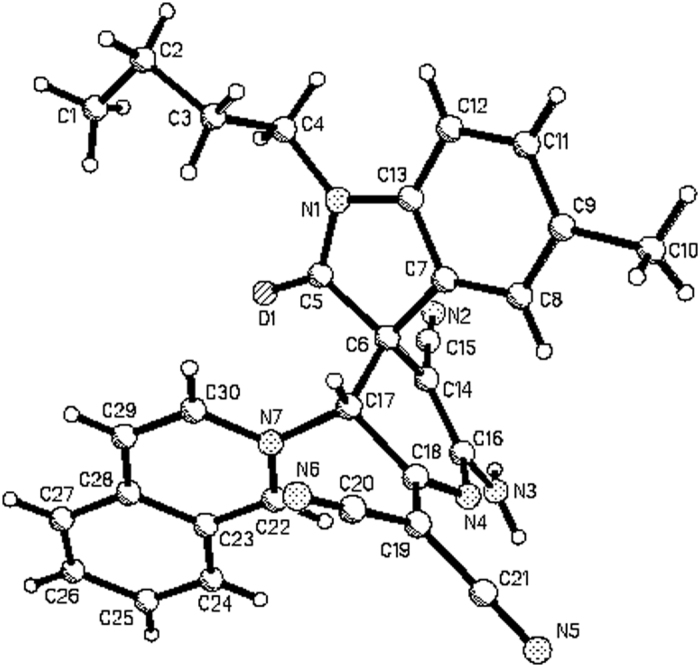
Molecular structure of spiro compound 3b.

In order to establish the generality of this three-component reaction, we extended the above reaction protocol to *N*-cyanomethylisoquinolinium chloride, which was previously prepared from reaction of isoquinoline and chloroacetonitrile in refluxing acetonitrile. Under similar reaction conditions, the three-component reaction of isatins, malononitrile with *N*-cyanomethylisoquinolinium chloride afforded complex polycyclic compounds **2a-2l** as the main products in moderate to good yields and the corresponding zwitterionic compounds as byproducts in very low yields (Fig. 3). For convenience, only two zwitterionic compounds **3a** and **3b** were successfully separated out in 14% and 10% yields, respectively. The structures of the obtained compounds **2a-2l** and **3a-3b** were fully characterized by IR, HRMS, ^1^H and ^13^C NMR spectroscopy. The single crystal structures of compounds **2d** (Fig. 4), **2e** (Fig. s4 in SI), **2k** (Fig. s5 in SI) and **3b** (Fig. 5) were determined by X-ray diffraction. Comparing the structures of compounds **1a-1o** with that of compounds **2a-2l**, it can be seen that compounds **2a-2l** have two additional pyrrolidine rings on the skeleton of spiro[indoline-3,2′-pyrrolo[2,1-*a*]isoquinolines] **1a-1o**, which suggested that the initially formed spiro compounds **1** underwent further transformations in the reaction system. The byproducts **3a-3b** were obviously coming from a separate reaction mechanism.

**Figure 6:**
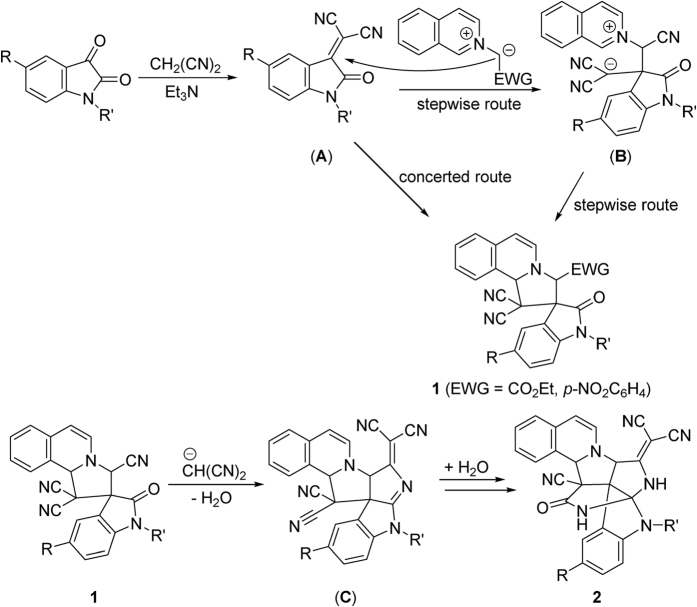
Proposed formation mechanism for spiro compounds 1 and 2.

In order to explain the formation mechanism of the spiro compounds **1** and **2**, a plausible reaction mechanism was proposed on the basis of the known 1,3-dipolar cycloaddition reactions of the cyclic nitrogen ylides^49^,^50^,^51^,^52^,^53^,^54^,^55^,^56^,^57^,^58^ (Fig. 6). Initially, triethylamine promoted condensation of isatin with malononitrile could afford isatylidene malononitrile (**A**). An isoquinolinium ylide was generated *in situ* from basic deprotonation of the isoquinolinium salt. Secondly, Michael addition of the isoquinolinium ylide to isatylidene malononitrile (**A**) resulted in intermediate (**B**). Thirdly, the intramolecular coupling of the cyclic iminium ion with the carbanion to give the spiro compound **1**. On the other hand, the spiro compound **1** might be directly formed by the concerted addition reaction of isoquinolinium ylide with isatylidene malononitrile (**A**). In case of reaction with *N-*(4-nitrobenzyl) and *N*-ethoxycarbonylmethyl isoquinolinium salts, the stable spiro compound **1** was separated out as the final product. In the case of *N*-cyanomethylisoquinolinium salt, further nucleophilic addition of the carbanion of malononitrile to the spiro compound **1** afforded a new intermediate (**C**). Then, the intramolecular attack of one cyano group in intermediate (**C**) to the cyclic imine afforded the obtained polycyclic spiro compound **2**.

**Figure 7:**
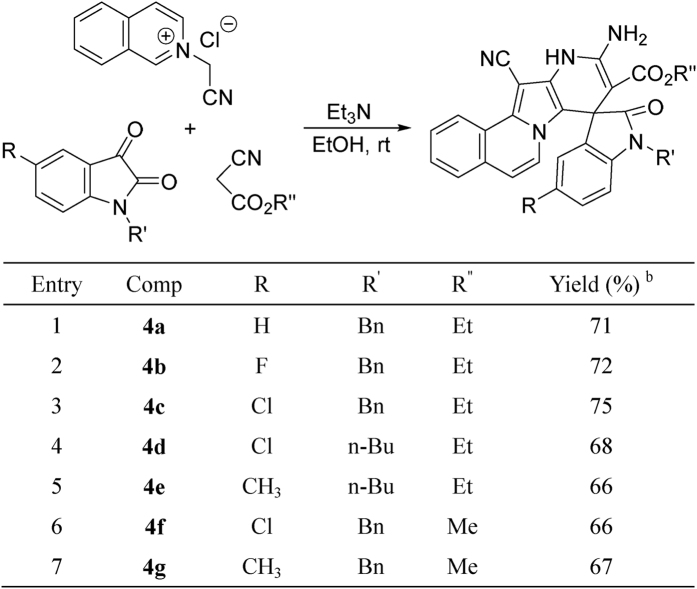
Synthesis of spiro[indoline-3,8′-pyrido[2′,3′:4,5]pyrrolo[2,1-a]isoquinolines] 4a-4g^a^.

**Figure 8:**
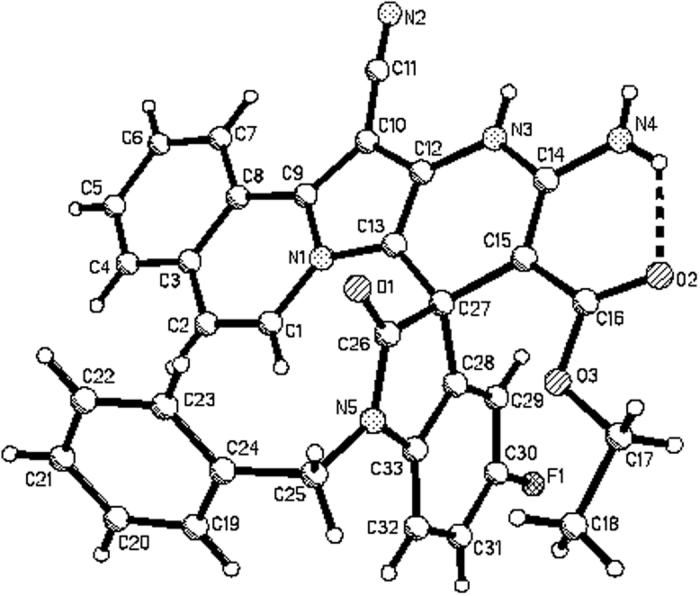
Molecular structure of spiro compound 4b.

Encouraged by the above results, ethyl cyanoacetate and methyl cyanoacetate were also employed as substrates to replace malononitrile under same reaction conditions. Instead of giving spiro[indoline-3,2′-pyrrolo[2,1-*a*]isoquinolines], new types of polycyclic compounds **4a-4g** were produced in moderate to good yields (Fig. 7). In order to elucidate the structures of the polycyclic compounds **4a-4g**, four single crystal structures of **4b** (Fig. 8), **4c**, **4d** and **4e** (Fig. s6–s8 in SI) were successfully determined by X-ray diffraction. From Fig. 8, it can be seen that the neutral compounds **4a-4g** have similar structuralfeatures as zwitterionic compounds **3a-3b**, which suggested that they were produced according to an alternative reaction process.

**Figure 9:**
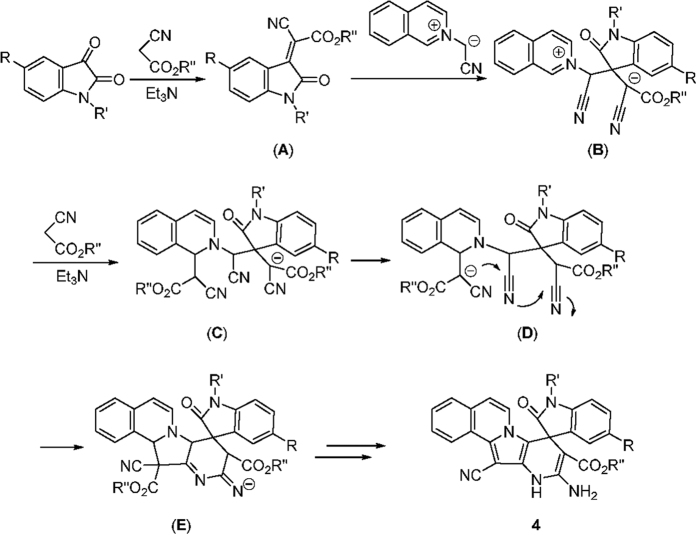
Proposed formation mechanism for spiro compound 4.

Although an accurate interpretation of the reaction mechanism remains elusive, according to the experimental observations and the closely related reports^84^, a plausible mechanism for the formation of polycyclic compounds **4** are proposed in Fig. 9. Presumably, the initially formed isatylidene cyanoacetate (**A**) reacts with the isoquinolinium ylide to afford adduct (**B**) as outlined in Fig. 6 Subsequently a Michael addition of second molecule of ethyl cyanoacetate to the adduct (**B**) provides new intermediate (C), which was converted to the intermediate (**D**). Then, the polycyclic intermediate (**E**) could be formed by an annulation process, which ultimately provides. polycyclic product **4**.

## Conclusion

In summary, we have systematically investigated the three-component reaction of various isoquinolinium salts with isatin and malononitrile or ethyl cyanoacetate. The reaction provided a variety of products depending on the structures of the cyclic nitrogen ylides and the functionalized groups in the substrates, from which the expected functionalized spiro[indoline-3,2′-pyrrolo[2,1-*a*]isoquinolines] and several complex polycyclic spirooxindoles were successfully synthesized in good yields. Possible formation mechanisms accounting for the formation of these complex spiro compounds have been proposed. This protocol has advantages of the mild reaction conditions, easily accessible starting materials, broad substrate scope, satisfactory yields and high diastereoselectivity, which makes it a useful and attractive method for the synthesis of the complex heterocyclic spirooxindole systems in synthetic and medicinal chemistry.

## Methods

### Materials

All reactions were performed in atmosphere unless noted. All reagents were commercially available and use as supplied without further purification. NMR spectra were collected on either an Agilent DD2400 MHz spectrometer or a Bruker AV-600 MHz spectrometer with internal standard tetramethylsilane (TMS) and signals as internal references, and the chemical shifts (δ) were expressed in ppm. High-resolution Mass (ESI) spectra were obtained with Bruker Micro-TOF spectrometer. The Fourier transform infrared (FTIR) samples were prepared as thin films on KBr plates, and spectra were recorded on a Bruker Tensor 27 spectrometer and are reported in terms of frequency of absorption (cm^−1^). X-ray data were collected on a Bruker Smart APEX-2 CCD diffractometer.

### General procedure for the three-component reaction of N-4-nitrobenzyl and *N*-ethoxycarbonylmethylisoquinolinium salts with isatin and malononitrile

To a 50 mL round flask was added *N*-(4-nitrobenzyl) or *N*-ethoxycarbonylmethyl isoquinolinium salt (1.0 mmol), isatin (1.0 mmol), malononitrile (1.0 mmol) and triethylamine (2.0 mmol) in ethanol (15.0 mL). The solution was stirred at room temperature for twelve hours. The resulting precipitates were collected by filtration, which were washed with cold ethanol to give the pure products for analysis.

### General procedure for the three-component reaction of *N*-cyanomethylisoquinolinium chloride with isatin and malononitrile

A mixture of *N*-cyanomethylisoquinolinium chloride (1.0 mmol), isatin (1.0 mmol), malononitrile (2.2 mmol) and triethylamine (2.0 mmol) in ethanol (15.0 mL). The solution was stirred at room temperature for twelve hours. The resulting precipitates were collected by filtration, which were subjected to preparative thin-layer chromatography with a mixture of light petroleum and ethyl acetate (V/V = 3:1) to give the pure products **2a-2l** and **3a-3b** for analysis.

### General procedure for the three-component reaction of *N*-cyanomethylisoquinolinium chloride with isatin and alkyl cyanoacetate

**A** mixture of *N*-cyanomethylisoquinolinium chloride (1.0 mmol), isatin (1.0 mmol), methyl or ethyl cyanoacetate (2.0 mmol) and triethylamine (2.0 mmol) in ethanol (15.0 mL) was stirred at room temperature for twelve hours. The resulting precipitates were collected by filtration, which were subjected to preparative thin-layer chromatography with a mixture of light petroleum and ethyl acetate (V/V = 3:1) to give the pure products **4a-4g** for analysis.

## Additional Information

**How to cite this article**: Sun, J. *et al*. Formation of diverse polycyclic spirooxindoles via three-component reaction of isoquinolinium salts, isatins and malononitrile. *Sci. Rep.* **7**, 41024; doi: 10.1038/srep41024 (2017).

**Publisher's note:** Springer Nature remains neutral with regard to jurisdictional claims in published maps and institutional affiliations.

## Supplementary Material

Supplementary Information
