# Supplementary material for: Formation of diverse polycyclic spirooxindoles via three-component reaction of isoquinolinium salts, isatins and malononitrile
Source: Sci Rep. 2017 Jan 20;7:41024. doi: 10.1038/srep41024 (PMC5247726; doi:10.1038/srep41024)

Formation of diverse polycyclic spirooxindoles via three-component reaction of isoquinolinium salts, isatins and malononitrile

Jing Sun, Guo-liang Shen, Ying Huang, Chao-Guo Yan*

Supporting Information

Figures s1-s8 for molecular structure of compounds 2-4

Characterization data for all compounds 5-16

^{1}H and ^{13}C NMR spectra of the spiro compounds 17-52

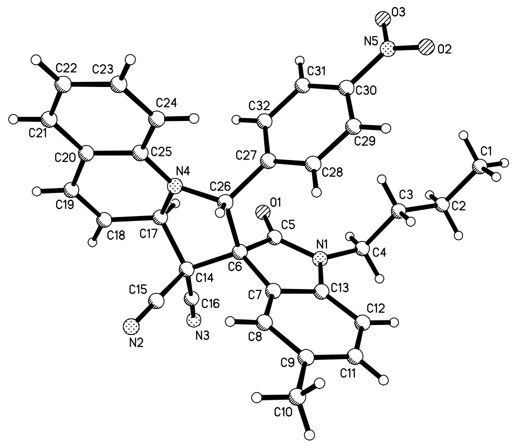

Fig. s1 Molecular structure of compound **1c**

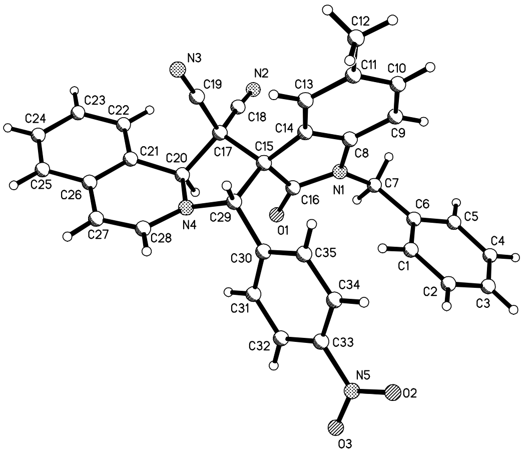

Fig. s2 Molecular structure of Compound **1e**

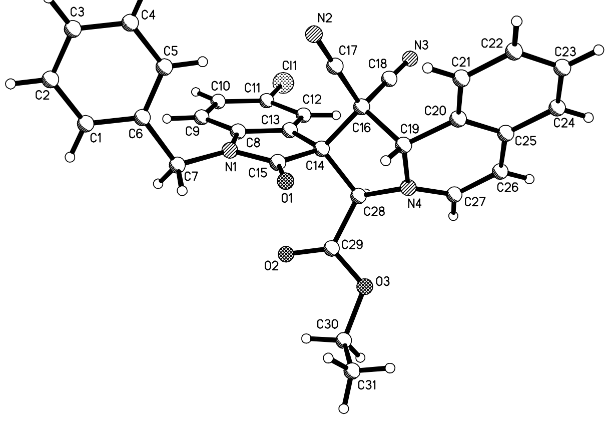

Fig. s3 Molecular structure of Compound **1j**

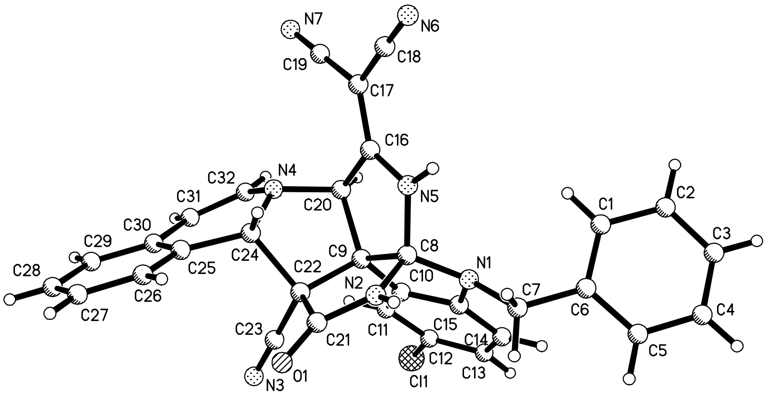

Fig. s4 Molecular structure of compound **2e**

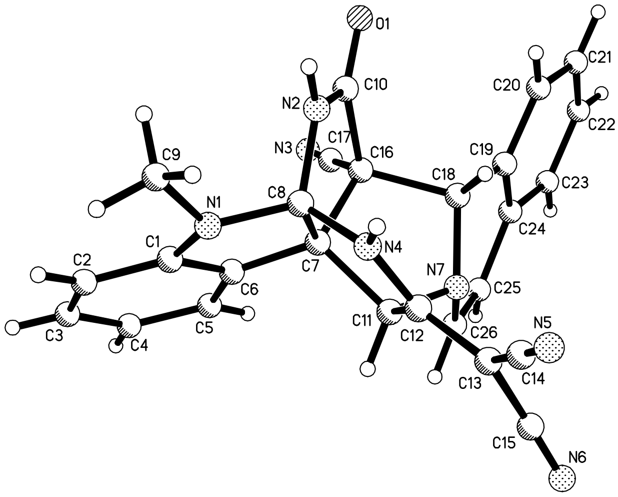

Fig. s5 Molecular structure of compound **2k**

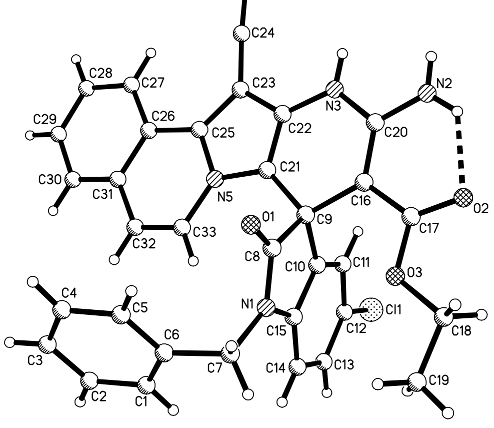

Fig. s6 Molecular structure of compound **4c**

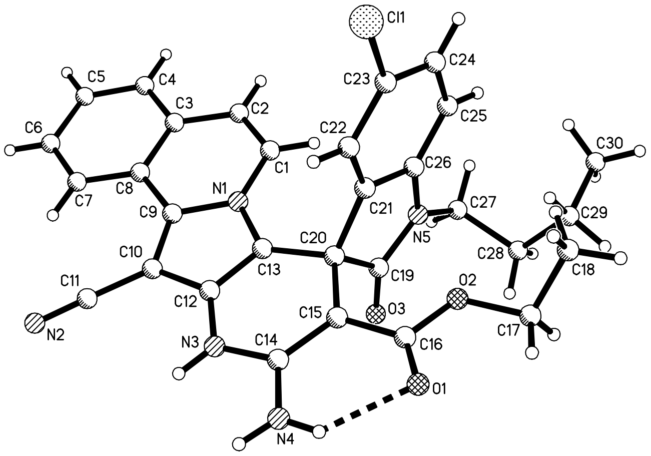

Fig. s7 Molecular structure of compound **4d**

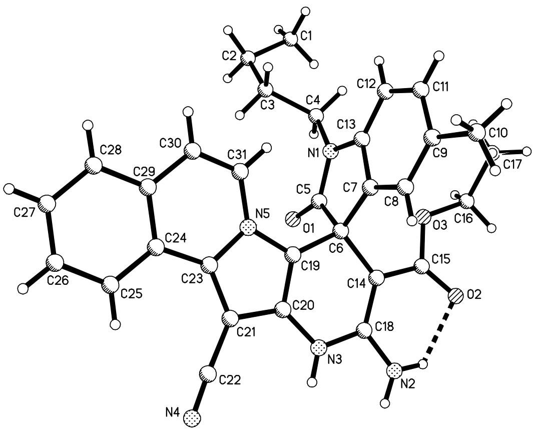

Fig. s8. Molecular structure of compound **4e**

1-Butyl-5-chloro-1',1'-diisocyano-3'-(4-nitrophenyl)-1',10b'-dihydro-3'H-spiro[indoline-3,2'-pyrrolo[2,1-a]isoquinolin]-2-one (1a): Yellow solid, yield: 84%, m.p. 168.4-168.6 ^{o}C, IR (KBr) ν: 3450, 2960, 2869, 1717, 1636, 1607, 1520, 1487, 1456, 1430, 1348, 1280, 1246, 1213, 1188, 1111, 1013, 941, 862, 824, 773, 694, cm^{-1}; ^{1}H NMR (600 MHz, DMSO-d_6) δ: 8.17 (d, J = 7.8 Hz, 2H, ArH), 8.09 (s, 1H, ArH), 7.68 (d, J = 8.4 Hz, 1H, ArH), 7.40 (t, J = 7.8 Hz, 2H, ArH), 7.34（d, J = 7.8 Hz, 1H, ArH), 7.30 (d, J = 8.4 Hz, 1H, ArH), 7.27 (t, J = 7.2 Hz, 2H, ArH), 7.20 (d, J = 7.8 Hz, 1H, ArH), 6.78 (s, 1H, CH), 6.30 (d, J = 7.2 Hz, 1H, CH), 6.13 (s, 1H, CH), 5.54 (d, J = 7.2 Hz, 1H, CH), 3.54-3.51 (m, 1H, CH), 3.49-3.45 (m, 1H, CH), 1.15-1.12 (m, 1H, CH), 1.04-1.01 (m, 1H, CH), 0.82-0.80 (m, 1H, CH), 0.74-0.72 (m, 1H, CH), 0.60 (t, J = 7.2 Hz, 3H, CH_3); ^{13}C NMR (150 MHz, DMSO-d_6) δ: 170.3, 147.5, 142.9, 142.3, 134.0, 132.2, 131.7, 130.2, 127.7, 127.6, 126.6, 125.9, 125.6, 125.2, 123.6, 123.2, 123.1, 111.7, 111.6, 99.9, 70.7, 67.4, 59.3, 49.6, 28.6, 18.9, 13.3; HRMS (ESI) Calcd. for $C_{31}H_{25}ClN_5O_3$ ([M+H]$^+$): 550.1640. Found: 550.1636.

1-Benzyl-5-chloro-1',1'-diisocyano-3'-(4-nitrophenyl)-1',10b'-dihydro-3'H-spiro[indoline-3,2'-pyrrolo[2,1-a]isoquinolin]-2-one (1b): Yellow solid, yield: 90%, m.p. 180.4-180.7 ^{o}C, IR (KBr) ν: 3451, 2860, 1715, 1621, 1522, 1486, 1457, 1434, 1346, 1310, 1248, 1178, 1135, 1083, 930, 863, 814, 772, 741, 695 cm^{-1}; ^{1}H NMR (600 MHz, DMSO-d_6) δ (ppm): 8.11 (t, J = 8.4 Hz, 3H, ArH), 7.64 (d, J = 8.4 Hz, 1H, ArH), 7.40 (d, J = 7.2 Hz, 1H, ArH), 7.38 (d, J = 7.2 Hz, 1H, ArH), 7.30 (d, J = 7.8 Hz, 1H, ArH), 7.26 (d, J = 7.8 Hz, 2H, ArH), 7.21 (d, J = 7.2 Hz, 1H, ArH), 7.16 (t, J = 9.0 Hz, 2H, ArH), 7.07 (t, J = 7.2 Hz, 2H, ArH), 6.84 (s, 1H, CH), 6.77 (d, J = 6.6 Hz, 2H, ArH), 6.29 (d, J = 7.2 Hz, 1H, CH), 6.17 (s, 1H, CH), 5.55 (d, J = 7.2 Hz, 1H, CH), 4.83 (d, J = 15.6 Hz, 1H, CH), 4.71 (d, J = 15.6 Hz, 1H, CH); ^{13}C NMR (150 MHz, DMSO-d_6) δ: 170.5, 147.6, 142.6, 141.8, 134.5, 133.9, 132.2, 131.7, 130.2, 128.8, 128.6, 128.1, 127.9, 127.8, 127.6, 127.5, 127.1, 126.6, 125.9, 125.8, 125.3, 123.7, 123.1, 112.0, 111.7, 100.0, 70.6, 67.4, 59.5, 49.8, 43.1; HRMS (ESI) Calcd. for C_{34} $H_{23}ClN_5O_3$ ([M+H]$^+$): 584.1484. Found: 584.1482.

1-Butyl-5-methyl-1',1'-diisocyano-3'-(4-nitrophenyl)-1',10b'-dihydro-3'H-spiro[indoline-3,2'-pyrrolo[2,1-a]isoquinolin]-2-one (1c): Yellow solid, yield: 78%, m.p. 169.2-169.3 ^{o}C, IR (KBr) ν: 3451, 3066, 2959, 2871, 1708, 1630, 1603, 1518, 1494, 1457, 1417, 1345, 1247, 1203, 1169, 1107, 1013, 934, 859, 814, 769, 744, 688 cm^{-1}; ^{1}H NMR (600 MHz, DMSO-d_6) δ: 8.14 (d, J = 7.8 Hz, 2H, ArH), 7.84 (s, 1H, ArH), 7.41-7.37 (m, 2H, ArH), 7.33 (d, J = 7.2 Hz, 1H, ArH), 7.27（t, J = 7.2 Hz, 1H, ArH), 7.22 (d, J = 8.4 Hz, 2H, ArH), 7.19 (d, J = 7.8 Hz, 1H, ArH), 7.12 (d, J = 7.2 Hz, 1H, ArH), 6.81 (s, 1H, CH), 6.31 (d, J = 7.2 Hz, 1H, CH), 5.93 (s, 1H, CH), 5.51 (d, J = 7.8 Hz, 1H, CH), 3.51-3.48 (m, 1H, CH), 3.43-3.40 (m, 1H, CH), 2.43 (s, 3H, CH_3), 1.15-1.13 (m, 1H, CH), 1.05-1.04 (m, 1H, CH), 0.83-0.81 (m, 1H, CH), 0.74-0.73 (m, 1H, CH), 0.61 (t, J = 7.2 Hz, 3H, CH_3); ^{13}C NMR (150 MHz, DMSO-d_6) δ: 170.5, 147.5, 143.1, 141.0, 134.1, 132.8, 132.2, 132.0, 130.1, 127.7, 126.5, 125.8, 125.7, 125.2, 123.5, 123.3, 121.3, 111.9,

109.9, 99.7, 71.1, 67.0, 59.3, 49.7, 28.7, 20.9, 19.0, 13.3; HRMS (ESI) Calcd. for $C_{32}H_{28}N_5O_3$ ($[M+H]^+$): 530.2187. Found: 530.2182.

5-Methyl-1',1'-diisocyano-3'-(4-nitrophenyl)-1',10b'-dihydro-3'H-spiro[indoline-3,2'-pyrrolo[2,1-a]isoquinolin]-2-one (1d): Yellow solid, yield: 81%, m.p. 186.1-186.5 °C, IR (KBr) ν: 3355, 3064, 2905, 1732, 1633, 1521, 1493, 1457, 1419, 1348, 1279, 1246, 1207, 1160, 1108, 1013, 931, 858, 816, 766, 715, 694, cm^{-1}; ^1H NMR (600 MHz, DMSO-d_6) δ: 11.1 (s, 1H, NH), 8.16 (d, J = 8.4 Hz, 2H, ArH), 7.76 (brs, 1H, ArH), 7.39 (t, J = 7.2 Hz, 1H, ArH), 7.33 (d, J = 7.8 Hz, 1H, ArH), 7.31 (d, J = 7.8 Hz, 1H, ArH), 7.27 (d, J = 7.8 Hz, 1H, ArH), 7.24 (d, J = 8.4 Hz, 2H, ArH), 7.19 (d, J = 7.8 Hz, 1H, ArH), 6.86 (d, J = 7.8 Hz, 1H, ArH), 6.70 (s, 1H, CH), 6.34 (d, J = 7.8 Hz, 1H, CH), 5.91 (s, 1H, CH), 5.51 (d, J = 7.2 Hz, 1H, CH), 2.40 (s, 3H, CH_3); ^{13}C NMR (150 MHz, DMSO-d_6) δ: 172.6, 147.4, 143.7, 140.0, 134.3, 132.3, 132.1, 131.9, 130.1, 127.4, 126.5, 126.0, 125.8, 125.2, 124.3, 123.6, 123.4, 123.3, 122.4, 112.0, 110.5, 99.6, 71.1, 66.9, 59.7, 49.9, 45.7, 20.9; HRMS (ESI) Calcd. for $C_{28}H_{20}N_5O_3$ ($[M+H]^+$): 474.1561. Found: 474.1557.

1-Benzyl-5-methyl-1',1'-diisocyano-3'-(4-nitrophenyl)-1',10b'-dihydro-3'H-spiro[indoline-3,2'-pyrrolo[2,1-a]isoquinolin]-2-one (1e): Yellow solid, yield: 88%, m.p. 185.2-185.4 °C, IR (KBr) ν = 3452, 3063, 2917, 2862, 1712, 1628, 1603, 1520, 1494, 1454, 1416, 1348, 1313, 1284, 1245, 1223, 1198, 1158, 1104, 1035, 997, 933, 863, 820, 763, 738, 690, 624 cm^{-1}; ^1H NMR (600 MHz, DMSO-d_6) δ: 8.08 (d, J = 7.8 Hz, 2H, ArH), 7.86 (s, 1H, ArH), 7.40-7.33 (m, 3H, ArH), 7.28 (t, J = 7.2 Hz, 1H, ArH), 7.23-7.19 (m, 3H, ArH), 7.15 (t, J = 7.2 Hz, 1H, ArH), 7.06 (t, J = 7.2 Hz, 2H, ArH), 7.00 (d, J = 7.8 Hz, 1H, ArH), 7.06 (t, J = 7.2 Hz, 2H, ArH), 7.00 (d, J = 7.8 Hz, 1H, ArH), 6.86 (s, 1H, CH), 6.78 (d, J = 7.8 Hz, 2H, ArH), 6.30 (d, J = 7.2 Hz, 1H, CH), 5.97 (s, 1H, CH), 5.52 (d, J = 7.2 Hz, 1H, CH), 4.80 (d, J = 15.0 Hz, 1H, CH), 4.65 (d, J = 15.0 Hz, 1H, CH), 2.41(s, 3H, CH_3); ^{13}C NMR (150 MHz, DMSO-d_6) δ: 170.7, 152.3, 147.5, 142.8, 140.6, 134.9, 134.0, 133.1, 132.2, 132.0, 130.1, 128.7, 128.1, 127.8, 127.4, 127.1, 126.6, 125.8, 125.2, 123.6, 123.3, 121.1, 120.3, 111.9, 110.3, 99.8, 70.9, 67.0, 59.5, 49.8, 43.0, 20.9; HRMS (ESI) Calcd. for $C_{35}H_{26}N_5O_3$ ($[M+H]^+$): 564.2030. Found: 564.2028.

1-Benzyl-1',1'-diisocyano-3'-(4-nitrophenyl)-1',10b'-dihydro-3'H-spiro[indoline-3,2'-pyrrolo[2,1-a]isoquinolin]-2-one (1f): Yellow solid, yield: 85%, m.p. 193.3-193.5 °C, IR (KBr) ν: 3452, 3064, 2899, 1803, 1711, 1611, 1521, 1490, 1461, 1417, 1373, 1349, 1314, 1283, 1233, 1180, 1130, 1105, 994, 938, 869, 807, 759, 694 cm^{-1}; ^1H NMR (600 MHz, DMSO-d_6) δ: 8.08 (d, J = 8.4 Hz, 2H, ArH), 8.02 (d, J = 7.8 Hz, 1H, ArH), 7.54 (t, J = 7.8 Hz, 1H, ArH), 7.40-7.36 (m, 3H, ArH), 7.29 (t, J = 7.8 Hz, 1H, ArH), 7.21 (t, J = 9.0 Hz, 3H, ArH), 7.15-7.12 (m, 2H, ArH), 7.06 (t, J = 7.2 Hz, 2H, ArH), 6.86 (s, 1H, CH), 6.79 (d, J = 7.2 Hz, 2H, ArH), 6.31 (d, J = 7.8 Hz, 1H, CH), 6.01 (s, 1H, CH), 5.53 (d, J = 7.8 Hz, 1H, CH), 4.83 (d, J = 15.6 Hz, 1H, CH), 4.69 (d, J = 15.6 Hz, 1H, CH); ^{13}C NMR (150 MHz, DMSO-d_6) δ: 170.8, 147.5, 142.9, 142.7, 134.8, 133.8, 132.2, 131.8, 130.1, 128.7, 128.6, 128.1, 127.8, 127.5, 127.4, 127.1, 126.6, 126.4, 125.8, 125.3, 125.2,

123.9, 123.6, 123.3, 121.1, 120.3, 111.9, 110.5, 99.9, 70.9, 67.0, 59.6, 49.7, 43.0; HRMS (ESI) Calcd. for $C_{34}H_{24}N_5O_3$ ($[M+H]^+$): 550.1874. Found: 550.1871.

1-Butyl-5-fluoro-1',1'-diisocyano-3'-(4-nitrophenyl)-1',10b'-dihydro-3'H-spiro[indoline-3,2'-pyrrolo[2,1-a]isoquinolin]-2-one (1g): Yellow solid, yield: 83%, m.p. 173.1-173.5 °C, IR (KBr) ν: 3452, 2955, 2867, 2025, 1721, 1693, 1526, 1492, 1454, 1417, 1350, 1278, 1191, 1104, 1014, 946, 832, 779, 688 cm^{-1}; ^{1}H NMR (600 MHz, DMSO-d_6) δ: 8.16 (d, J = 7.8 Hz, 2H, ArH), 7.90 (d, J = 7.8 Hz, 1H, ArH), 7.48 (t, J = 8.4 Hz, 1H, ArH), 7.39 (d, J = 6.6 Hz, 1H, ArH), 7.34 (t, J = 7.2 Hz, 1H, ArH), 7.28 (t, J = 7.8 Hz, 4H, ArH), 7.20 (d, J = 7.8 Hz, 1H, ArH), 6.81 (s, 1H, CH), 6.28 (d, J = 6.6 Hz, 1H, CH), 6.06 (s, 1H, CH), 5.53 (d, J = 6.6 Hz, 1H, CH), 3.54-3.52 (m, 1H, CH), 3.47-3.45 (m, 1H, CH), 1.15 (brs, 1H, CH), 1.05-1.04 (m, 1H, CH), 0.82 (brs, 1H, CH), 0.74-0.73 (m, 1H, CH), 0.62 (t, J = 7.2 Hz, 3H, CH_3); ^{13}C NMR (150 MHz, DMSO-d_6) δ(ppm): 170.4, 158.4(d, J = 239.0 Hz), 147.6, 142.8, 139.7, 133.9, 132.1, 130.2, 127.8, 126.6, 125.8, 125.2, 123.6, 123.2, 122.8 (d, J = 8.0 Hz), 118.3 (d, J = 22.8 Hz), 113.5 (d, J = 26.1 Hz), 111.7, 111.4 (d, J = 8.0 Hz), 99.9, 70.8, 67.2, 59.4, 49.5, 28.6, 19.0, 13.3; HRMS (ESI) Calcd. for $C_{31}H_{25}FN_5O_3$ ($[M+H]^+$): 534.1936. Found: 534.1932.

1-Benzyl-5-fluoro-1',1'-diisocyano-3'-(4-nitrophenyl)-1',10b'-dihydro-3'H-spiro[indoline-3,2'-pyrrolo[2,1-a]isoquinolin]-2-one (1h): Yellow solid, yield: 87%, m.p. 175.6-175.9 °C, IR (KBr) ν: 3452, 3065, 1716, 1627, 1523, 1492, 1453, 1417, 1349, 1314, 1276, 1249, 1227, 1181, 1106, 1033, 962, 938, 855, 822, 765, 724, 692 cm^{-1}; ^{1}H NMR (600 MHz, DMSO-d_6) δ: 8.10 (d, J = 8.4 Hz, 2H, ArH), 7.93 (d, J = 8.4 Hz, 1H, ArH), 7.40-7.38 (m, 3H, ArH), 7.30 (d, J = 7.8 Hz, 1H, ArH), 7.27 (d, J = 8.4 Hz, 2H, ArH), 7.21 (d, J = 7.2 Hz, 1H, ArH), 7.15 (d, J = 7.2 Hz, 2H, ArH), 7.07 (t, J = 7.2 Hz, 2H, ArH), 6.87 (s, 1H, CH), 6.78 (d, J = 7.2 Hz, 2H, ArH), 6.28 (d, J = 7.8 Hz, 1H, CH), 6.11 (s, 1H, CH), 5.55 (d, J = 7.8 Hz, 1H, CH), 4.83 (d, J = 15.6 Hz, 1H, CH), 4.70 (d, J = 15.6 Hz, 1H, CH); ^{13}C NMR (150 MHz, DMSO-d_6) δ: 170.6, 158.4(d, J = 239.3 Hz), 147.6, 142.5, 139.2, 134.7, 133.8, 132.1, 130.2, 128.8, 128.1, 127.9, 127.3, 127.1, 126.6, 125.8, 125.2, 123.7, 123.2, 122.7 (d, J = 8.1 Hz), 118.4 (d, J = 23.0 Hz), 113.7 (d, J = 26.6 Hz), 111.7 (d, J = 8.4 Hz), 111.7, 100.0, 70.6, 67.2, 59.7, 49.7, 43.1; HRMS (ESI) Calcd. for $C_{34}H_{23}FN_5O_3$ ($[M+H]^+$): 568.1779. Found: 568.1778.

Ethyl 1-benzyl-1',1'-diisocyano-2-oxo-1',10b'-dihydro-3'H-spiro[indoline-3,2'-pyrrolo[2,1-a]isoquinoline]-3'-carboxylate (1i): White solid, yield: 62%, m.p. 145.5-145.7 °C, IR (KBr) ν: 3443, 2987, 2935, 2025, 1729, 1705, 1643, 1491, 1462, 1421, 1371, 1345, 1306, 1270, 1250, 1207, 1172, 1133, 1107, 1027, 986, 938, 861, 758, 735, 691 cm^{-1}; ^{1}H NMR (600 MHz, $CDCl_3$) δ: 7.70 (d, J = 7.8 Hz, 1H, ArH), 7.44 (d, J = 7.2 Hz, 2H, ArH), 7.36 (t, J = 7.8 Hz, 4H, ArH), 7.32-7.30 (m, 2H, ArH), 7.20 (d, J = 7.8 Hz, 1H, ArH), 7.18 (d, J = 7.8 Hz, 1H, ArH), 7.10 (d, J = 7.2 Hz, 1H, ArH), 6.83 (d, J = 8.4 Hz, 1H, ArH), 6.48 (s, 1H, CH), 6.23 (d, J = 7.8 Hz, 1H, CH), 5.50 (d, J = 7.8 Hz, 1H, CH), 5.14 (d, J = 15.6 Hz, 1H, CH), 4.83 (d, J = 15.6 Hz, 1H, CH), 4.69 (s, 1H,

CH), 4.18-4.15 (m, 1H, CH), 4.09-4.06 (m, 1H, CH), 1.13 (t, J = 7.2 Hz, 3H, CH_3); ^{13}C NMR (150 MHz, $CDCl_3$) δ: 173.2, 170.0, 143.6, 134.5, 133.5, 132.2, 131.1, 130.2, 128.9, 128.1, 127.7, 127.5, 126.8, 126.6, 125.5, 124.5, 123.8, 123.3, 110.4, 101.1, 72.0, 68.4, 62.5, 58.1, 51.0, 44.8, 13.9; HRMS (ESI) Calcd. for $C_{31}H_{25}N_4O_3$ ($[M+H]^+$): 501.1921. Found: 501.1922.

Ethyl 1-butyl-5-chloro-1',1'-diisocyano-2-oxo-1',10b'-dihydro-3'H-spiro[indoline-3,2'-pyrrolo[2,1-a]isoquinoline]-3'-carboxylate (**1j**): White solid, yield: 70%, m.p. 147.3-147.9 °C, IR (KBr) ν: 3446, 2971, 2025, 1712, 1632, 1487, 1456, 1430, 1369, 1302, 1252, 1186, 1162, 1111, 1034, 940, 858, 816, 774, 742, 680 cm^{-1}; ^{1}H NMR (600 MHz, $CDCl_3$) δ: 7.67 (brs, 1H, ArH), 7.42 (d, J = 7.8 Hz, 2H, ArH), 7.32 (s, 1H, ArH), 7.20 (brs, 1H, ArH), 7.10 (brs, 1H, ArH), 6.91 (brs, 1H, ArH), 6.42 (s, 1H, CH), 6.20 (d, J = 7.2 Hz, 1H, CH), 5.50 (d, J = 7.2 Hz, 1H, CH), 4.62 (s, 1H, CH), 4.15 (brs, 2H, CH_2), 3.86 (brs, 2H, CH_2), 1.71(brs, 2H, CH_2), 1.45(brs, 2H, CH_2), 1.18(s, 3H, CH_3), 0.98(brs, 3H, CH_3); ^{13}C NMR (150 MHz, DMSO-d_6) δ: 172.6, 167.8, 142.6, 133.4, 132.1, 132.0, 131.2, 130.2, 129.0, 126.8, 126.5, 125.6, 125.1, 123.2, 111.4, 110.4, 101.2, 71.8, 68.4, 62.5, 57.7, 50.7, 50.6, 40.8, 29.1, 20.2, 13.9, 13.6; HRMS (ESI) Calcd. for $C_{28}H_{26}Cl\ N_4O_3$ ($[M+H]^+$): 501.1688. Found: 501.1692.

Ethyl 1-benzyl-5-chloro-1',1'-diisocyano-2-oxo-1',10b'-dihydro-3'H-spiro[indoline-3,2'-pyrrolo[2,1-a]isoquinoline]-3'-carboxylate (1k): White solid, yield: 72%, m.p. 142.1-142.5 °C, IR (KBr) ν: 3442, 2980, 2025, 1721, 1633, 1609, 1488, 1454, 1431, 1368, 1305, 1241, 1176, 1133, 1109, 1027, 968, 936, 861, 813, 773, 735, 698, 677 cm^{-1}; ^{1}H NMR (600 MHz, $CDCl_3$) δ: 7.68 (s, 1H, ArH), 7.42 (t, J = 9.0 Hz, 3H, ArH), 7.36 (d, J = 7.8 Hz, 1H, ArH), 7.34 (t, J = 7.2 Hz, 2H, ArH), 7.31 (d, J = 7.2 Hz, 2H, ArH), 7.21 (t, J = 7.2 Hz, 1H, ArH), 7.11 (d, J = 7.2 Hz, 1H, ArH), 6.74 (d, J = 7.8 Hz, 1H, ArH), 6.46 (s, 1H, CH), 6.22 (d, J = 7.8 Hz, 1H, CH), 5.51 (d, J = 7.8 Hz, 1H, CH), 5.10 (d, J = 15.6 Hz, 1H, CH), 4.81 (d, J = 15.6 Hz, 1H, CH), 4.67 (s, 1H, CH), 4.20-4.17 (m, 1H, CH), 4.11-4.08 (m, 1H, CH), 1.16 (t, J = 7.2 Hz, 3H, CH_3); ^{13}C NMR (150 MHz, $CDCl_3$) δ: 172.9, 167.9, 142.1, 134.0, 133.3, 132.1, 131.2, 130.3, 129.0, 128.2, 127.6, 126.9, 126.6, 125.7, 125.4, 125.0, 123.1, 111.4, 101.4, 72.2, 68.6, 62.7, 58.0, 50.8, 44.9, 14.0; HRMS (ESI) Calcd. for $C_{31}H_{24}ClN_4O_3$ ($[M+H]^+$): 535.1531. Found: 535.1537.

Ethyl 1-butyl-5-methyl-1',1'-diisocyano-2-oxo-1',10b'-dihydro-3'H-spiro[indoline-3,2'-pyrrolo[2,1-a]isoquinoline]-3'-carboxylate (**1l**): White solid, yield: 86%, m.p. 156.8-157.0 °C, IR (KBr) ν: 3444, 2971, 2865, 2025, 1730, 1704, 1630, 1497, 1454, 1367, 1341, 1301, 1251, 1203, 1156, 1127, 1107, 1035, 989, 938, 861, 815, 774, 744, 687 cm^{-1}; ^{1}H NMR (600 MHz, $CDCl_3$) δ: 7.49 (brs, 1H, ArH), 7.40 (d, J = 7.2 Hz, 1H, ArH), 7.31 (t, J = 7.2 Hz, 1H, ArH), 7.24 (brs, 1H, ArH), 7.18 (t, J = 7.2 Hz, 1H, ArH), 7.08 (d, J = 7.2 Hz, 1H, ArH), 6.86 (d, J = 7.8 Hz, 1H, ArH), 6.44 (s, 1H, CH), 6.21 (d, J = 7.2 Hz, 1H, CH), 5.48 (d, J = 7.2 Hz, 1H, CH), 4.62 (s, 1H, CH), 4.15-4.13 (m, 2H, CH_2), 3.89-3.82 (m, 2H, CH_2), 2.39 (s, 3H, CH_3), 1.75-1.70 (m, 2H, CH_2), 1.48-1.43 (m, 2H, CH_2), 1.16 (d, J = 7.2 Hz, 3H, CH_3), 0.98 (t, J = 7.2 Hz, 3H, CH_3); ^{13}C NMR

(150 MHz, DMSO-d_6) δ: 172.8, 168.0, 141.5, 133.6, 133.3, 132.8, 132.2, 131.5, 130.1, 126.7, 126.6, 125.5, 125.2, 123.8, 123.4, 111.7, 109.1, 100.9, 71.7, 68.2, 62.3, 57.8, 50.9, 40.6, 29.2, 21.2, 20.2, 13.9, 13.7; HRMS (ESI) Calcd. for C_{29} $H_{29}N_4O_3$ ($[M+H]^+$): 481.2234. Found: 481.2237.

Ethyl 1-benzyl-5-methyl-1',1'-diisocyano-2-oxo-1',10b'-dihydro-3'H-spiro[indoline-3,2'-pyrrolo[2,1-a]isoquinoline]-3'-carboxylate (1m): White solid, yield: 80%, m.p. 156.1-156.3 ^oC, IR (KBr) ν: 3446, 3068, 2935, 2244, 2026, 1893, 1826, 1734, 1706, 1630, 1496, 1452, 1371, 1341, 1305, 1250, 1201, 1160, 1134, 1106, 1026, 991, 940, 908, 815, 726, 688 cm^{-1}; ^1H NMR (600 MHz, $CDCl_3$) δ: 7.50 (brs, 1H, ArH), 7.43 (d, J = 7.8 Hz, 2H, ArH), 7.35 (d, J = 7.8 Hz, 2H, ArH), 7.33 (brs, 1H, ArH), 7.32-7.29 (m, 2H, ArH), 7.19 (t, J = 7.2 Hz, 1H, ArH), 7.12 (d, J = 9.0 Hz, 1H, ArH), 7.10 (d, J = 7.2 Hz, 1H, ArH), 6.70 (d, J = 7.8 Hz, 1H, ArH), 6.48 (s, 1H, CH), 6.23 (d, J = 7.8 Hz, 1H, CH), 5.50 (d, J = 7.8 Hz, 1H, CH), 5.10 (d, J = 15.6 Hz, 1H, CH), 4.80 (d, J = 15.6 Hz, 1H, CH), 4.67 (s, 1H, CH), 4.17-4.14 (m, 1H, CH), 4.09-4.06 (m, 1H, CH), 2.53 (s, 3H, CH_3), 1.13 (t, J = 7.2 Hz, 3H, CH_3); ^{13}C NMR (150 MHz, $CDCl_3$) δ: 173.2, 168.1, 141.2, 134.6, 133.6, 132.9, 132.2, 131.5, 130.2, 128.9, 128.0, 127.7, 127.6, 126.8, 126.6, 125.5, 125.2, 123.7, 123.4, 110.2, 101.0, 72.1, 68.5, 62.5, 58.2, 51.0, 44.8, 21.2, 14.0; HRMS (ESI) Calcd. for $C_{32}H_{27}N_4O_3$ ($[M+H]^+$): 515.2078. Found: 515.2080.

Ethyl 5-methyl-1',1'-diisocyano-2-oxo-1',10b'-dihydro-3'H-spiro[indoline-3,2'-pyrrolo[2,1-a]isoquinoline]-3'-carboxylate (1n): White solid, yield: 53%, m.p. 143.2-143.5 ^oC, IR (KBr) ν: 3429, 2978, 1717, 1628, 1494, 1451, 1418, 1370, 1305, 1252, 1208, 1156, 1104, 1028, 991, 938, 884, 861, 824, 776, 740, 686 cm^{-1}; ^1H NMR (600 MHz, $CDCl_3$) δ: 8.60 (s, 1H, NH), 7.47 (s, 1H, ArH), 7.42 (d, J = 7.8 Hz, 1H, ArH), 7.32 (t, J = 7.8 Hz, 1H, ArH), 7.20 (d, J = 7.2 Hz, 2H, ArH), 7.10 (d, J = 7.2 Hz, 1H, ArH), 6.91 (d, J = 7.8 Hz, 1H, ArH), 6.37 (s, 1H, CH), 6.23 (d, J = 7.2 Hz, 1H, CH), 5.50 (d, J = 7.2 Hz, 1H, CH), 4.65 (s, 1H, CH), 3.73 (q, J = 7.8 Hz, 2H, CH_2), 2.38 (s, 3H, CH_3), 1.25 (t, J = 7.2 Hz, 3H, CH_3); ^{13}C NMR (150 MHz, $CDCl_3$) δ: 175.2, 168.0, 139.0, 133.6, 133.5, 132.2, 131.7, 130.2, 126.7, 126.5, 125.6, 125.3, 124.2, 123.3, 110.8, 101.1, 71.9, 68.4, 62.5, 58.4, 50.8, 21.3, 18.4, 13.8; HRMS (ESI) Calcd. for $C_{25}H_{21}N_4O_3$ ($[M+H]^+$): 425.1608. Found: 425.1611.

Ethyl 1-butyl-5-fluoro-1',1'-diisocyano-2-oxo-1',10b'-dihydro-3'H-spiro[indoline-3,2'-pyrrolo[2,1-a]isoquinoline]-3'-carboxylate (1o): White solid, yield: 76%, m.p. 145.4-145.6 ^oC, IR (KBr) ν: 3448, 2972, 2867, 2025, 1707, 1631, 1492, 1453, 1421, 1367, 1302, 1273, 1191, 1164, 1134, 1107, 1034, 939, 863, 823, 774, 742, 686, 638 cm^{-1}; ^1H NMR (600 MHz, $CDCl_3$) δ: 7.45 (d, J = 7.2 Hz, 1H, ArH), 7.40 (d, J = 7.8 Hz, 1H, ArH), 7.32 (t, J = 7.2 Hz, 1H, ArH), 7.20 (d, J = 7.8 Hz, 1H, ArH), 7.16 (d, J = 7.8 Hz, 1H, ArH), 7.10 (d, J = 7.2 Hz, 1H, ArH), 6.93-6.90 (m, 1H, ArH), 6.44 (s, 1H, CH), 6.20 (d, J = 7.2 Hz, 1H, CH), 5.50 (d, J = 7.2 Hz, 1H, CH), 4.61 (s, 1H, CH), 4.15 (q, J = 7.8 Hz, 2H, CH_2), 3.89-3.84 (m, 2H, CH_2), 1.73-1.71 (m, 2H, CH_2), 1.48-1.44 (m, 2H, CH_2), 1.18 (t, J = 7.2 Hz, 3H, CH_3), 0.99 (t, J = 7.2 Hz, 3H, CH_3); ^{13}C NMR (150 MHz,

DMSO-d_6) δ: 172.7, 167.8, 159.2(d, J = 242.1 Hz), 140.0, 133.4, 132.1, 130.2, 126.8, 126.7, 126.5, 125.6, 123.2, 117.7(d, J = 23.1 Hz), 113.0(d, J = 26.0 Hz), 111.4, 110.1(d, J = 8.0 Hz), 101.2, 71.7, 68.3, 62.5, 57.9, 50.8, 40.8, 29.1, 20.2, 13.9, 13.6; HRMS (ESI) Calcd. for $C_{28}H_{26}FN_4O_3$ ($[M+H]^+$): 485.1983. Found: 485.1994.

15-cyano-16-oxo-14b,15-dihydro-5a,15-(epiminomethano)indolo[2'',3'':2',3']pyrrolo[3',4':4,5]pyrrolo[2,1-a]isoquinolin-7(5H,6H,7aH)-ylidene)malononitrile (2a): white solid, 62%, m.p. 256-258℃; ^{1}H NMR (400 MHz, DMSO) δ: 11.67 (s, 1H, NH), 10.29 (s, 1H, NH), 7.41 (s, 1H, ArH), 7.36-7.32 (m, 1H, ArH), 7.24-7.16 (m, 5H, ArH), 6.85 (t, J = 7.6 Hz, 1H, ArH), 6.71 (d, J = 8.0 Hz, 1H, CH), 6.59 (d, J = 7.6 Hz, 1H, CH), 5.68-5.64 (m, 2H, CH), 5.25 (s, 1H, CH); ^{13}C NMR (150 MHz, DMSO) δ: 171.1, 167.5, 148.9, 134.4, 131.5, 130.6, 129.4, 128.0, 125.9, 125.3, 125.1, 124.7, 122.2, 119.8, 114.8, 114.6, 110.5, 102.1, 99.6, 79.1, 68.5, 68.3, 63.7, 47.0; IR (KBr) υ: 3578, 3402, 3148, 3070, 2976, 2807, 2212, 1724, 1656, 1595, 1483, 1439, 1411, 1379, 1316, 1247, 1154, 1101, 1028, 943, 765 cm^{-1}; MS (m/z): HRMS (ESI) Calcd. for $C_{25}H_{14}N_7O$ ($[M-H]^-$): 428.1265. Found: 428.1270.

15-cyano-2-fluoro-16-oxo-14b,15-dihydro-5a,15-(epiminomethano)indolo[2'',3'':2',3']pyrrolo[3',4':4,5]pyrrolo[2,1-a]isoquinolin-7(5H,6H,7aH)-ylidene)malononitrile (2b): white solid, 58%, m.p. 257-259℃; ^{1}H NMR (400 MHz, DMSO) δ: 11.70 (s, 1H, NH), 10.34 (s, 1H, NH) 7.35-7.11 (m, 7H, ArH), 6.92-6.91 (m, 1H, ArH), 6.71 (m, 1H, CH), 6.61-6.60 (m, 1H, CH), 5.75-5.67 (m, 2H, CH), 5.27 (s, 1H, CH); ^{13}C NMR (150 MHz, DMSO) δ: 174.3, 170.9, 167.7, 167.2, 159.5, 157.9, 156.9, 155.4, 153.8, 145.4, 137.6, 135.2, 134.4, 132.4, 131.5, 129.7, 129.4, 128.0, 126.9, 125.9, 124.7, 122.3, 119.4, 117.1, 114.9, 114.7, 112.4, 112.2, 111.3, 107.7, 102.1, 70.2, 69.8, 68.4, 68.3, 63.4, 60.7, 47.4; IR (KBr) υ: 3633, 3320, 3090, 2922, 2850, 2217, 1721, 1658, 1604, 1490, 1456, 1387, 1297, 1252, 1156, 1104, 982, 931, 822, 770 cm^{-1}; MS (m/z): HRMS (ESI) Calcd. for $C_{25}H_{13}FN_7O$ ($[M-H]^-$): 446.1171. Found: 446.1176.

15-cyano-2-methyl-16-oxo-14b,15-dihydro-5a,15-(epiminomethano)indolo[2'',3'':2',3']pyrrolo[3',4':4,5]pyrrolo[2,1-a]isoquinolin-7(5H,6H,7aH)-ylidene)malononitrile (2c): white solid, 60%, m.p. 270-272℃; ^{1}H NMR (600 MHz, DMSO) δ: 11.58 (s, 1H, NH), 10.18 (s, 1H, NH), 7.33 (brs, 1H, ArH), 7.21-7.11 (m, 4H, ArH), 7.04-7.01 (m, 2H, ArH), 6.62-6.59 (m, 2H, CH), 5.65 (s, 2H, CH), 5.23 (s, 1H, CH), 2.22 (s, 3H, CH_3); ^{13}C NMR (150 MHz, DMSO) δ: 171.0, 167.5, 146.6, 134.4, 131.5, 131.1, 129.3, 128.4, 128.0, 125.8, 125.6, 125.1, 124.6, 122.5, 114.8, 114.7, 114.6, 110.6, 101.9, 99.9, 78.8, 68.4, 68.3, 68.2, 63.6, 56.0, 46.8, 46.8, 20.5, 18.5; IR (KBr) υ: 3559, 3495, 3240, 2227, 1725, 1603, 1496, 1461, 1397, 1298, 1243, 1152, 1109, 1063, 972, 880, 849, 819, 770 cm^{-1}; MS (m/z): HRMS (ESI) Calcd. for $C_{26}H_{16}N_7O$ ($[M-H]^-$): 442.1422. Found: 442.1422.

5-benzyl-15-cyano-16-oxo-14b,15-dihydro-5a,15-(epiminomethano)indolo[2'',3'':2',3']pyrrolo[3',4':4,5]pyrrolo[2,1-a]isoquinolin-7(5H,6H,7aH)-ylidene)malononitrile (2d): white solid, 61%, m.p. 234-236℃; ^{1}H NMR (600 MHz, DMSO) δ: 11.92 (s, 1H, NH), 10.74 (s, 1H, NH), 7.38-7.34 (m, 3H, ArH), 7.29-7.18 (m, 7H, ArH), 7.09 (m, 1H, ArH), 6.81 (m, 1H, ArH), 6.60 (d, *J* = 7.2 Hz, 1H, ArH), 6.05 (d, *J* = 7.2 Hz, 1H, CH), 5.84 (s, 1H, CH), 5.68 (d, *J* = 6.2 Hz, 1H, CH), 5.35 (s, 1H, CH), 4.91 (d, *J* = 16.8 Hz, 1H, CH), 4.56 (d, *J* = 16.8 Hz, 1H, CH); ^{13}C NMR (150 MHz, DMSO) δ: 171.6, 167.5, 148.4, 137.5, 134.4, 131.5, 130.5, 129.4, 128.5, 128.1, 127.0, 126.5, 125.9, 125.2, 124.7, 121.9, 119.0, 114.6, 107.7, 102.2, 79.2, 68.3, 67.3, 64.3, 47.2; IR (KBr) υ: 3546, 3070, 2855, 2216, 1722, 1658, 1605, 1490, 1455, 1379, 1319, 1156, 1108, 974, 748 cm^{-1}; MS (m/z): HRMS (ESI) Calcd. for $C_{32}H_{20}N_7O$ ([M-H]$^-$): 518.1735. Found: 518.1739.

5-benzyl-2-chloro-15-cyano-16-oxo-14b,15-dihydro-5a,15-(epiminomethano)indolo[2'',3'':2',3']pyrrolo[3',4':4,5]pyrrolo[2,1-a]isoquinolin-7(5H,6H,7aH)-ylidene)malononitrile (2e): white solid, 62%, m.p. 276-278℃; ^{1}H NMR (400 MHz, DMSO) δ: 12.00 (s, 1H, NH), 10.86 (s, 1H, NH), 7.41-7.34 (m, 3H, ArH), 7.31-7.17 (m, 7H, ArH), 7.14-7.13 (m, 1H, ArH), 6.63 (d, *J* = 7.6 Hz, 1H, ArH), 6.03 (d, *J* = 8.4 Hz, 1H, CH), 5.96 (s, 1H, CH), 5.73 (d, *J* = 7.6 Hz, 1H, CH), 5.40 (s, 1H, CH), 4.90 (d, *J* = 17.0 Hz, 1H, CH), 4.59 (d, *J* = 17.0 Hz, 1H, CH); ^{13}C NMR (150 MHz, DMSO) δ: 171.4, 167.2, 147.2, 136.9, 134.3, 131.5, 130.3, 129.4, 128.6, 128.1, 127.1, 126.5, 126.0, 125.1, 124.8, 123.7, 122.2, 114.5, 108.9, 102.5, 78.9, 68.4, 66.9, 64.2, 47.1; IR (KBr) υ: 3177, 3071, 2874, 2226, 1722, 1611, 1543, 1487, 1458, 1419, 1354, 1327, 1274, 1158, 1107, 1079, 975, 777, 746, 706 cm^{-1}; MS (m/z): HRMS (ESI) Calcd. for $C_{32}H_{19}ClN_7O$ ([M-H]$^-$): 552.1345. Found: 552.1346.

5-benzyl-15-cyano-2-fluoro-16-oxo-14b,15-dihydro-5a,15-(epiminomethano)indolo[2'',3'':2',3']pyrrolo[3',4':4,5]pyrrolo[2,1-a]isoquinolin-7(5H,6H,7aH)-ylidene)malononitrile (2f): white solid, 63%, m.p. 272-274℃; ^{1}H NMR (400 MHz, DMSO) δ: 11.99 (s, 1H, NH), 10.83 (s, 1H, NH), 7.41-7.19 (m, 9H, ArH), 7.02-6.95 (m, 2H, ArH), 6.62 (d, *J* = 7.6 Hz, 1H, ArH), 6.01-5.98 (m, 1H, CH), 5.92 (s, 1H, CH), 5.72 (d, *J* = 7.6 Hz, 1H, CH), 5.39 (s, 1H, CH), 4.89 (d, *J* = 16.8 Hz, 1H, CH), 4.53 (d, *J* = 16.8 Hz, 1H, CH); ^{13}C NMR (150 MHz, DMSO) δ: 171.4, 167.3, 156.5, 154.9, 154.0, 145.0, 137.3, 134.3, 131.5, 129.4, 128.5, 128.1, 127.1, 126.6, 126.0, 125.1, 124.8, 114.6, 112.7, 112.5, 68.3, 67.0, 64.1, 47.4; IR (KBr) υ: 3633, 3223, 3069, 2838, 2219, 1723, 1606, 1492, 1452, 1366, 1322, 1264, 1155, 1106, 982, 917, 815, 747 cm^{-1}; MS (m/z): HRMS (ESI) Calcd. for $C_{32}H_{19}FN_7O$ ([M-H]$^-$): 536.1641. Found: 536.1642.

5-benzyl-15-cyano-2-methyl-16-oxo-14b,15-dihydro-5a,15-(epiminomethano)indolo[2'',3'':2',3']pyrrolo[3',4':4,5]pyrrolo[2,1-a]isoquinolin-7(5H,6H,7aH)-ylidene)malononitrile (2g): white solid, 70%, m.p. 234-236℃; ^{1}H NMR (400 MHz, DMSO) δ: 11.94 (s, 1H, NH), 10.76 (s, 1H, NH), 7.40-7.33 (m, 3H, ArH), 7.29-7.24 (m, 4H, ArH), 7.22-7.17 (m, 2H, ArH), 7.03 (brs, 1H,

ArH), 6.91 (d, J = 8.0 Hz, 1H, ArH), 6.60 (d, J = 7.6 Hz, 1H, ArH), 5.94 (d, J = 8.0 Hz, 1H, CH), 5.86 (s, 1H, CH), 5.70 (d, J = 7.6 Hz, 1H, CH), 5.36 (s, 1H, CH), 4.88 (d, J = 16.8 Hz, 1H, CH), 4.51 (d, J = 16.8 Hz, 1H, CH), 2.18 (s, 3H, CH_3); ^{13}C NMR (100 MHz, DMSO) δ: 172.0, 168. 0, 146.7, 138.1, 134.8, 132.0, 131.3, 129.8, 129.0, 128.5, 128.2, 127.4, 127.0, 126.4, 126.1, 125.6, 125.2, 122.4, 115.1, 108.1, 102.6, 68.6, 67.7,64.8, 47.7, 20.9; IR (KBr) υ: 3230, 2872, 2221, 1744, 1656, 1603, 1545, 1498, 1458, 1383, 1155, 1099, 979, 873, 772, 738, 700 cm^{-1}; MS (m/z): HRMS (ESI) Calcd. for $C_{33}H_{22}N_7O$ ($[M-H]^-$): 532.1891. Found: 532.1891.

5-butyl-15-cyano-16-oxo-14b,15-dihydro-5a,15-(epiminomethano)indolo[2'',3'':2',3']pyrrolo[3',4':4,5]pyrrolo[2,1-a]isoquinolin-7(5H,6H,7aH)-ylidene)malononitrile (2h): white solid, 55%, m.p. 236-238℃; ^{1}H NMR (400 MHz, DMSO) δ: 11.91 (s, 1H, NH), 10.63 (s, 1H, NH), 7.36-7.32 (m 1H, ArH), 7.28-7.16 (m, 5H, ArH), 6.82-6.78 (m, 1H, ArH), 6.59-6.57 (m, 2H, ArH), 5.72 (s, 1H, CH), 5.66 (d, J = 7.2Hz, 2H, CH), 5.29 (s, 1H, CH), 3.45-3.41 (m, 2H, CH), 1.61-1.49 (m, 2H, CH), 1.36-1.28 (m, 2H, CH), 0.94 (t, J = 7.2Hz, 3H, CH_3); ^{13}C NMR (150 MHz, DMSO) δ: 171.3, 167.6, 148.3, 134.4, 131.5, 130.8, 129.4, 128.0, 125.9, 125.1, 124.7, 121.6, 118.3, 114.6, 114.6, 107.0, 102.1, 101.6, 79.4, 68.5, 67.0, 64.1, 42.6, 39.9, 39.8, 29.2, 19.7, 13.7; IR (KBr) υ: 3627, 3169, 2959, 2870, 2219, 1715, 1607, 1491, 1460, 1415, 1369, 1324, 1275, 1164, 1086, 948, 918, 774, 743 cm^{-1}; MS (m/z): HRMS (ESI) Calcd. for $C_{29}H_{22}N_7O$ ($[M-H]^-$): 484.1891. Found: 484.1903.

5-butyl-2-chloro-15-cyano-16-oxo-14b,15-dihydro-5a,15-(epiminomethano)indolo[2'',3'':2',3']pyrrolo[3',4':4,5]pyrrolo[2,1-a]isoquinolin-7(5H,6H,7aH)-ylidene)malononitrile (2i): white solid, 56%, m.p. 288-290℃; ^{1}H NMR (400 MHz, DMSO) δ: 11.96 (s, 1H, NH), 10.70 (s, 1H, NH), 7.36-7.30 (m, 2H, ArH), 7.24-7.17 (m, 3H, ArH), 7.09 (m, 1H, ArH), 6.62-6.60 (m, 2H, ArH), 5.84 (s, 1H, CH), 5.71 (d, J = 7.6Hz, 1H, CH), 5.32 (s, 1H, CH), 3.48-3.42 (m, 2H, CH), 1.59-1.51 (m, 2H, CH), 1.35-1.30 (m, 2H, CH), 0.94 (t, J = 7.2Hz, 3H, CH_3); ^{13}C NMR (150 MHz, DMSO) δ: 171.1, 167.3, 147.3, 134.2, 131.4, 130.5, 129.4, 128.0, 125.9, 125.0, 124.8, 123.4, 121.4, 114.5, 114.4, 108.3, 102.3, 101.8, 79.0, 68.5, 66.6, 64.0, 47.7, 42.6, 29.0, 19.6, 13.7; IR (KBr) υ: 3226, 2929, 2221, 1721, 1655, 1615, 1564, 1544, 1493, 1460, 1422, 1368, 1272, 1169, 1096, 961, 871, 816, 771, 741 cm^{-1}; MS (m/z): HRMS (ESI) Calcd. for $C_{29}H_{21}ClN_7O$ ($[M-H]^-$): 518.1502. Found: 518.1507.

5-butyl-15-cyano-2-methyl-16-oxo-14b,15-dihydro-5a,15-(epiminomethano)indolo[2'',3'':2',3']pyrrolo[3',4':4,5]pyrrolo[2,1-a]isoquinolin-7(5H,6H,7aH)-ylidene)malononitrile (2j): white solid, 55%, m.p. 252-254℃; ^{1}H NMR (600 MHz, DMSO) δ: 11.84 (s, 1H, NH), 10.55 (s, 1H, NH), 7.34 (brs, 1H, ArH), 7.23-7.17 (m, 3H, ArH), 7.07 (brs, 1H, ArH), 6.99 (s, 1H, ArH), 6.58 (brs, 1H, ArH), 6.48 (brs, 1H, CH), 5.69-5.68 (m, 2H, CH), 5.26 (s, 1H, CH), 3.40 (m, 2H, CH), 2.21 (s, 3H, CH_3), 1.57 (m, 2H, CH), 1.33 (m, 2H, CH), 0.94 (m, 3H, CH_3); ^{13}C NMR (150 MHz,

DMSO) δ: 171.2, 167.6, 146.3, 134.4, 131.5, 129.3, 128.0, 126.9, 125.8, 125.7, 125.1, 124.7, 121.7, 114.6, 108.7, 107.5, 106.9, 102.0, 79.2, 79.1, 70.8, 68.5, 66.9, 54.1, 42.8, 29.2, 20.4, 19.7, 13.8; IR (KBr) υ: 3458, 3233, 2956, 2867, 2215, 1715, 1610, 1497, 1456, 1374, 1279, 1161, 1095, 961, 916, 875, 809, 772, 738 cm^{-1}; MS (m/z): HRMS (ESI) Calcd. for $C_{30}H_{24}N_7O$ ($[M-H]^-$): 498.2048. Found: 498.2052.

15-cyano-5-methyl-16-oxo-14b,15-dihydro-5a,15-(epiminomethano)indolo[2'',3'':2',3']pyrrolo[3',4':4,5]pyrrolo[2,1-a]isoquinolin-7(5H,6H,7aH)-ylidene)malononitrile (2k): white solid, 62%, m.p. 254-256℃; ^1H NMR (400 MHz, DMSO) δ: 11.90 (s, 1H, NH), 10.70 (s, 1H, NH), 7.36-7.17 (m, 6H, ArH), 6.86-6.83 (m, 1H, ArH), 6.64 (d, J = 7.6Hz, 1H, ArH), 6.59 (d, J = 7.2Hz, 1H, CH), 5.72 (s, 1H, CH), 5.67 (d, J = 7.2Hz, 1H, CH), 5.33 (s, 1H, CH), 2.98 (s, 3H, CH_3); ^{13}C NMR (100 MHz, DMSO) δ: 172.0, 168.2, 150.1, 134.8, 132.0, 131.2, 129.8, 128.5, 126.3, 125.5, 125.2, 122.3, 119.4, 115.1, 107.8, 102.5, 79.5, 79.4, 68.6, 67.4, 64.7, 60.2, 29.7; IR (KBr) υ: 3238, 2928, 2216, 1721, 1656, 1544, 1494, 1459, 1385, 1223, 1122, 999, 944, 867, 763 cm^{-1}; MS (m/z): HRMS (ESI) Calcd. for $C_{26}H_{16}N_7O$ ($[M-H]^-$): 442.1422. Found: 442.1417.

2-chloro-15-cyano-5-(4-methylbenzyl)-16-oxo-14b,15-dihydro-5a,15-(epiminomethano)indolo[2'',3'':2',3']pyrrolo[3',4':4,5]pyrrolo[2,1-a]isoquinolin-7(5H,6H,7aH)-ylidene)malononitrile (2l): white solid, 66%, m.p. 278-280℃; ^1H NMR (400 MHz, DMSO) δ: 12.01 (s, 1H, NH), 10.84 (s, 1H, NH), 7.36 (td, J_1 = 7.2 Hz, J_2 = 2.0 Hz, 1H, ArH), 7.26-7.12 (m, 9H, ArH), 6.63 (d, J = 7.6 Hz, 1H, ArH), 6.02 (d, J = 8.4Hz, 1H, CH), 5.96 (s, 1H, CH), 5.73 (d, J = 7.6 Hz, 1H, CH), 5.39 (s, 1H, CH), 4.85 (d, J = 16.8 Hz, 1H, CH), 4.52 (d, J = 16.8 Hz, 1H, CH), 2.29 (s, 3H, CH_3); ^{13}C NMR (150 MHz, DMSO) δ: 171.4, 167.2, 147.3, 136.2, 134.2, 133.8, 131.5, 130.3, 129.4, 129.1, 128.1, 126.5, 126.0, 125.1, 124.8, 123.7, 122.2, 114.5, 109.0, 102.4, 102.1, 78.8, 68.3, 66.9, 64.2, 46.9, 20.6; IR (KBr) υ: 3454, 3070, 2860, 2231, 1721, 1620, 1564, 1544, 1482, 1383, 1101, 973, 773 cm^{-1}; MS (m/z): HRMS (ESI) Calcd. for $C_{33}H_{21}ClN_7O$ ($[M-H]^-$): 566.1502. Found: 566.1511.

(6'-amino-1-benzyl-5'-cyano-3'-(isoquinolin-2-ium-2-yl)-5-methyl-2-oxo-3'H-spiro[indoline-3,4'-pyridin]-2'-yl)(cyano)(isocyano)methanide (3a): white solid, 44%, m.p. 254-256℃; ^1H NMR (400 MHz, DMSO) δ: 9.99 (m, 2H, NH), 8.72-8.70 (m, 1H, ArH), 8.49-8.33 (m, 3H, ArH), 8.14-8.10 (m, 1H, ArH), 7.38-7.24 (m, 5H, ArH), 7.15-7.13 (m, 1H, ArH), 6.86-6.82 (m, 2H, CH), 6.74 (s, 2H, CH), 5.83 (s, 1H, CH), 4.95 (d, J = 15.8 Hz, 1H, CH), 4.54 (d, J = 15.8 Hz, 1H, CH), 2.29 (s, 3H, CH_3); ^{13}C NMR (150 MHz, DMSO) δ: 172.2, 161.0, 138.0, 137.6, 135.5, 132.6, 131.7, 131.0, 129.4, 128.6, 128.4, 128.2, 128.0, 127.4, 127.3, 127.2, 127.0, 126.9, 122.8, 121.5, 120.6, 118.9, 117.4, 109.7, 62.8, 51.3, 51.3, 50.6, 43.5, 43.2, 20.8; IR (KBr) υ: 3424, 3314, 3208, 3071, 2921, 2206, 2175, 1702, 1635, 1551, 1503, 1433, 1365, 1283, 1189, 1113, 1024, 930, 868, 811, 753, 704 cm^{-1}; MS (m/z): HRMS (ESI) Calcd. for $C_{33}H_{24}N_7O$ ($[M-H]^-$): 534.2037. Found: 534.2030.

6'-amino-1-butyl-5'-cyano-3'-(isoquinolin-2-ium-2-yl)-5-methyl-2-oxo-3'H-spiro[indoline-3,4'-pyridin]-2'-yl)(cyano)(isocyano)methanide (3b): white solid, 43%, m.p. 265-267℃; ^{1}H NMR (400 MHz, DMSO) δ: 9.92 (m, 2H, NH), 8.68 (d, *J* = 6.8 Hz, 1H, ArH), 8.45-8.39 (m, 2H, ArH), 8.36-8.32 (m, 1H, ArH), 8.11 (t, *J* = 7.2 Hz, 1H, ArH), 7.23 (d, *J* = 7.6 Hz, 1H, ArH), 7.10 (d, *J* = 8.0 Hz, 1H, ArH), 6.84 (s, 1H, CH), 6.64 (s, 2H, CH), 5.73 (s, 1H, CH), 3.61-3.54 (m, 1H, CH), 3.48-3.41 (m, 1H, CH), 2.32 (s, 3H, CH_3), 1.52-1.42 (m, 2H, CH), 1.28-1.21 (m, 2H, CH), 0.80 (t, *J* = 7.2 Hz, 3H, CH_3); ^{13}C NMR (150 MHz, DMSO) δ: 171.7, 162.3, 139.7, 138.0, 137.5, 132.2, 131.9, 131.6, 130.9, 129.5, 127.4, 122.8, 121.6, 120.3, 118.9, 117.4, 109.3, 62.8, 59.7, 51.5, 51.1, 50.3, 28.8, 20.8, 19.3, 19.3, 13.6, 13.5; IR (KBr) υ: 3459, 3332, 3216, 3078, 2961, 2868, 2207, 2173, 1705, 1632, 1555, 1507, 1438, 1363, 1288, 1199, 1152, 1109, 1029, 925, 879, 819, 757 cm^{-1}; MS (m/z): HRMS (ESI) Calcd. for $C_{30}H_{26}N_7O$ ($[M-H]^-$): 500.2193. Found: 500.2187.

Ethyl 10'-amino-1-benzyl-12'-cyano-2-oxo-11'H-spiro[indoline-3,8'-pyrido[2',3':4,5]pyrrolo-[2,1-a]isoquinoline]-9'-carboxylate (4a): white solid, 71%, m.p. 216-218℃; ^{1}H NMR (400 MHz, DMSO) δ: 9.77 (s, 1H, NH), 8.54 (d, *J* = 8.0 Hz, 1H, ArH), 7.72-7.65 (m, 2H, ArH), 7.60-7.54 (m, 3H, ArH), 7.45-7.38 (m, 3H, ArH), 7.33-7.27 (m, 2H, ArH), 7.03 (d, *J* = 7.2 Hz, 1H, ArH), 6.95 (t, *J* = 7.2 Hz, 1H, ArH), 6.69 (t, *J* = 7.6 Hz, 1H, CH), 6.56 (d, *J* = 7.6 Hz, 1H, CH), 5.16 (d, *J* = 14.8 Hz, 1H, CH), 4.86 (d, *J* = 14.8 Hz, 1H, CH), 3.74-3.68 (m, 1H, CH), 3.48-3.40 (m, 1H, CH), 0.60 (t, *J* = 7. 2Hz, 3H, CH_3); ^{13}C NMR (150 MHz, DMSO) δ: 177.2, 167.8, 153.9, 142.8, 136.5, 133.1, 131.2, 128.9, 128.7, 128.5, 128.3, 128.2, 127.9, 127.5, 126.9, 123.9, 123.1, 122.9, 121.2, 120.9, 116.1, 112.9, 108.5, 107.1, 75.7, 71.2, 57.8, 50.2, 44.0, 13.6; IR (KBr) υ: 3490, 3372, 2927, 2209, 1657, 1624, 1509, 1462, 1372, 1295, 1187, 1105, 782, 700 cm^{-1}; MS (m/z): HRMS (ESI) Calcd. for $C_{33}H_{24}N_5O_3$ ($[M-H]^-$): 538.1885. Found: 538.1869.

Ethyl 10'-amino-1-benzyl-12'-cyano-5-fluoro-2-oxo-11'H-spiro[indoline-3,8'-pyrido-[2',3':4,5]pyrrolo[2,1-a]isoquinoline]-9'-carboxylate (4b): white solid, 72%, m.p. 290-292℃; ^{1}H NMR (400 MHz, DMSO) δ: 9.79 (s, 1H, NH), 8.55 (d, *J* = 8.4 Hz, 1H, ArH), 7.74-7.67 (m, 2H, ArH), 7.60-7.56 (m, 3H, ArH), 7.45-7.36 (m, 3H, ArH), 7.34-7.31 (m, 1H, ArH), 7.14 (td, J_1 = 9.2 Hz, J_2 = 1.2 Hz, 1H, ArH), 6.99-6.96 (m, 1H, ArH), 6.67 (t, *J* = 7.2 Hz, 1H, CH), 6.60 (d, *J* = 7.2 Hz, 1H, CH), 5.15 (d, *J* = 14.8 Hz, 1H, CH), 4.87 (d, *J* = 14.8 Hz, 1H, CH), 3.79-3.71 (m, 1H, CH), 3.49-3.41 (m, 1H, CH), 0.62 (t, *J* = 7.2 Hz, 3H, CH_3); ^{13}C NMR (150 MHz, DMSO) δ: 177.1, 167.7, 159.9, 158.3, 153.9, 139.0, 136.3, 135.1, 135.0, 131.4, 128.9, 128.7, 128.6, 128.5, 128.2, 127.9, 127.5, 126.9, 122.9, 121.3, 120.8, 116.1, 114.6, 114.4, 113.1, 111.9, 111.8, 109.4, 109.3, 106.5, 75.4, 71.3, 57.9, 50.6, 44.1, 13.7; IR (KBr) υ: 3474, 3364, 3169, 2976, 2208, 1658, 1624, 1515, 1369, 1288, 1169, 1107, 1068, 782 cm^{-1}; MS (m/z): HRMS (ESI) Calcd. for $C_{33}H_{23}FN_5O_3$ ($[M-H]^-$): 556.1790. Found: 556.1799.

Ethyl 10'-amino-1-benzyl-5-chloro-12'-cyano-2-oxo-11'H-spiro[indoline-3,8'-pyrido-[2',3':4,5]pyrrolo[2,1-a]isoquinoline]-9'-carboxylate (4c): white solid, 75%, m.p. 308-310℃; ^{1}H NMR (600 MHz, DMSO) δ: 9.79 (s, 1H, NH), 8.55 (d, J = 7.8 Hz, 1H, ArH), 7.73-7.67 (m, 2H, ArH), 7.58 (m, 3H, ArH), 7.43 (m, 2H, ArH), 7.40-7.39 (m, 1H, ArH), 7.36 (m, 2H, ArH), 7.10 (brs, 1H, ArH), 6.66-6.65 (m, 1H, CH), 6.59-6.58 (m, 1H, CH), 5.15 (d, J = 15.0 Hz, 1H, CH), 4.89 (d, J = 15.0 Hz, 1H, CH), 3.77-3.76 (m, 1H, CH), 3.49-3.48 (m, 1H, CH), 0.64 (m, 3H, CH_3); ^{13}C NMR (150 MHz, DMSO) δ: 177.4, 168.0, 154.4, 142.1, 136.6, 135.6, 131.8, 129.7,129.6, 129.2, 129.1, 129.0, 128.9, 128.3, 127.7, 123.3, 121.8, 121.6, 121.4, 120.9, 116.5, 113.8, 113.4, 110.9, 110.1, 106.7, 75.6, 71.8, 50.8, 14.2, 14.1; IR (KBr) υ: 3383, 3275, 2933, 2219, 1713, 1662, 1505, 1479, 1373, 1323, 1292, 1162, 1107, 1081, 1048, 874, 830, 783, 741, 704 cm^{-1}; MS (m/z): HRMS (ESI) Calcd. for $C_{33}H_{23}ClN_5O_3$ ([M-H]$^-$): 572.1495. Found: 572.1477.

Ethyl 10'-amino-1-butyl-5-chloro-12'-cyano-2-oxo-11'H-spiro[indoline-3,8'-pyrido-[2',3':4,5]pyrrolo[2,1-a]isoquinoline]-9'-carboxylate (4d): white solid, 68%, m.p. 306-308℃; ^{1}H NMR (400 MHz, DMSO) δ: 9.81 (s, 1H, NH), 8.57 (d, J = 8.4 Hz, 1H, ArH), 7.79 (d, J = 8.0 Hz, 1H, ArH), 7.73-7.69 (m, 1H, ArH), 7.62-7.58 (m, 1H, ArH), 7.41-7.38 (m, 1H, ArH), 7.24 (d, J = 8.0 Hz, 1H, ArH), 7.13-7.11 (m, 2H, ArH), 6.96 (m, 1H, ArH), 3.95-3.88 (m, 1H, CH), 3.80-3.75 (m, 1H, CH), 3.70-3.60 (m, 2H, CH), 1.76-1.64 (m, 2H, CH), 1.49-1.40 (m, 2H, CH), 0.98 (d, J = 7.2 Hz, 3H, CH_3), 0.71 (d, J = 7.2 Hz, 3H, CH_3); ^{13}C NMR (150 MHz, DMSO) δ: 176.7, 167.6, 153.9, 142.0, 135.2, 131.4, 128.7, 128.5, 128.3, 128.2, 127.5, 127.0, 123.9, 123.0, 121.3, 120.6, 116.1, 113.4, 109.7, 106.4, 75.3, 71.4, 57.9, 50.3, 29.4, 20.6, 13.8, 13.6; IR (KBr) υ: 3436, 3269, 3096, 2960, 2869, 2212, 1661, 1621, 1564, 1513, 1480, 1429, 1370, 1297, 1202, 1167, 1111, 1072, 878, 814, 778 cm^{-1}; MS (m/z): HRMS (ESI) Calcd. for $C_{30}H_{25}ClN_5O_3$ ([M-H]$^-$): 538.1651. Found: 538.1648.

Ethyl 10'-amino-1-butyl-12'-cyano-5-methyl-2-oxo-11'H-spiro[indoline-3,8'-pyrido-[2',3':4,5]pyrrolo[2,1-a]isoquinoline]-9'-carboxylate (4e): white solid, 66%, m.p. 298-300℃; ^{1}H NMR (400 MHz, DMSO) δ: 9.73 (s, 1H, NH), 8.56 (d, J = 8.0 Hz, 1H, ArH), 7.78 (d, J = 7.6 Hz, 1H, ArH), 7.72-7.68 (m, 1H, ArH), 7.60-7.57 (m, 1H, ArH), 7.12-7.07 (m, 3H, ArH), 7.02-7.00 (m, 1H, ArH), 6.87 (s, 1H, ArH), 3.93-3.87 (m, 1H, CH), 3.75-3.69 (m, 1H, CH), 3.66-3.58 (m, 2H, CH), 2.15 (s, 3H, CH_3), 1.79-1.62 (m, 2H, CH), 1.48-1.42 (m, 2H, CH), 0.98 (d, J = 6.8 Hz, 3H, CH_3), 0.71 (d, J = 6.8 Hz, 3H, CH_3); ^{13}C NMR (150 MHz, DMSO) δ: 176.8, 167.9, 153.8, 140.7, 133.1, 131.9, 131.2, 128.6, 128.4, 128.3, 128.1, 127.4, 126.9, 124.4, 122.9, 121.2, 120.9, 116.1, 113.1, 107.8, 107.4, 75.8, 71.2, 57.8, 50.2, 29.5, 20.4, 13.7, 13.6; IR (KBr) υ: 3432, 3272, 2959, 2868, 2211, 1668, 1624, 1564, 1513, 1459, 1370, 1296, 1197, 1111, 883, 780 cm^{-1}; MS (m/z): HRMS (ESI) Calcd. for $C_{31}H_{28}N_5O_3$ ([M-H]$^-$): 518.2198. Found: 518.2202.

Methyl 10'-amino-1-benzyl-5-chloro-12'-cyano-2-oxo-11'H-spiro[indoline-3,8'-pyrido-[2',3':4,5]pyrrolo[2,1-a]isoquinoline]-9'-carboxylate (4f): white solid, 66%, m.p. 298-300℃; ^{1}H NMR (400 MHz, DMSO) δ: 9.83 (s, 1H, NH), 8.56 (d, *J* = 8.4Hz, 1H, ArH), 7.77 (d, *J* = 7.6Hz, 1H, ArH), 7.70 (t, *J* = 7.6Hz, 1H, ArH), 7.61-7.58 (m, 3H, ArH), 7.45 (t, *J* = 7.2Hz, 2H, ArH), 7.40-7.30 (m, 3H, ArH), 7.10 (m, 1H, ArH), 6.81-6.75 (m, 2H, ArH), 5.10 (d, *J* = 14.8Hz, 1H, CH), 4.99 (d, *J* = 14.8Hz, 1H, CH), 2.98 (s, 3H, CH_3); ^{13}C NMR (150 MHz, DMSO) δ: 177.0, 167.8, 153.7, 141.4, 136.2, 135.0, 131.5, 128.8, 128.6, 128.3, 127.9, 127.6, 127.2, 126.9, 123.9, 122.9, 121.3, 120.6, 116.1, 113.4, 110.1, 106.3, 75.4, 71.4, 50.3, 49.6, 44.1; IR (KBr) υ: 3390, 3285, 3139, 2946, 2218, 1715, 1667, 1621, 1506, 1480, 1432, 1373, 1298, 1191, 1160, 1114, 1078, 871, 831, 780, 743, 702 cm^{-1}; MS (m/z): HRMS (ESI) Calcd. for $C_{32}H_{21}ClN_5O_3$ ($[M-H]^-$): 558.1338. Found: 558.1343.

Methyl 10'-amino-1-benzyl-12'-cyano-5-methyl-2-oxo-11'H-spiro[indoline-3,8'-pyrido-[2',3':4,5]pyrrolo[2,1-a]isoquinoline]-9'-carboxylate (4g): white solid, 67%, m.p. 282-284℃; ^{1}H NMR (400 MHz, DMSO) δ: 9.76 (s, 1H, NH), 8.55 (d, *J* = 8.4 Hz, 1H, ArH), 7.75 (d, *J* = 7.6 Hz, 1H, ArH), 7.69 (t, *J* = 7.6 Hz, 1H, ArH), 7.59-7.56 (m, 3H, ArH), 7.44 (t, *J* = 7.2 Hz, 2H, ArH), 7.39-7.35 (m, 1H, ArH), 7.15 (d, *J* = 8.0 Hz, 1H, ArH), 7.06 (d, *J* = 8.0 Hz, 1H, ArH), 6.83 (s, 1H, ArH), 6.80 (d, *J* = 7.2 Hz, 1H, CH), 6.75 (d, *J* = 7.2 Hz, 1H, CH), 5.08 (d, *J* = 14.8 Hz, 1H, CH), 4.92 (d, *J* = 14.8 Hz, 1H, CH), 2.97 (s, 3H, CH_3), 2.12 (s, 3H, CH_3); ^{13}C NMR (150 MHz, DMSO) δ: 177.2, 168.1, 153.6, 140.2, 136.6, 132.9, 132.1, 131.2, 128.8, 128.7, 128.6, 128.5, 128.4, 128.2, 127.8, 127.5, 126.9, 124.3, 122.9, 121.2, 121.0, 116.1, 113.2, 108.3, 107.3, 75.9, 71.2, 50.2, 49.6, 44.0, 20.4; IR (KBr) υ: 3451, 3299, 2945, 2210, 1660, 1624, 1567, 1504, 1459, 1434, 1372, 1309, 1187, 1114, 1078, 874, 784 cm^{-1}; MS (m/z): HRMS (ESI) Calcd. for $C_{33}H_{24}N_5O_3$ ($[M-H]^-$): 538.1885. Found: 538.1894.

1-butyl-5-chloro-1',1'-diisocyano-3'-(4-nitrophenyl)-1',10b'-dihydro-3'H-spiro[indoline-3,2'-pyrrolo[2,1-a]isoquinolin]-2-one (1a):

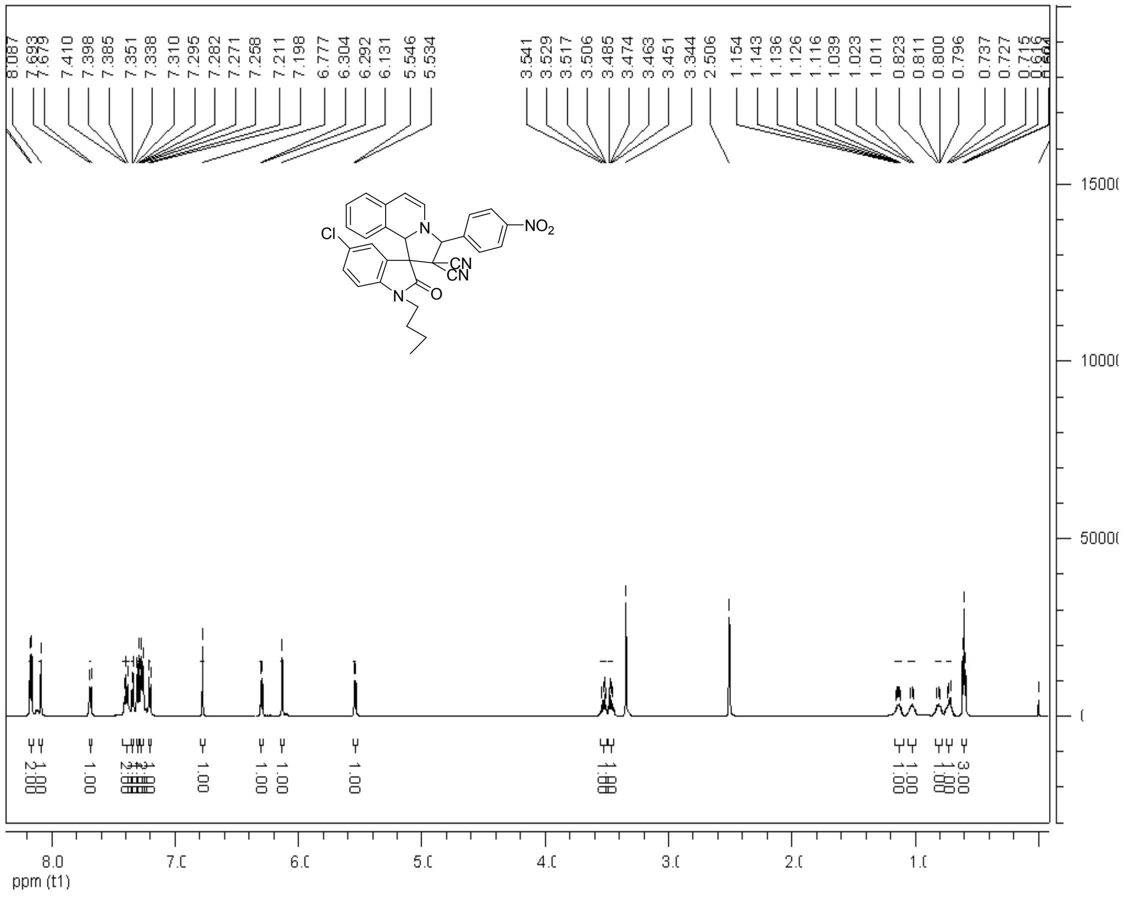

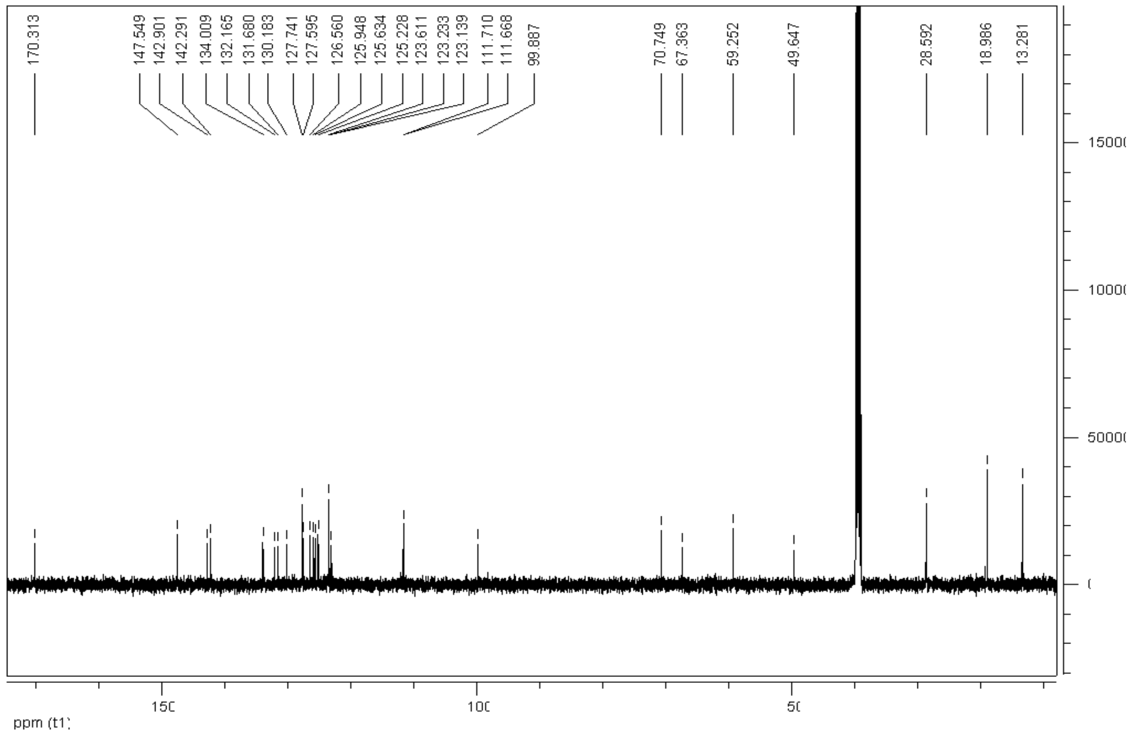

1-benzyl-5-chloro-1',1'-diisocyano-3'-(4-nitrophenyl)-1',10b'-dihydro-3'H-spiro[indoline-3,2'-pyrrolo[2,1-a]isoquinolin]-2-one (1b):

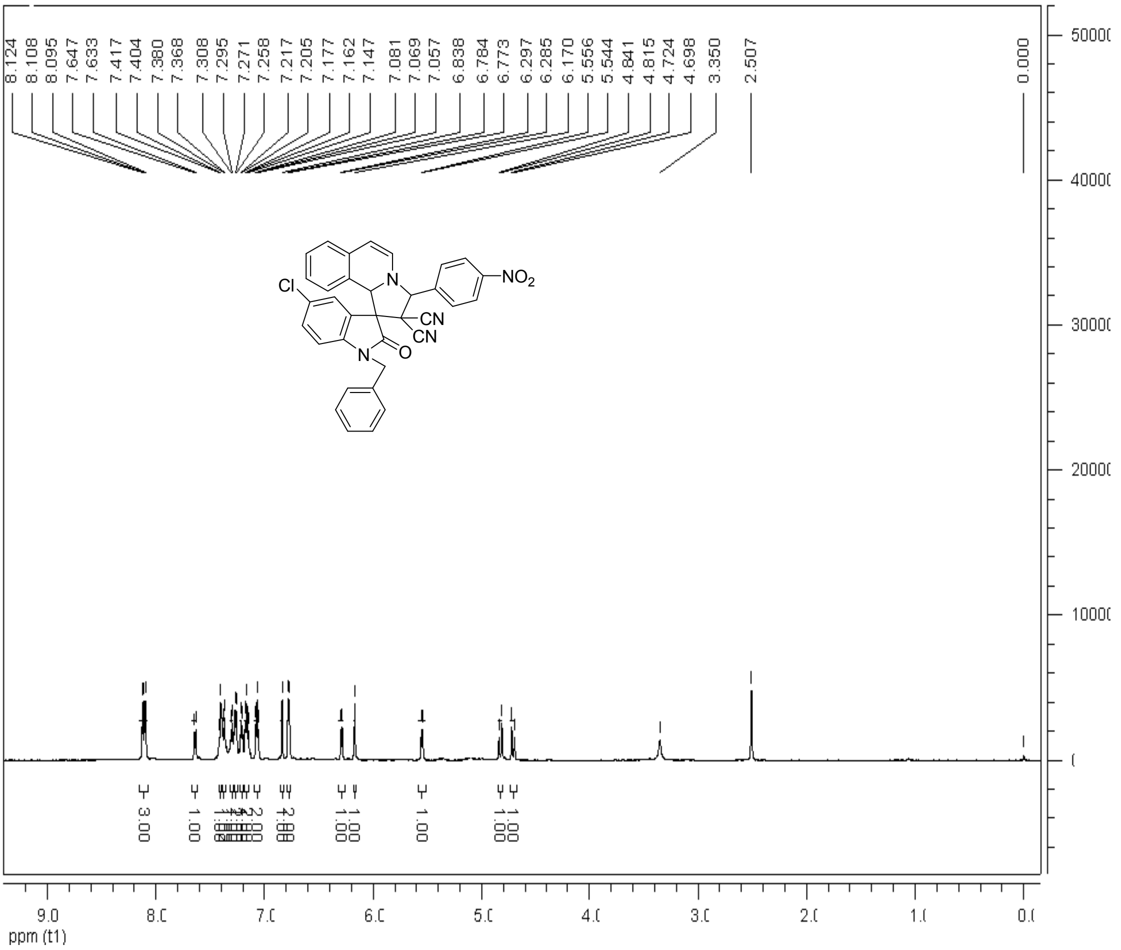

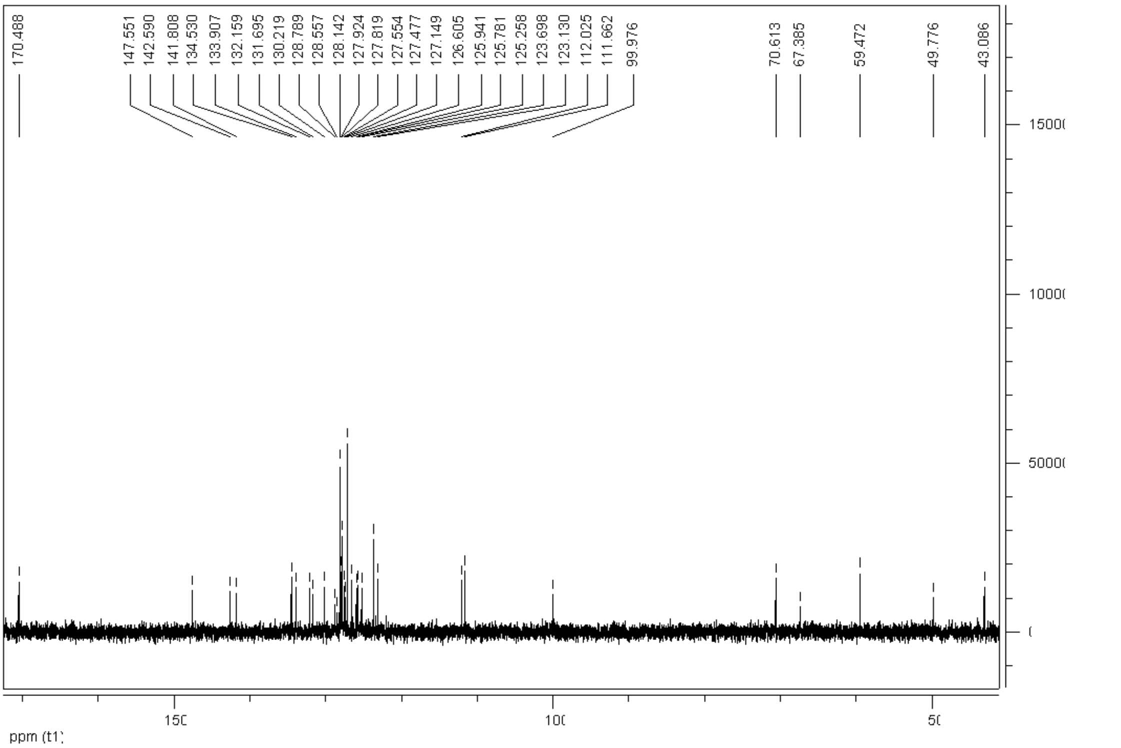

1-Butyl-5-methyl-1',1'-diisocyano-3'-(4-nitrophenyl)-1',10b'-dihydro-3'H-spiro[indoline-3,2'-pyrrolo[2,1-a]isoquinolin]-2-one (1c):

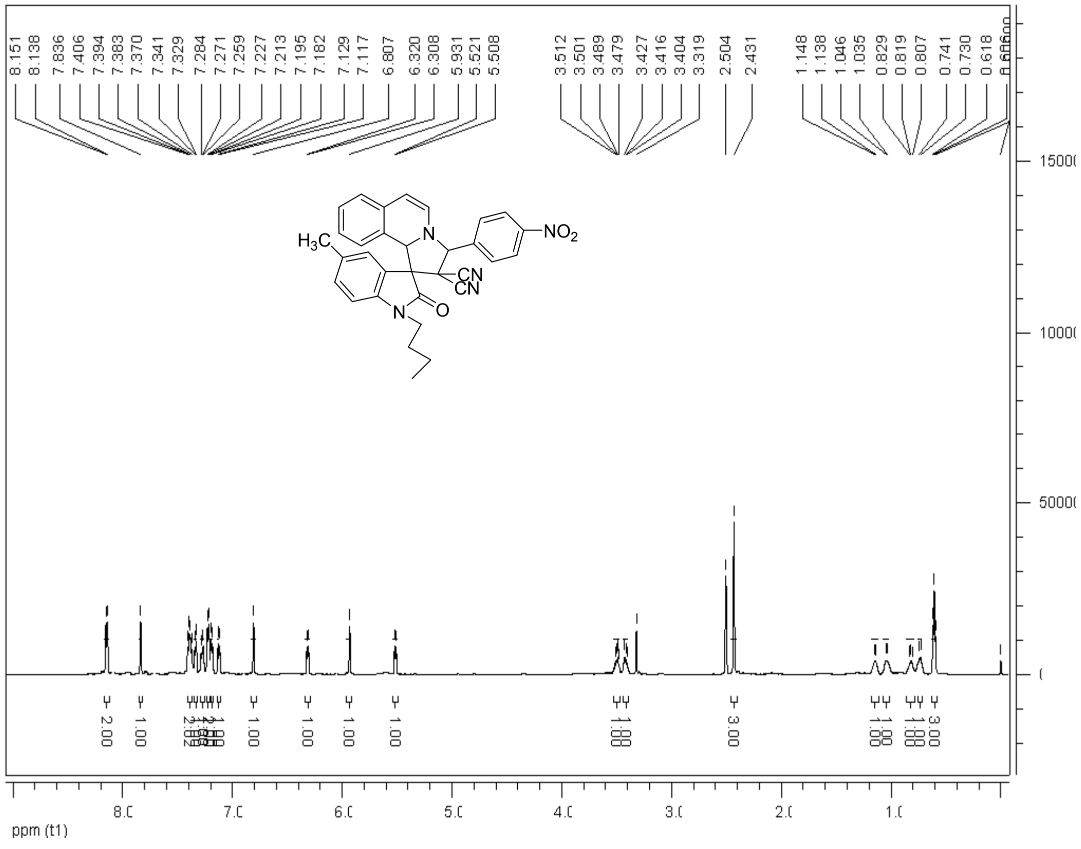

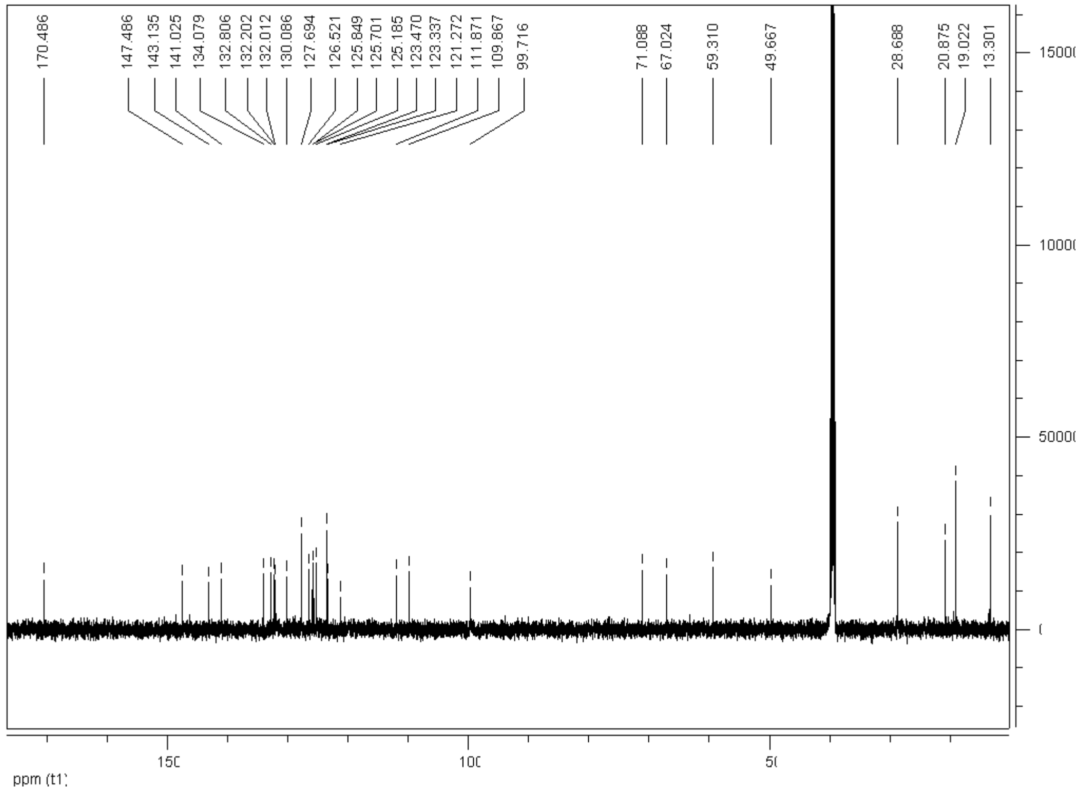

5-methyl-1',1'-diisocyano-3'-(4-nitrophenyl)-1',10b'-dihydro-3'H-spiro[indoline-3,2'-pyrrolo[2,1-a]isoquinolin]-2-one (1d):

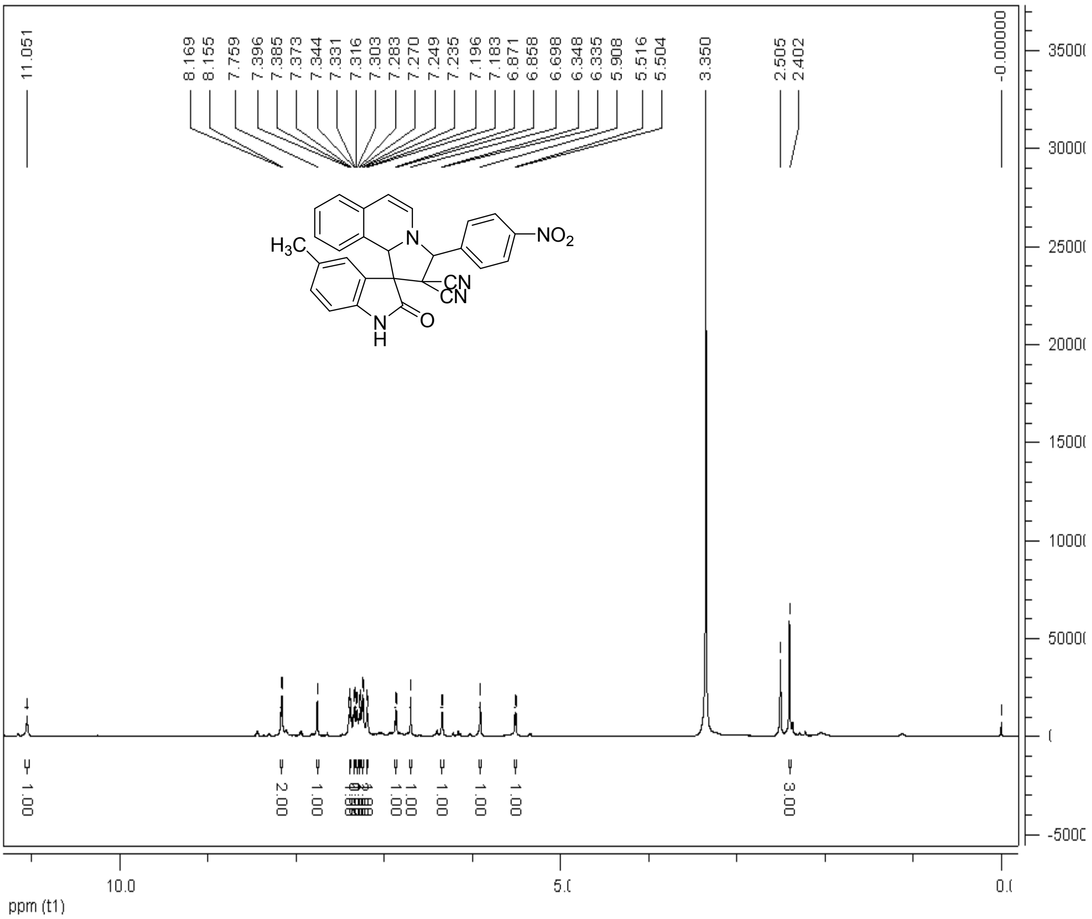

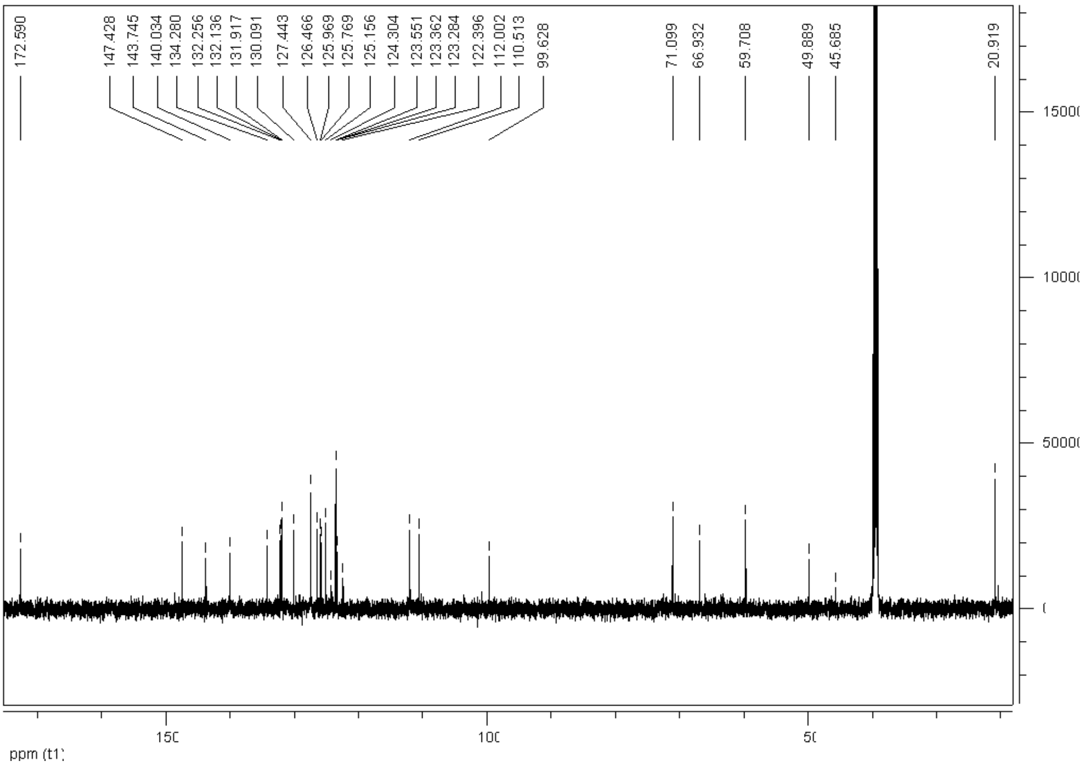

1-benzyl-5-methyl-1',1'-diisocyano-3'-(4-nitrophenyl)-1',10b'-dihydro-3'H-spiro[indoline-3,2'-pyrrolo[2,1-a]isoquinolin]-2-one (1e):

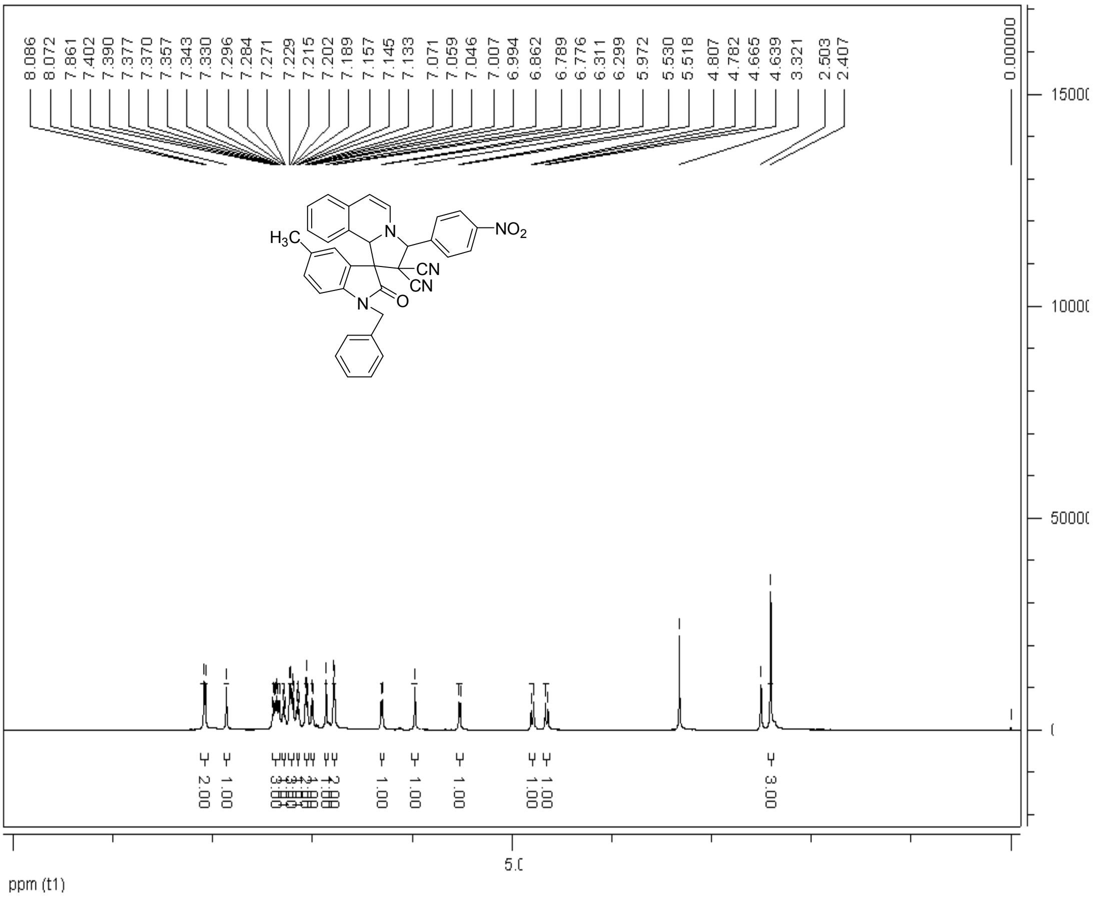

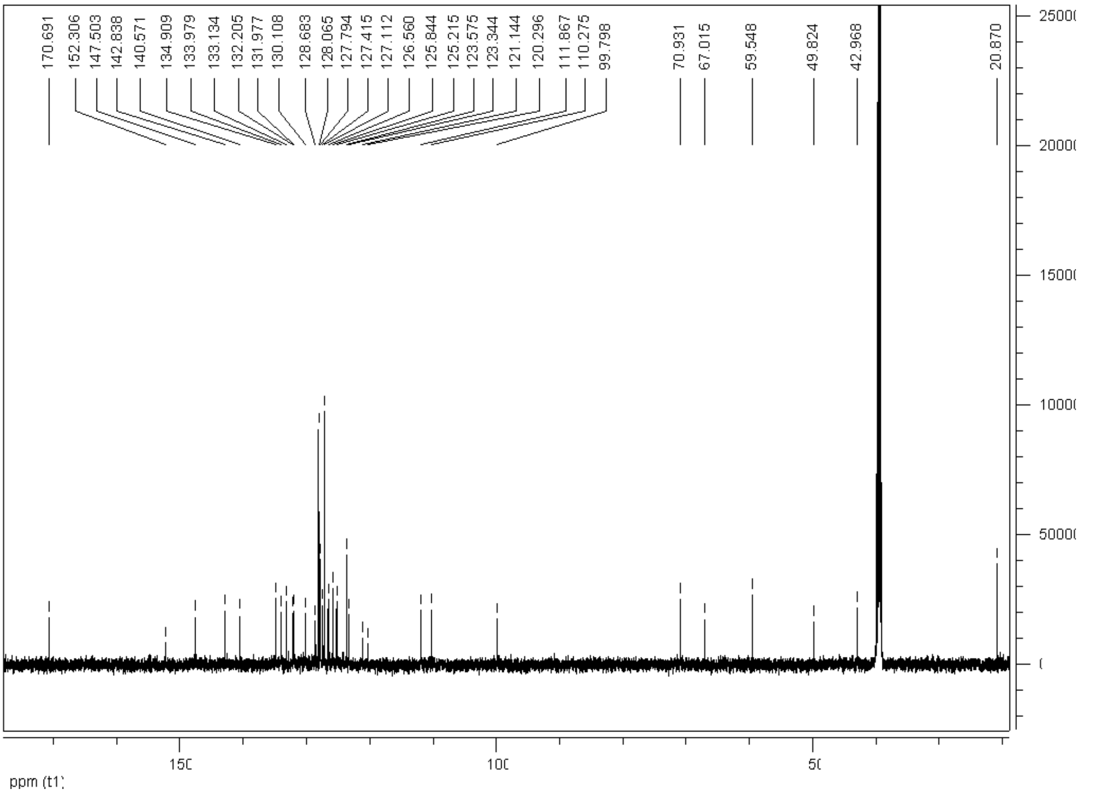

1-benzyl-1',1'-diisocyano-3'-(4-nitrophenyl)-1',10b'-dihydro-3'H-spiro[indoline-3,2'-pyrrolo[2,1-a]isoquinolin]-2-one (1f):

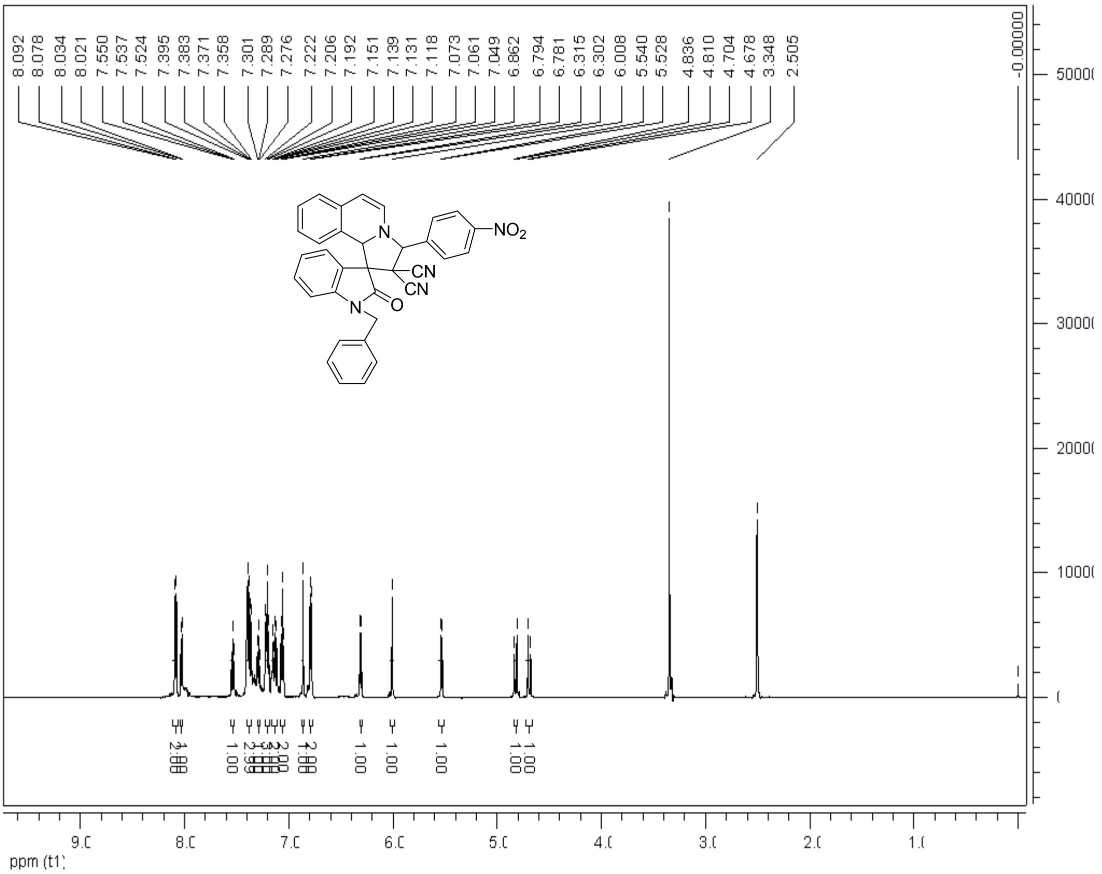

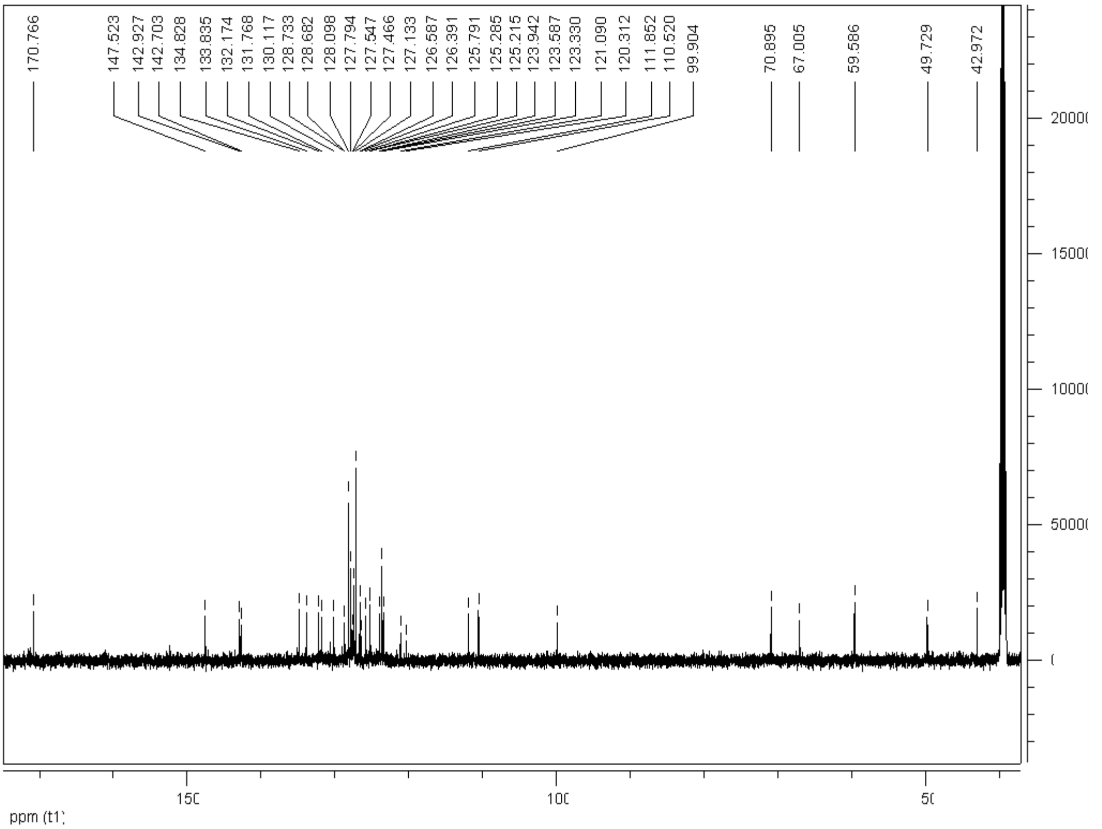

1-butyl-5-fluoro-1',1'-diisocyano-3'-(4-nitrophenyl)-1',10b'-dihydro-3'H-spiro[indoline-3,2'-pyrrolo[2,1-a]isoquinolin]-2-one (1g):

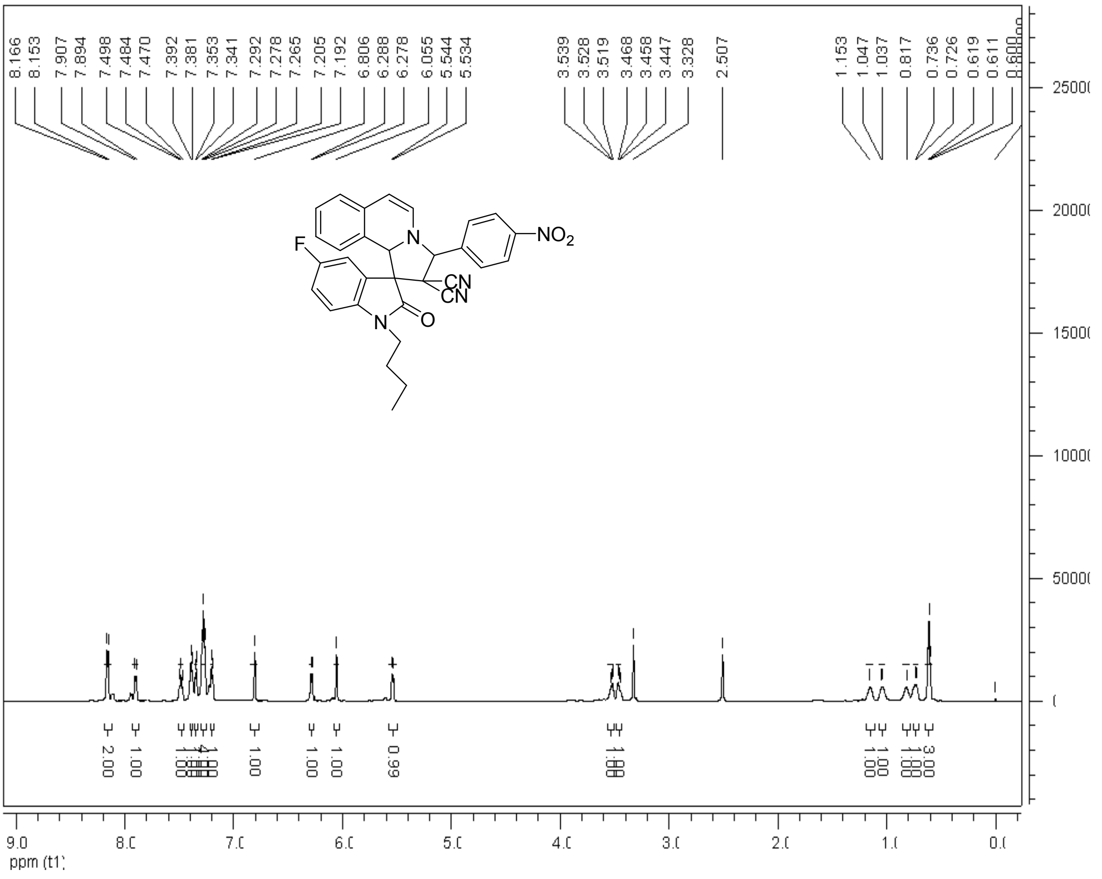

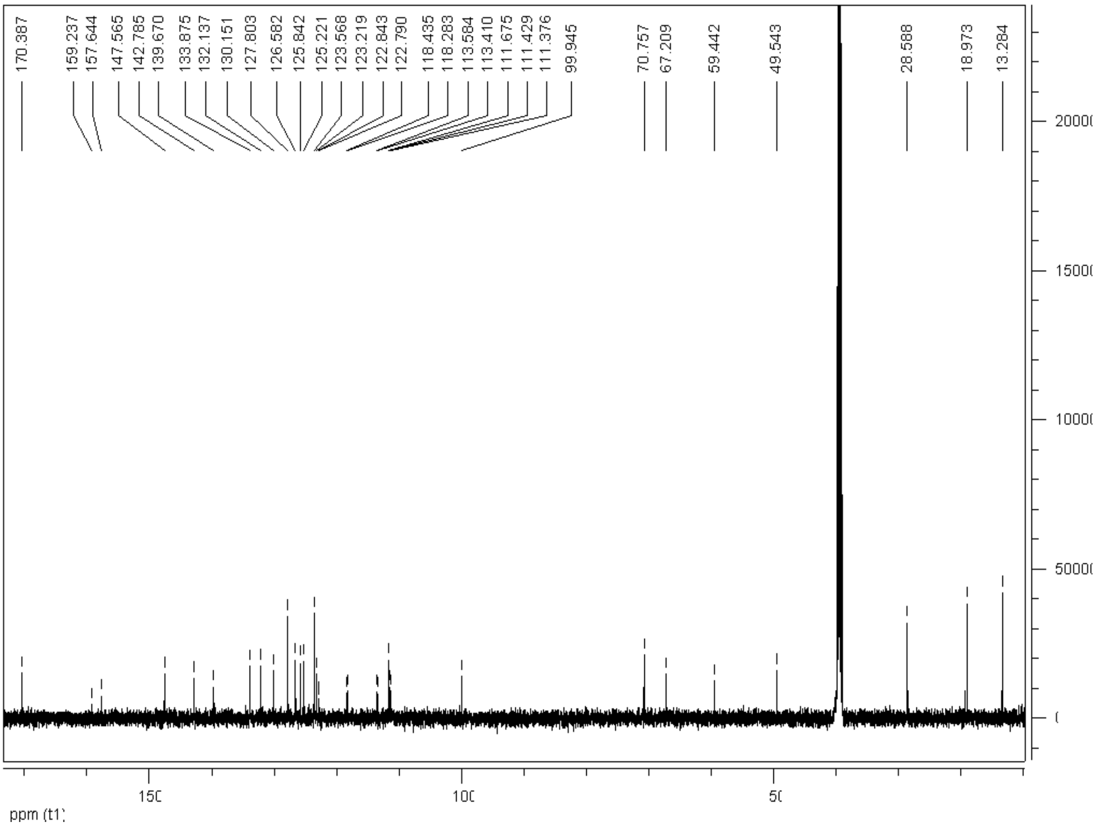

1-benzyl-5-fluoro-1',1'-diisocyano-3'-(4-nitrophenyl)-1',10b'-dihydro-3'H-spiro[indoline-3,2'-pyrrolo[2,1-a]isoquinolin]-2-one (1h):

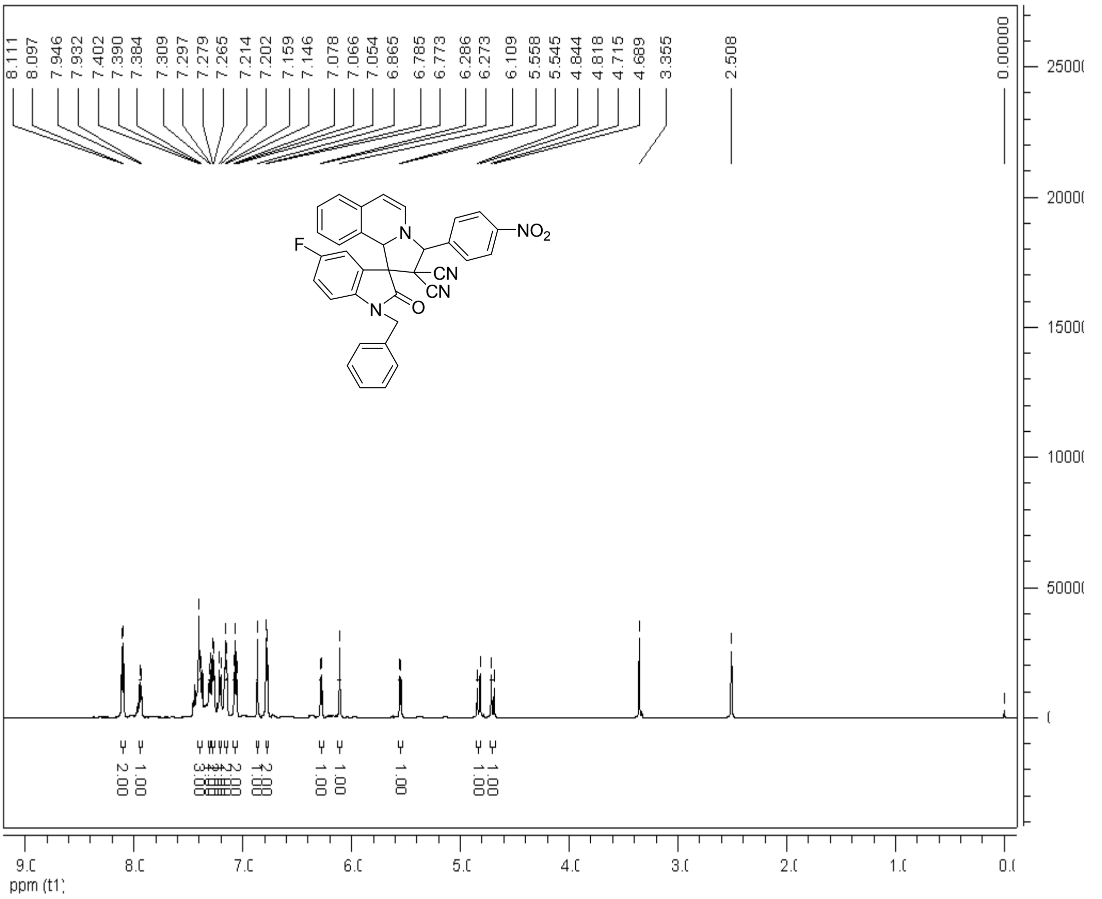

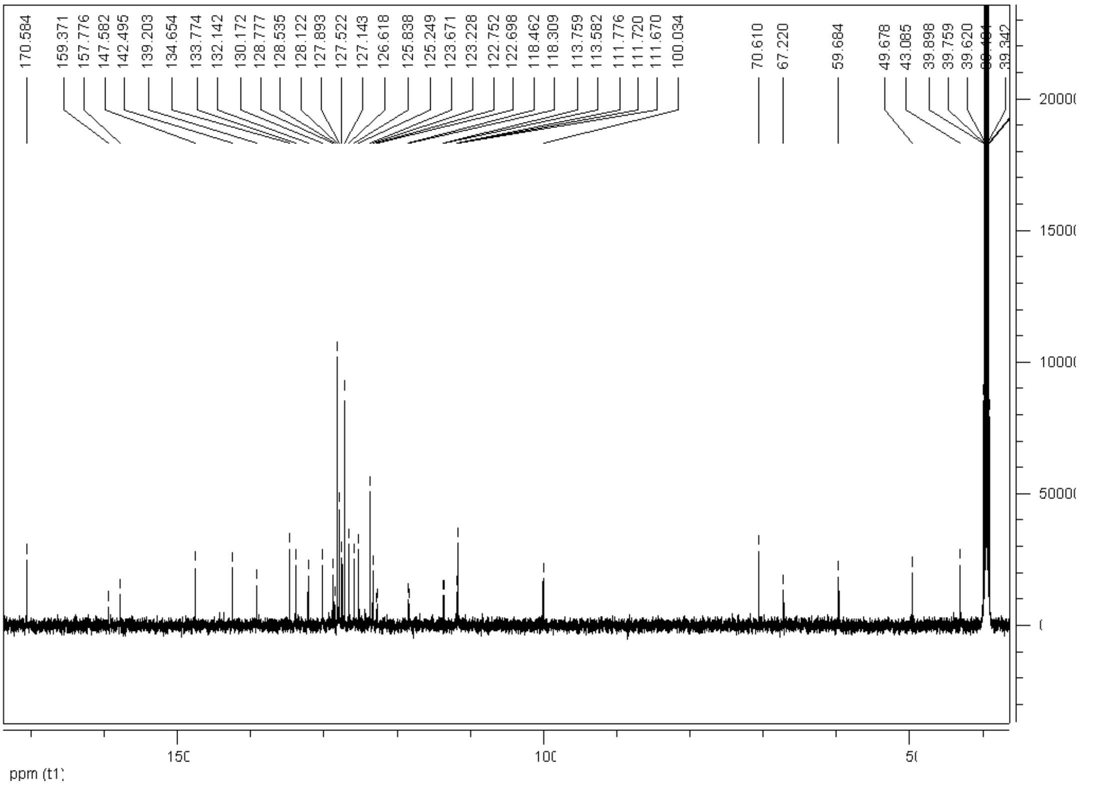

Ethyl 1-benzyl-1',1'-diisocyano-2-oxo-1',10b'-dihydro-3'H-spiro[indoline-3,2'-pyrrolo[2,1-a]isoquinoline]-3'-carboxylate (1i):

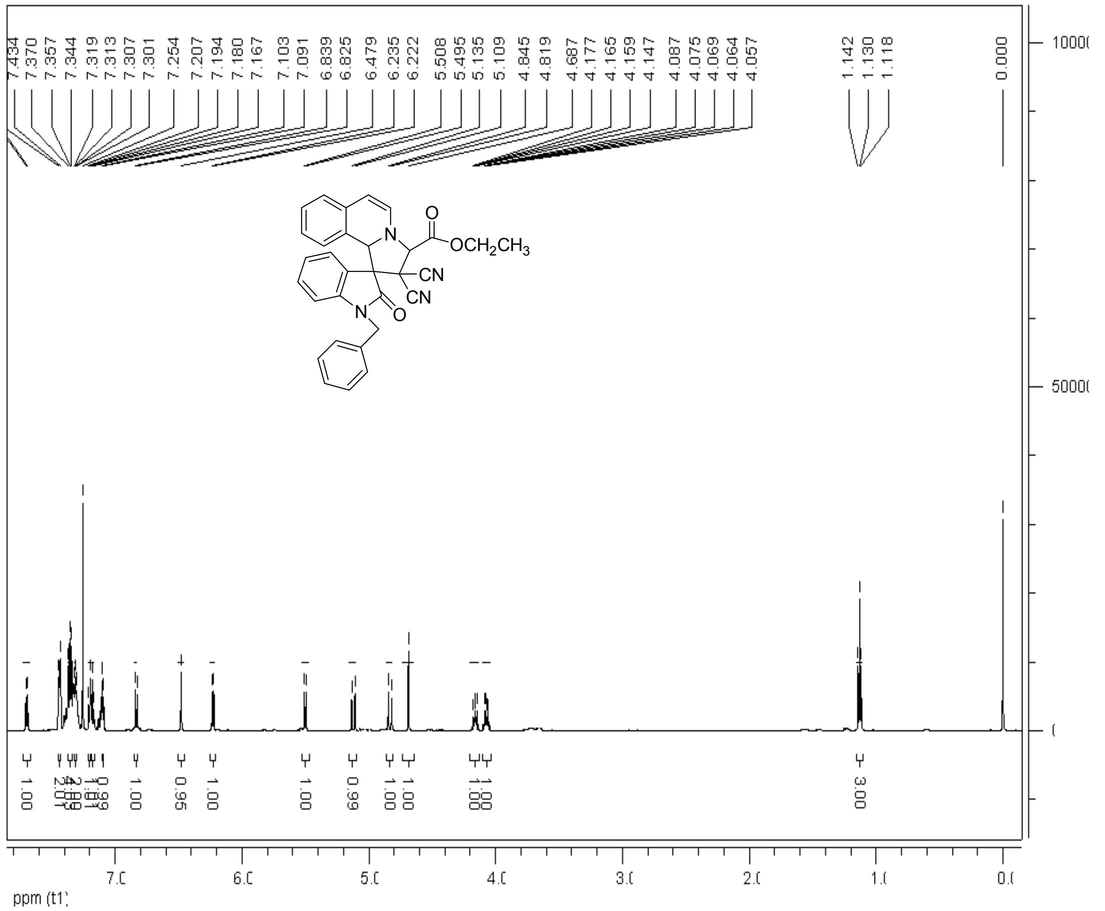

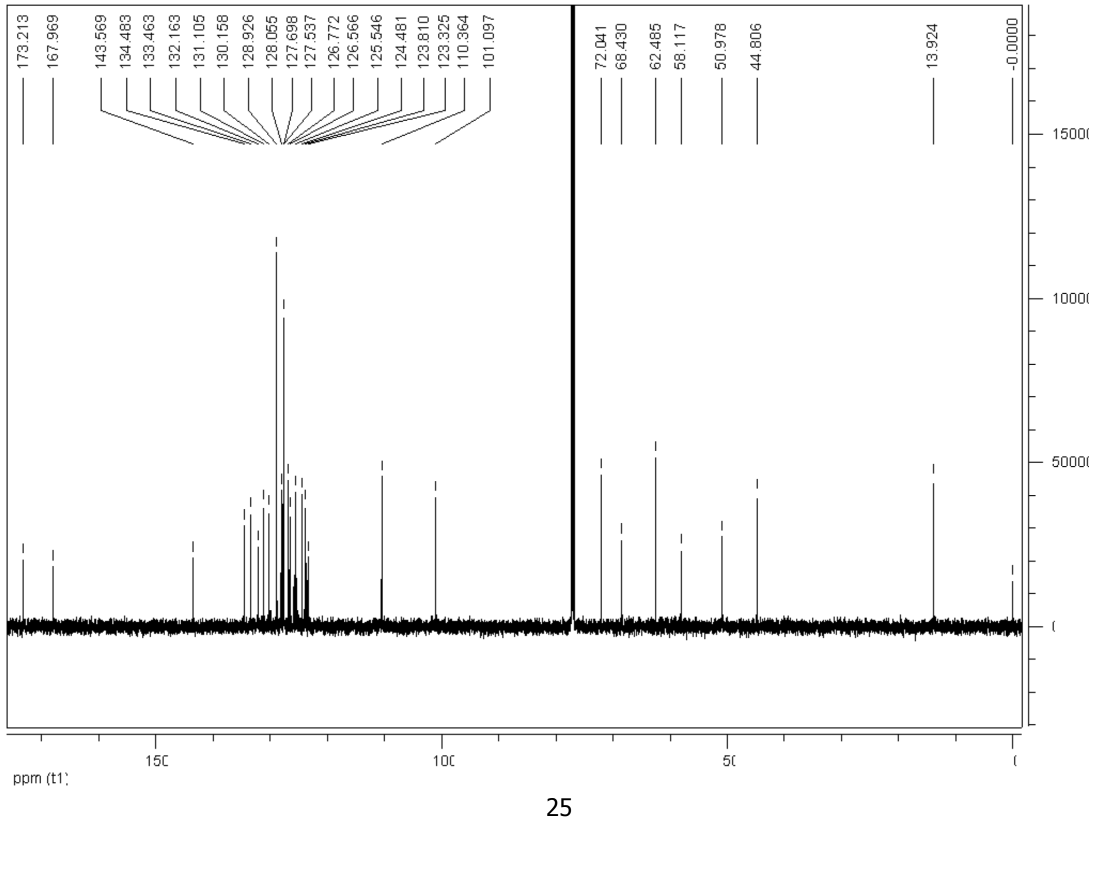

Ethyl 1-butyl-5-chloro-1',1'-diisocyano-2-oxo-1',10b'-dihydro-3'H-spiro[indoline-3,2'-pyrrolo[2,1-a]isoquinoline]-3'-carboxylate (1j):

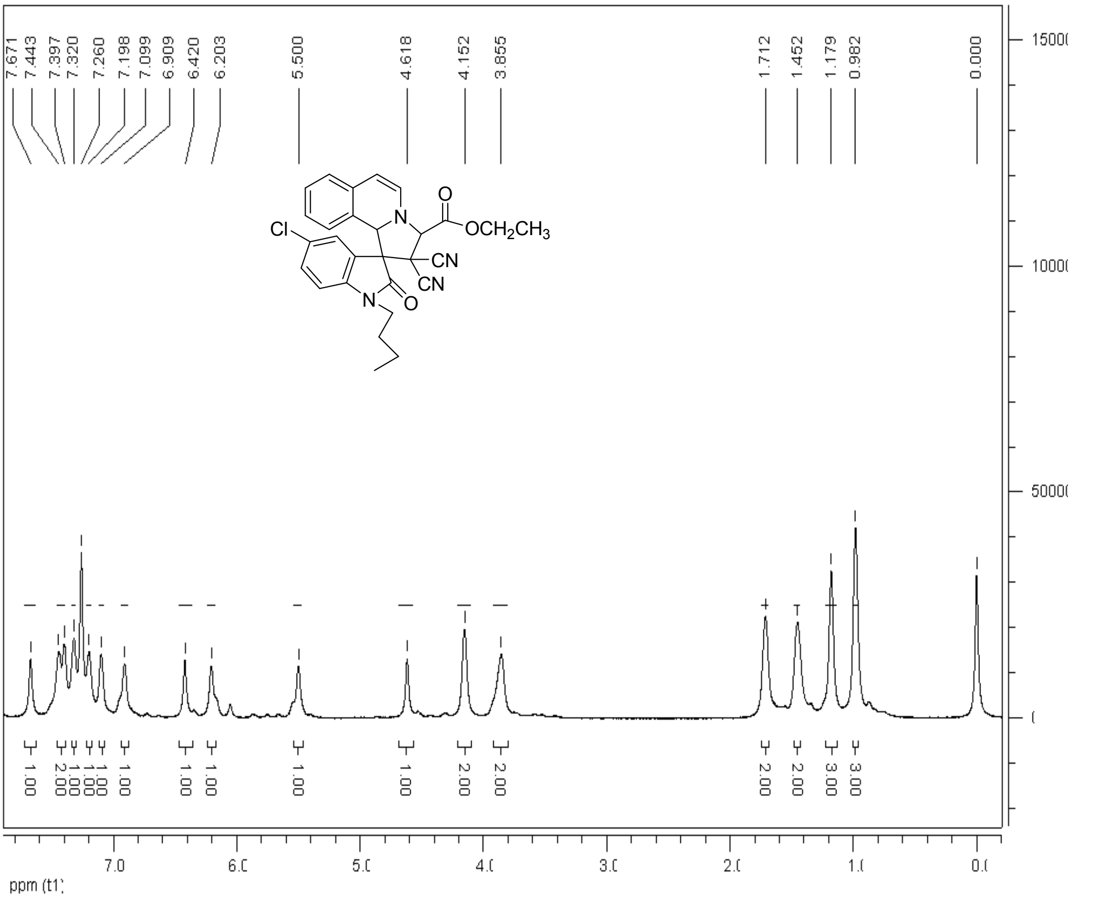

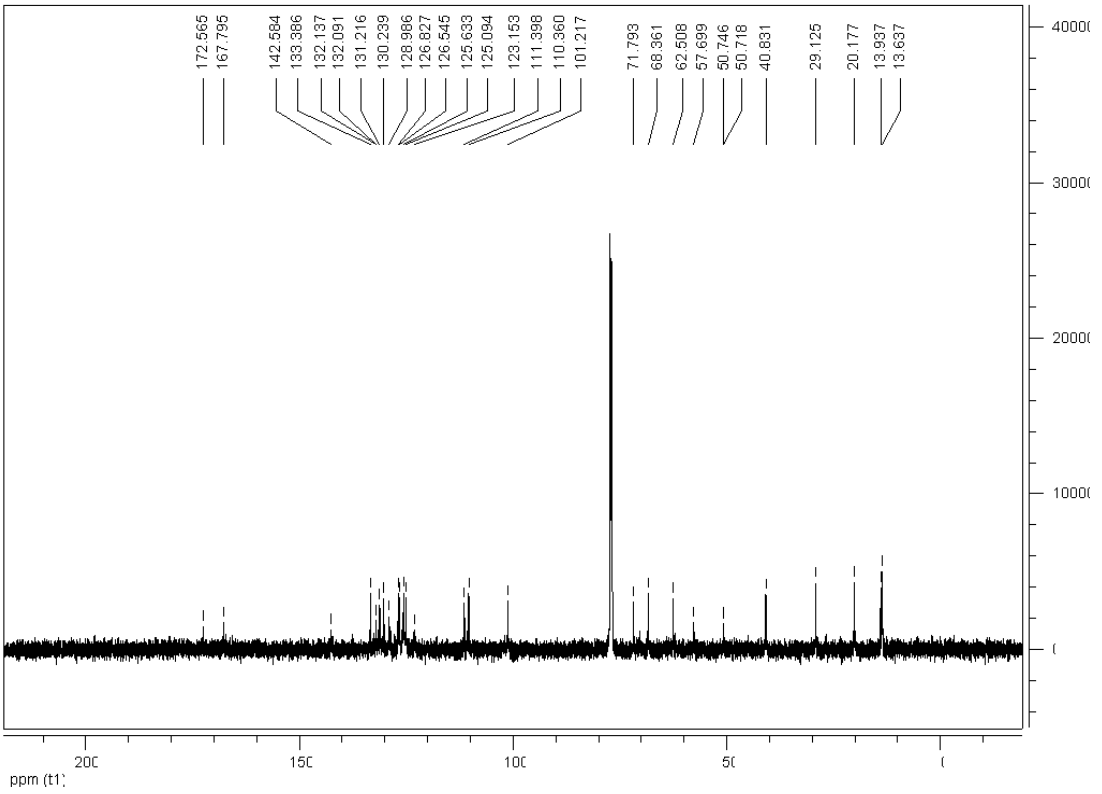

Ethyl 1-benzyl-5-chloro-1',1'-diisocyano-2-oxo-1',10b'-dihydro-3'H-spiro[indoline-3,2'-pyrrolo[2,1-a]isoquinoline]-3'-carboxylate (1k):

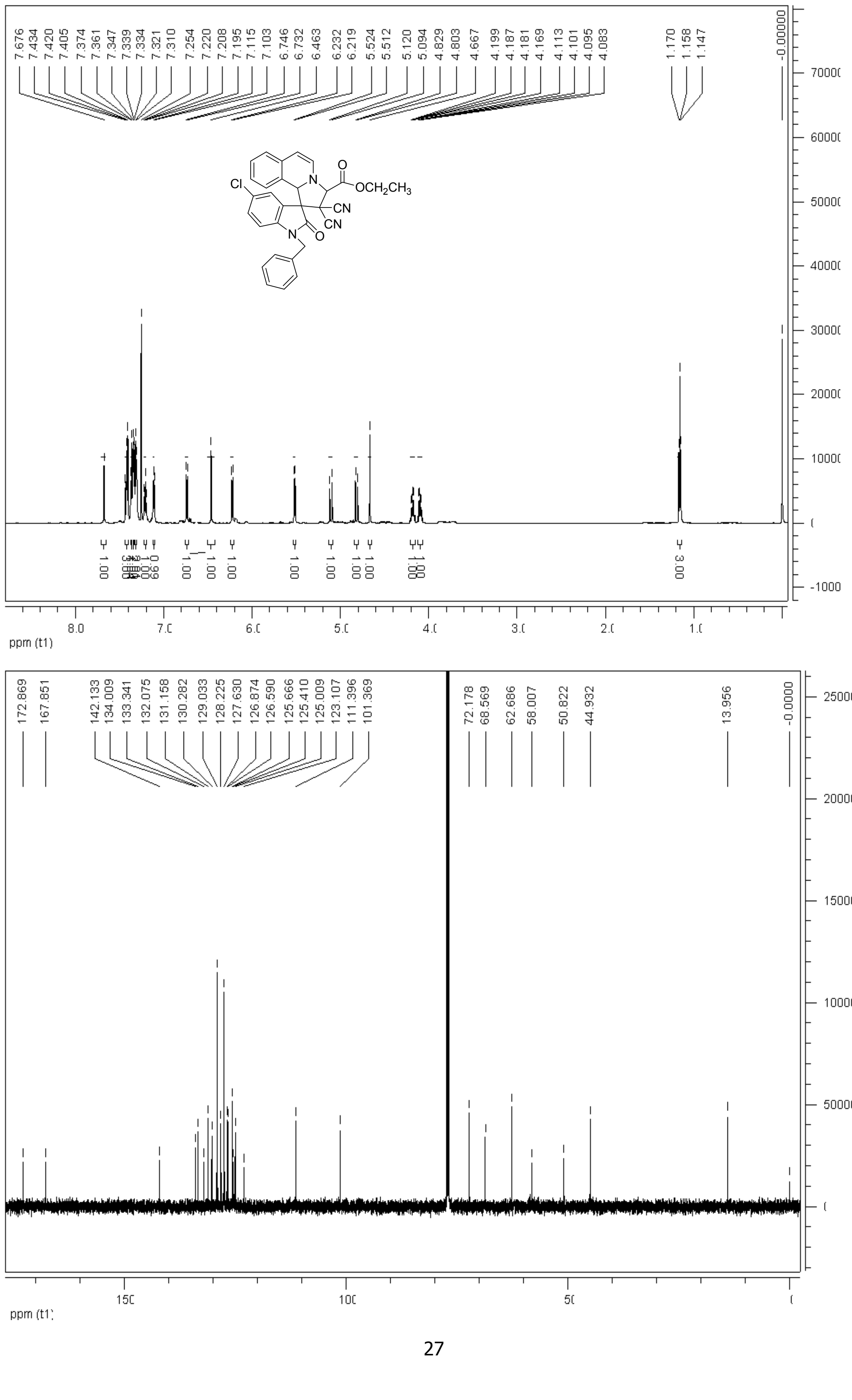

Ethyl 1-butyl-5-methyl-1',1'-diisocyano-2-oxo-1',10b'-dihydro-3'H-spiro[indoline-3,2'-pyrrolo[2,1-a]isoquinoline]-3'-carboxylate (1l):

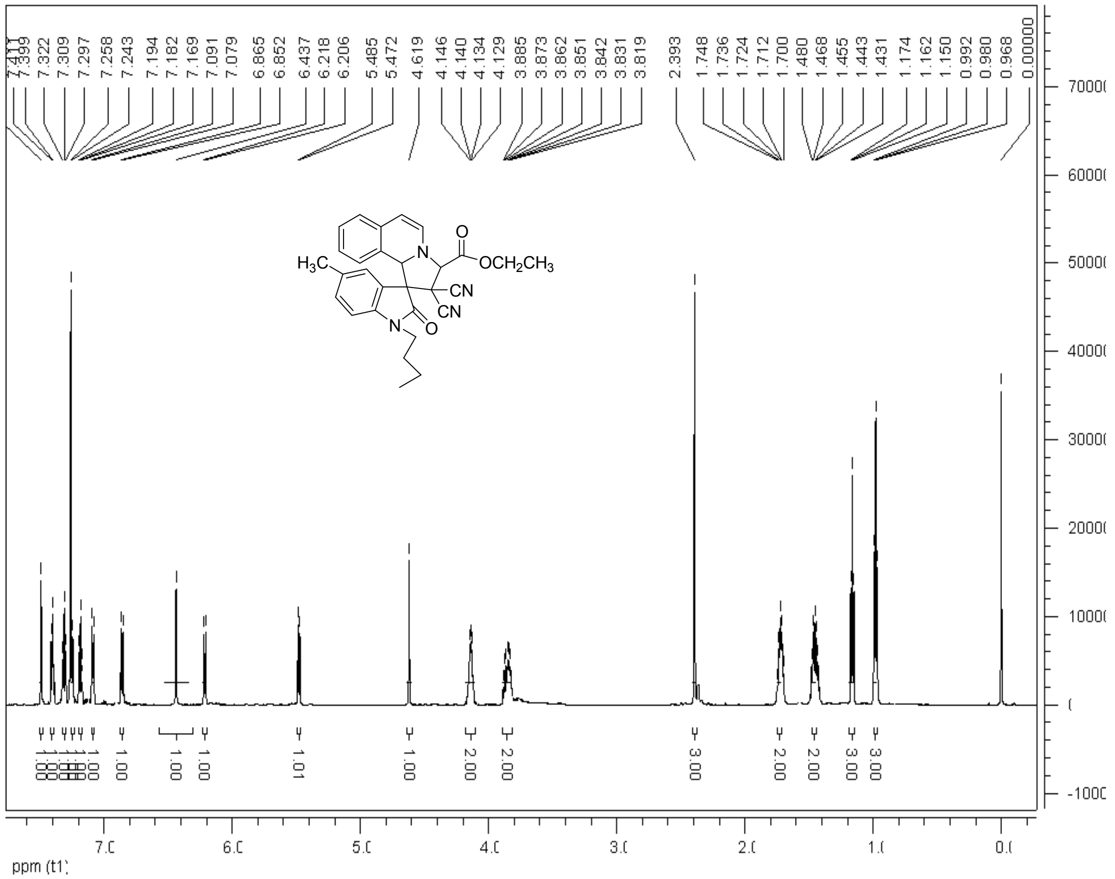

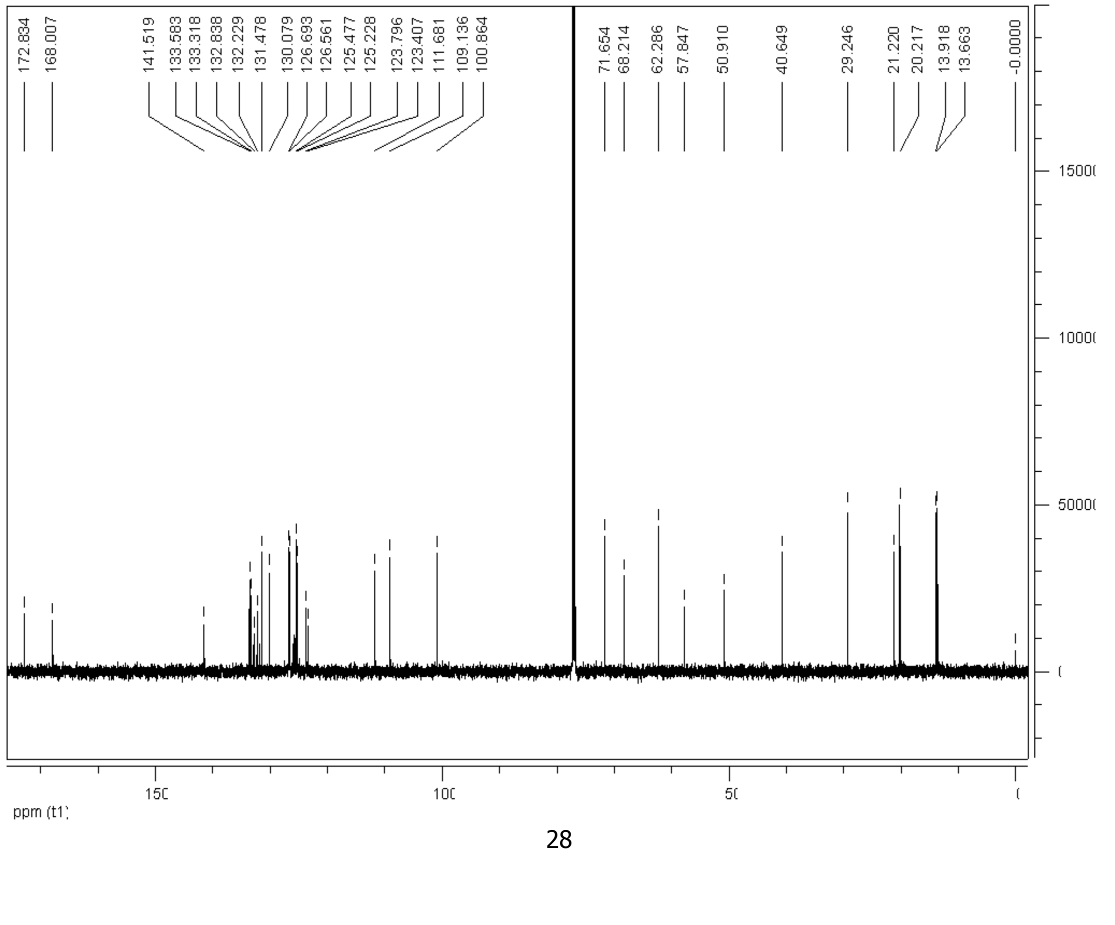

Ethyl 1-benzyl-5-methyl-1',1'-diisocyano-2-oxo-1',10b'-dihydro-3'H-spiro[indoline-3,2'-pyrrolo[2,1-a]isoquinoline]-3'-carboxylate (1m):

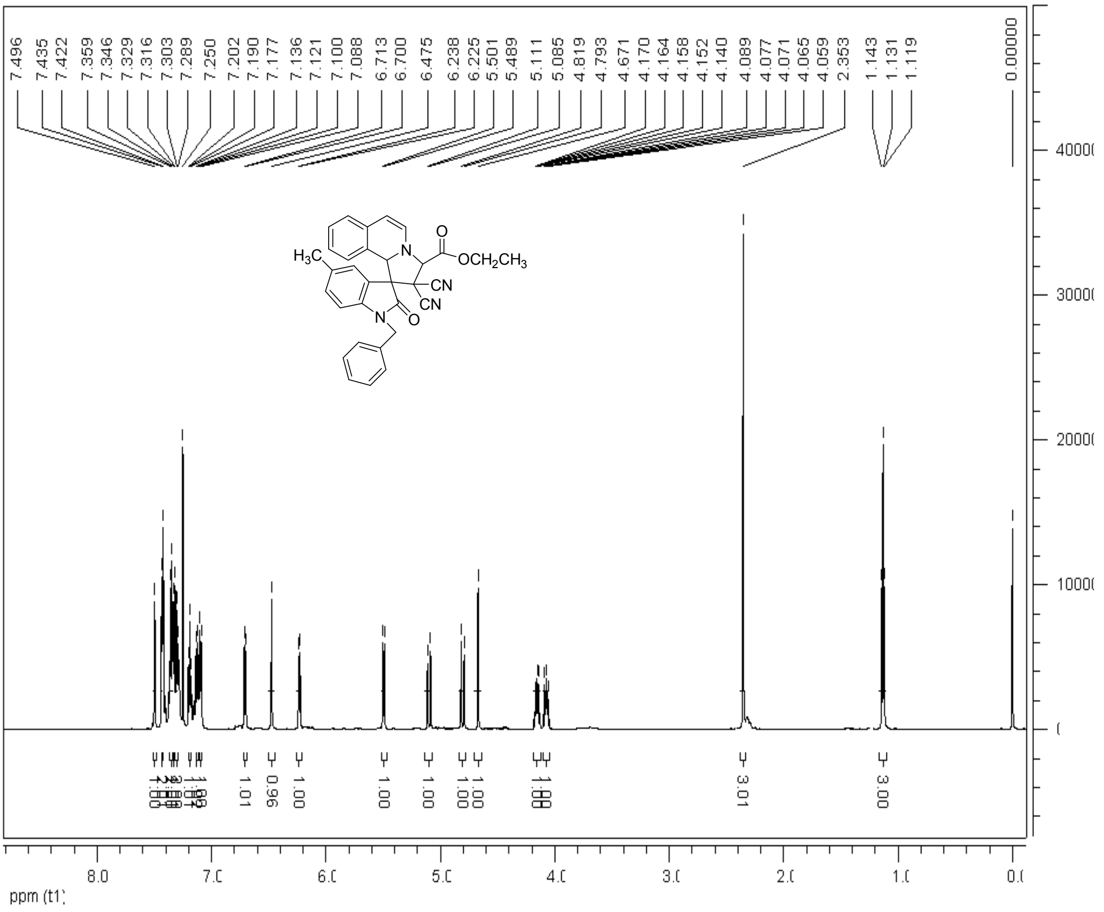

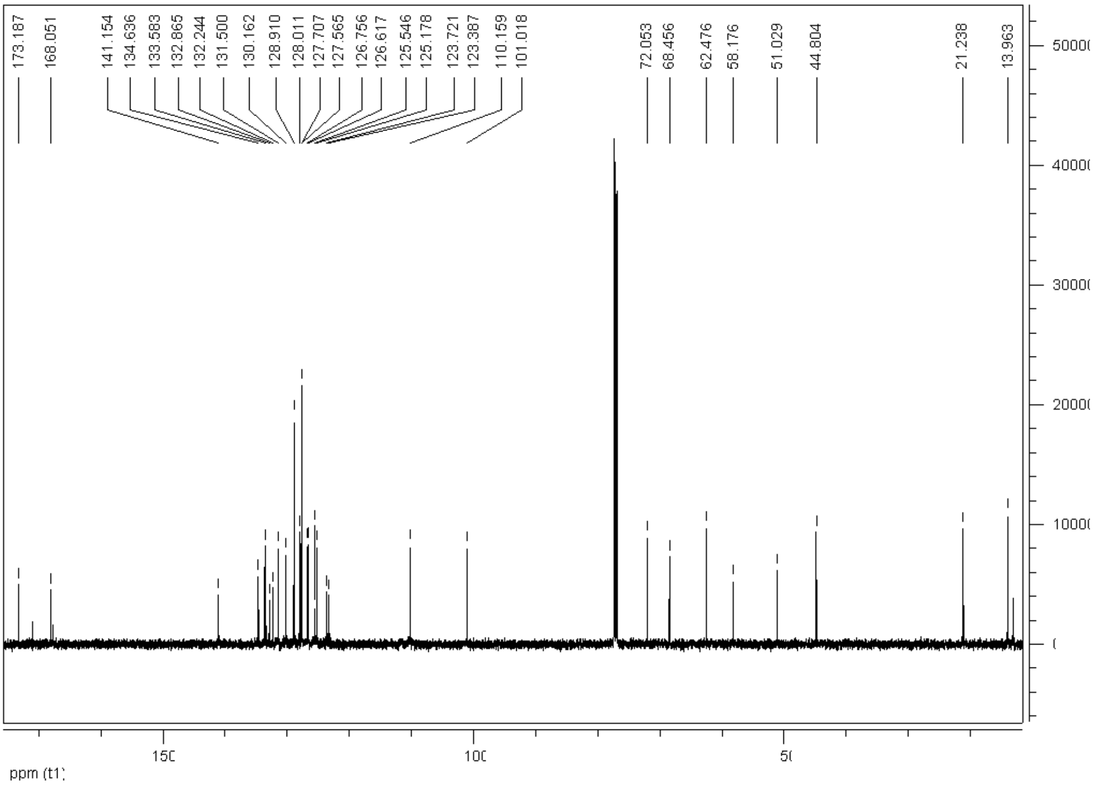

Ethyl 5-methyl-1',1'-diisocyano-2-oxo-1',10b'-dihydro-3'H-spiro[indoline-3,2'-pyrrolo[2,1-a]isoquinoline]-3'-carboxylate (1n):

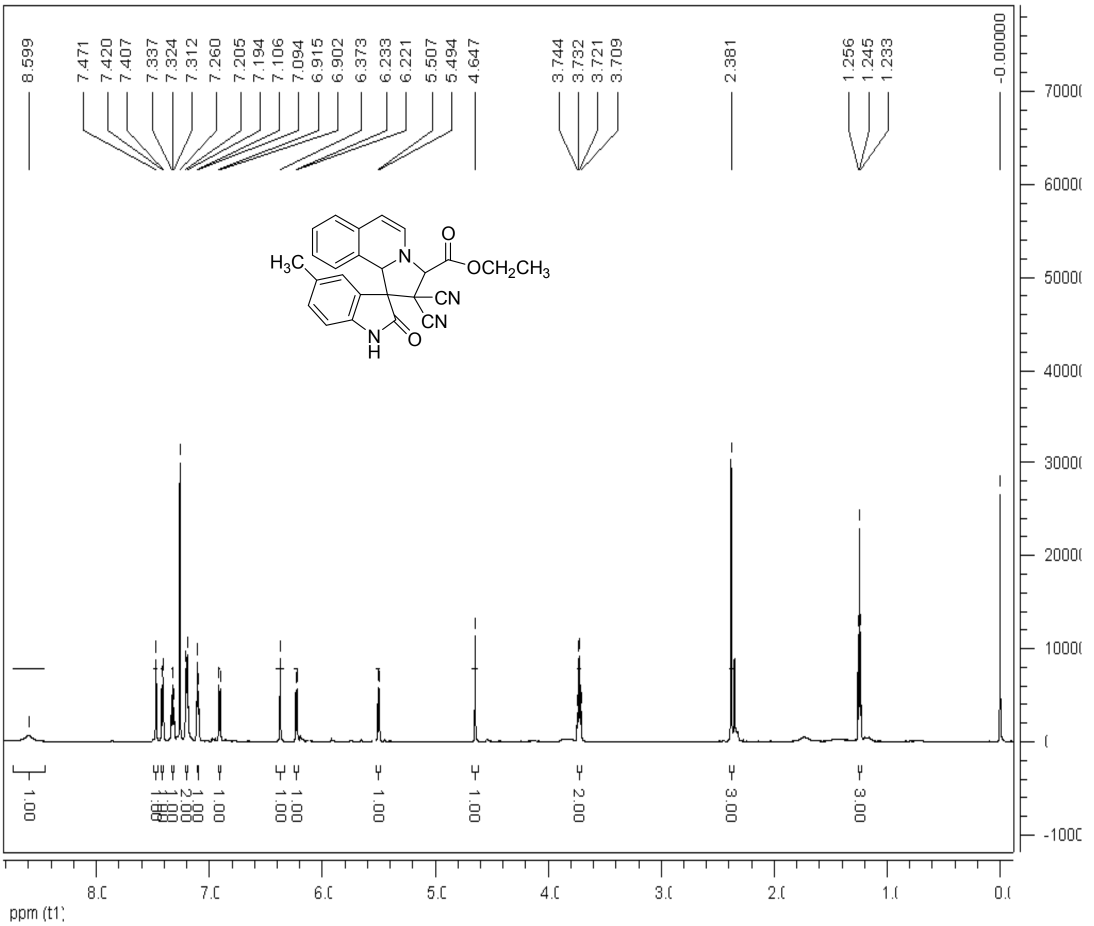

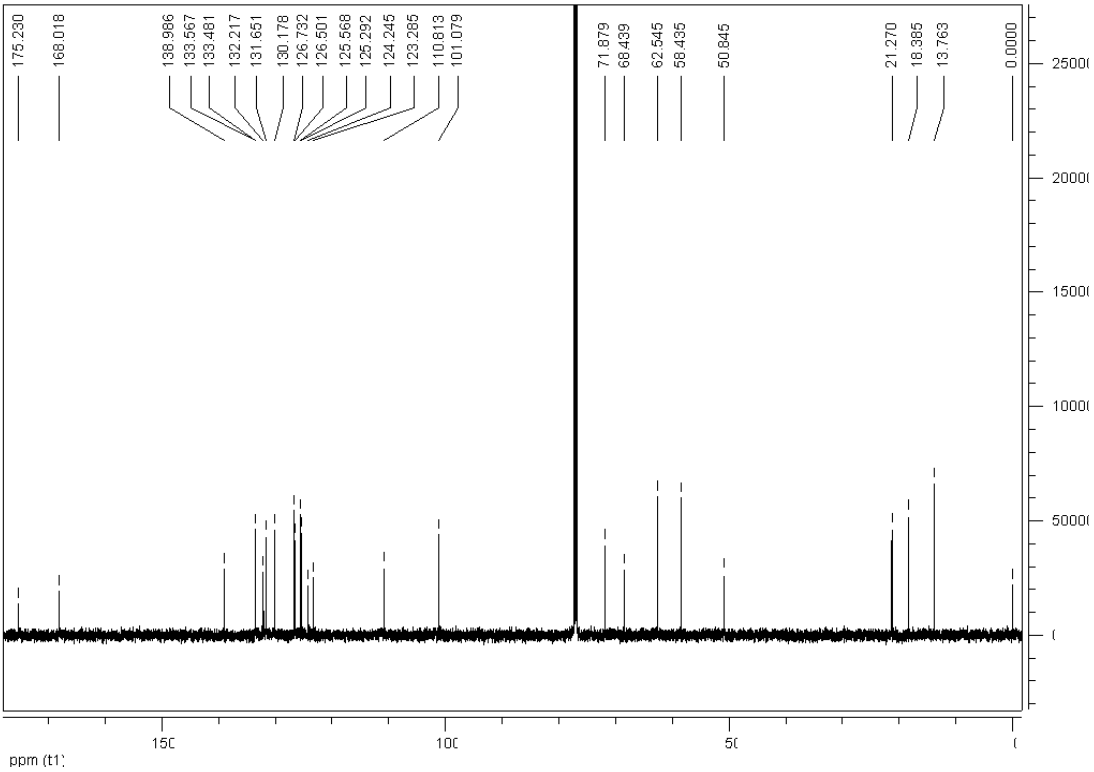

Ethyl 1-butyl-5-fluoro-1',1'-diisocyano-2-oxo-1',10b'-dihydro-3'H-spiro[indoline-3,2'-pyrrolo[2,1-a]isoquinoline]-3'-carboxylate (1o):

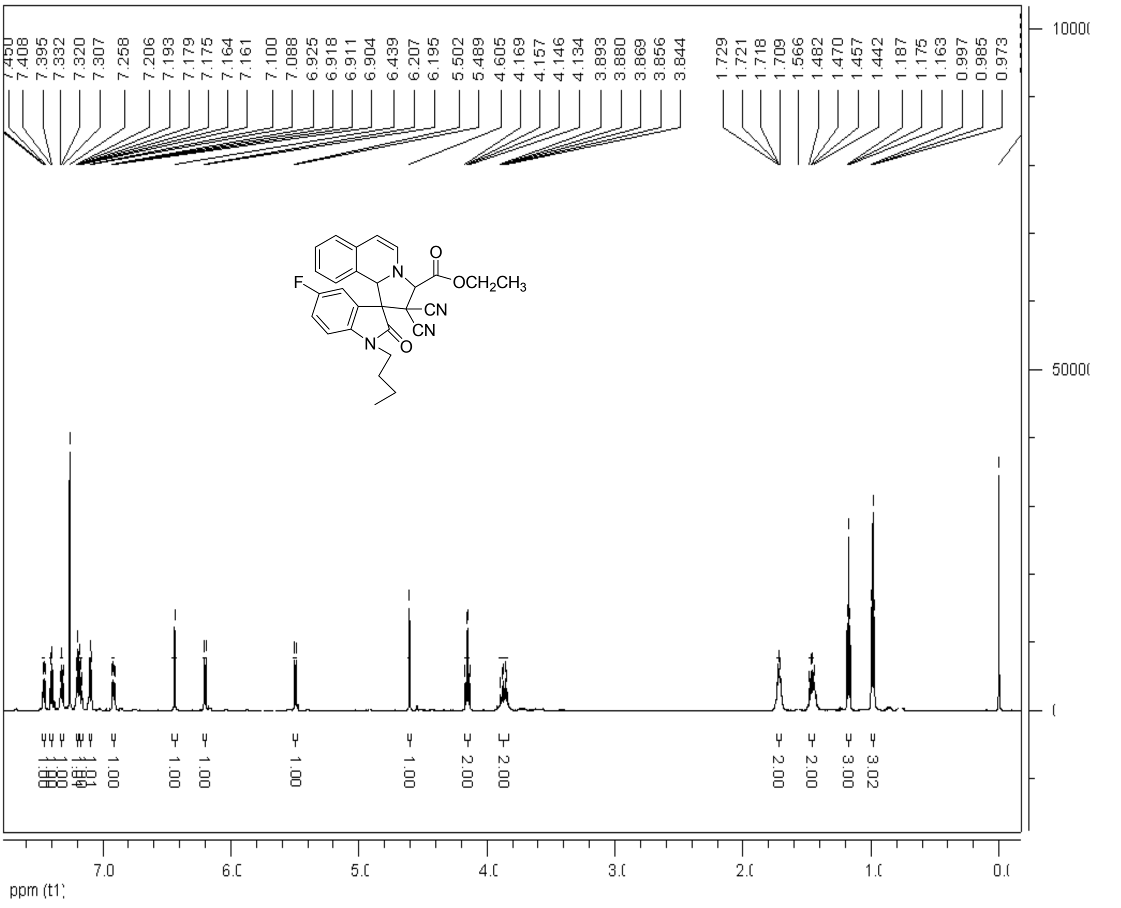

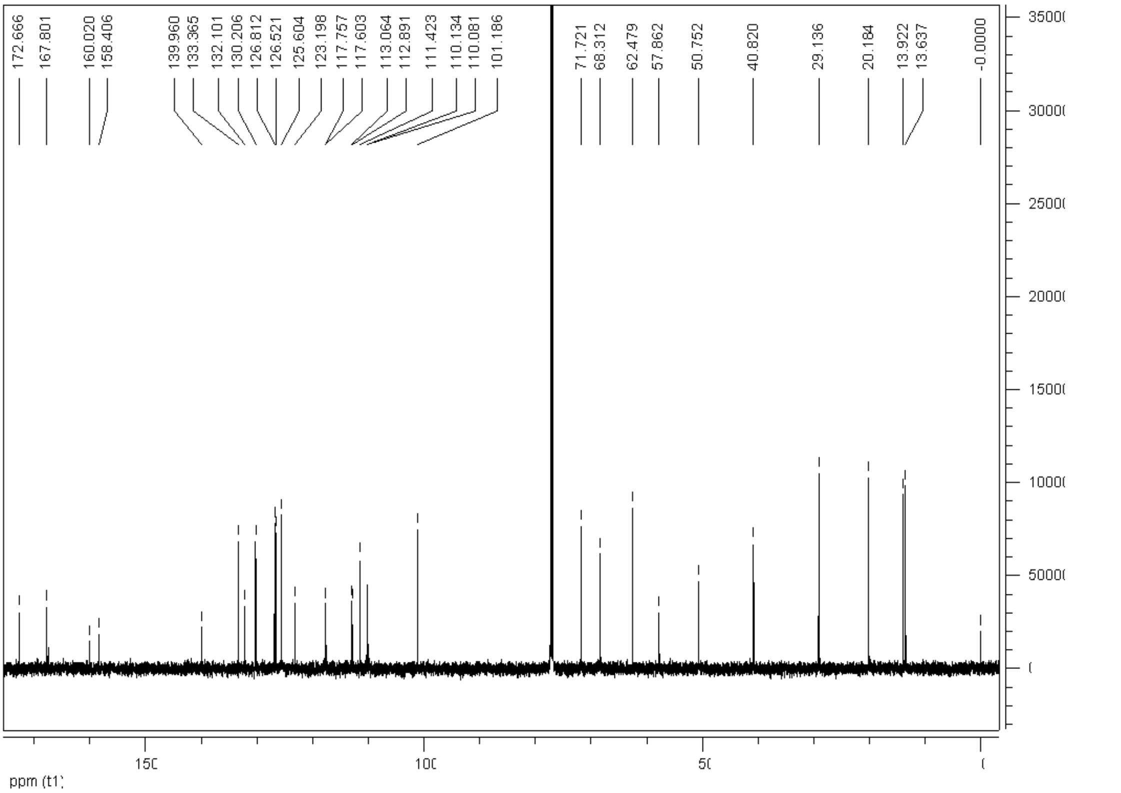

15-cyano-16-oxo-14b,15-dihydro-5a,15-(epiminomethano)indolo[2'',3'':2',3']pyrrolo[3',4':4,5]pyrrolo[2,1-a]isoquinolin-7(5H,6H,7aH)-ylidene)malononitrile (2a):

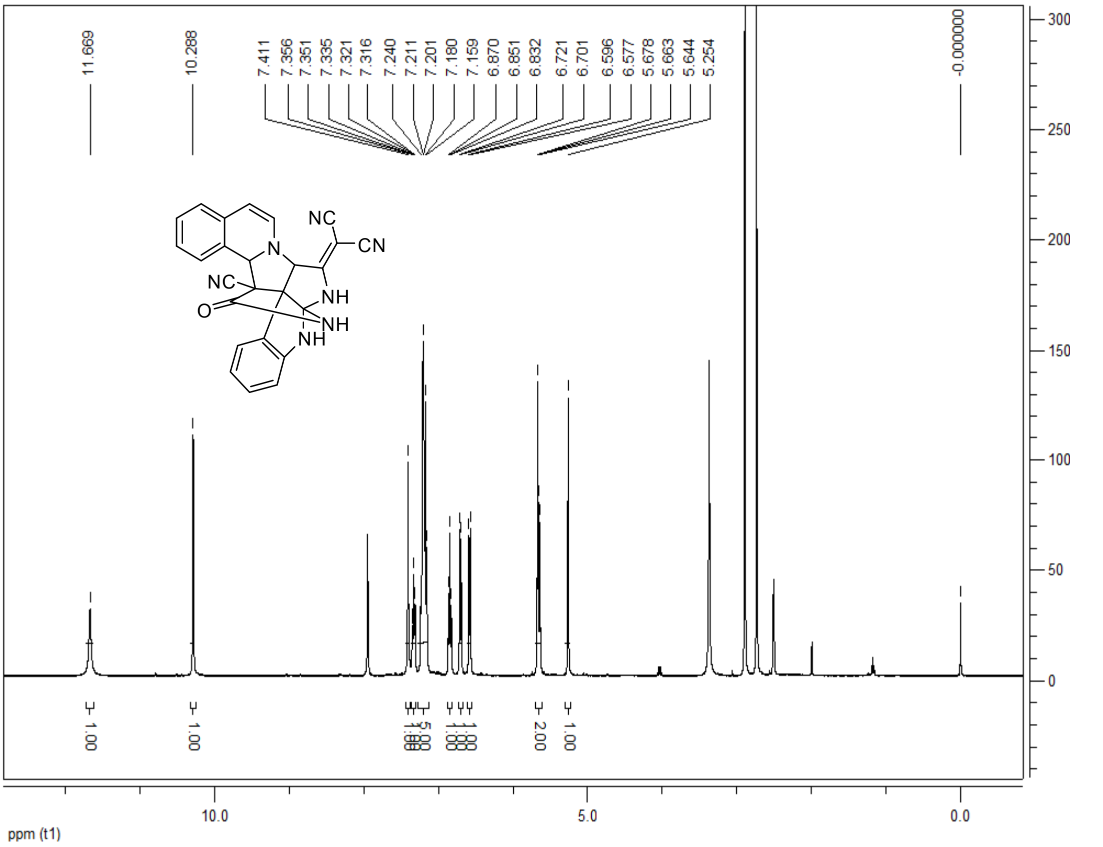

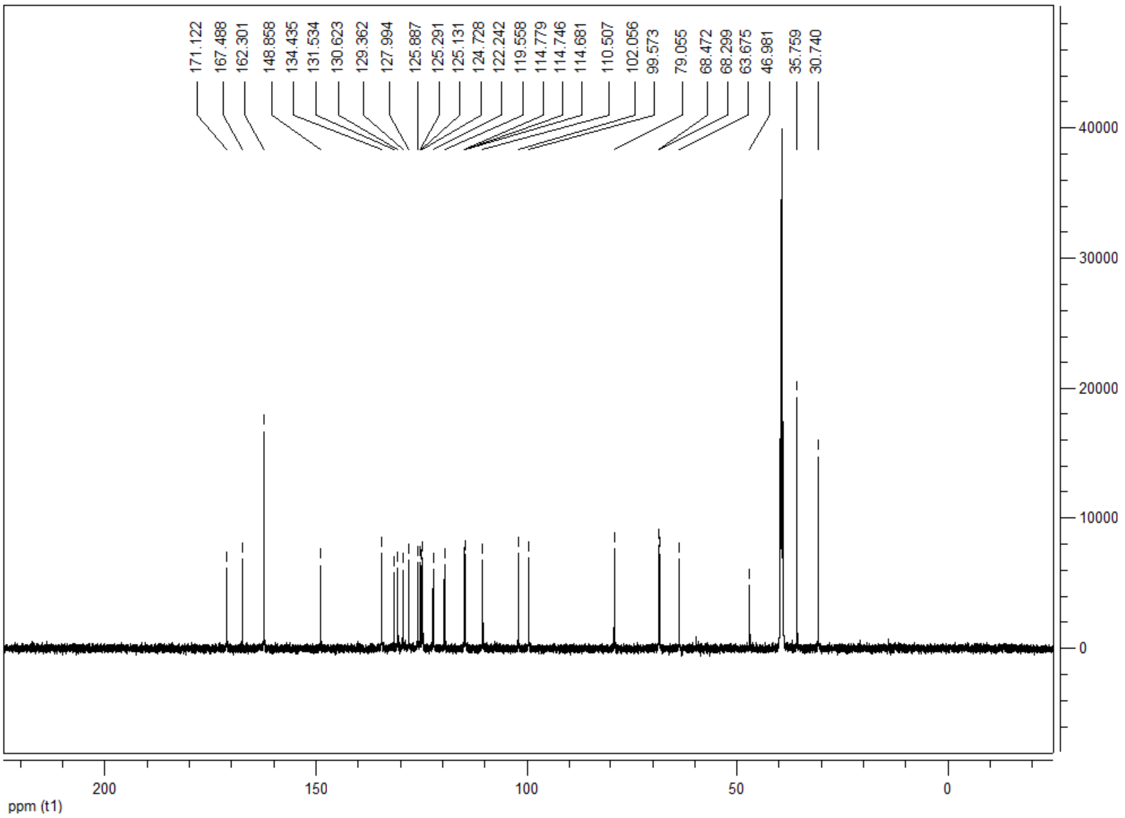

15-cyano-2-fluoro-16-oxo-14b,15-dihydro-5a,15-(epiminomethano)indolo[2'',3'':2',3']pyrrolo [3',4':4,5]pyrrolo[2,1-a]isoquinolin-7(5H,6H,7aH)-ylidene)malononitrile (2b):

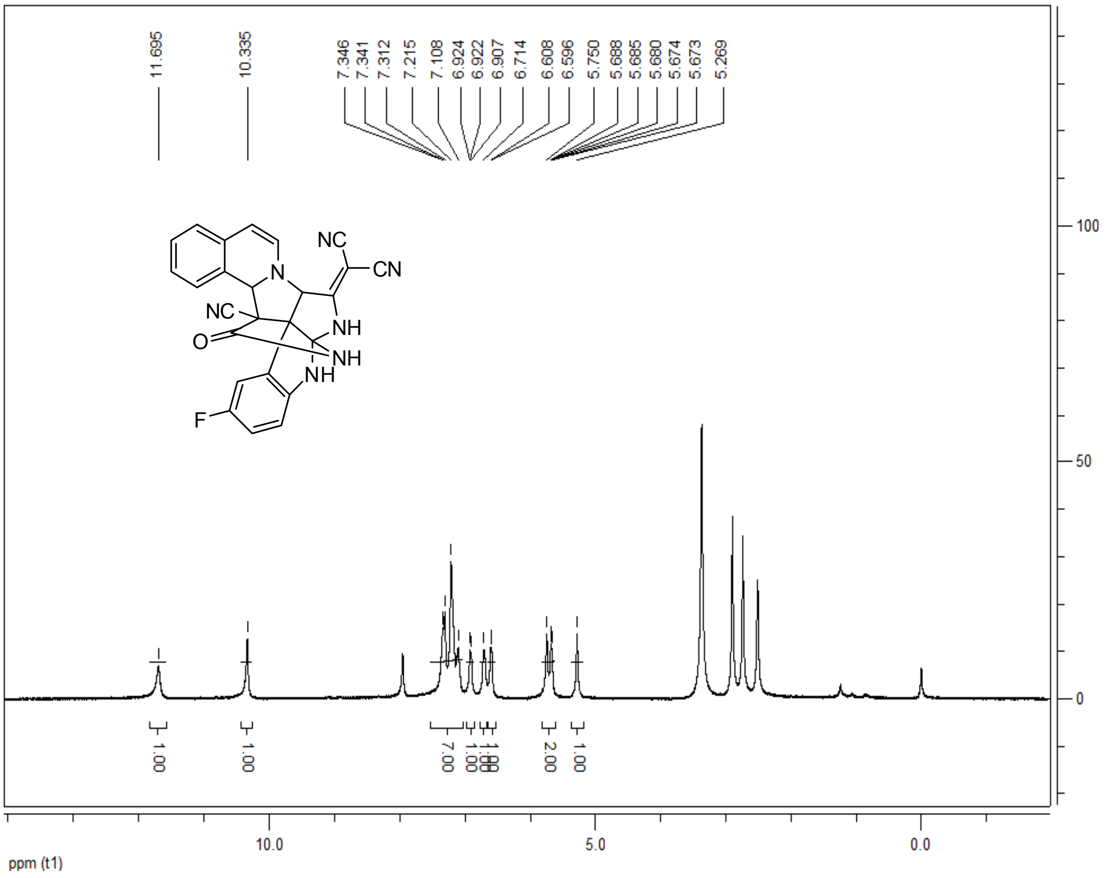

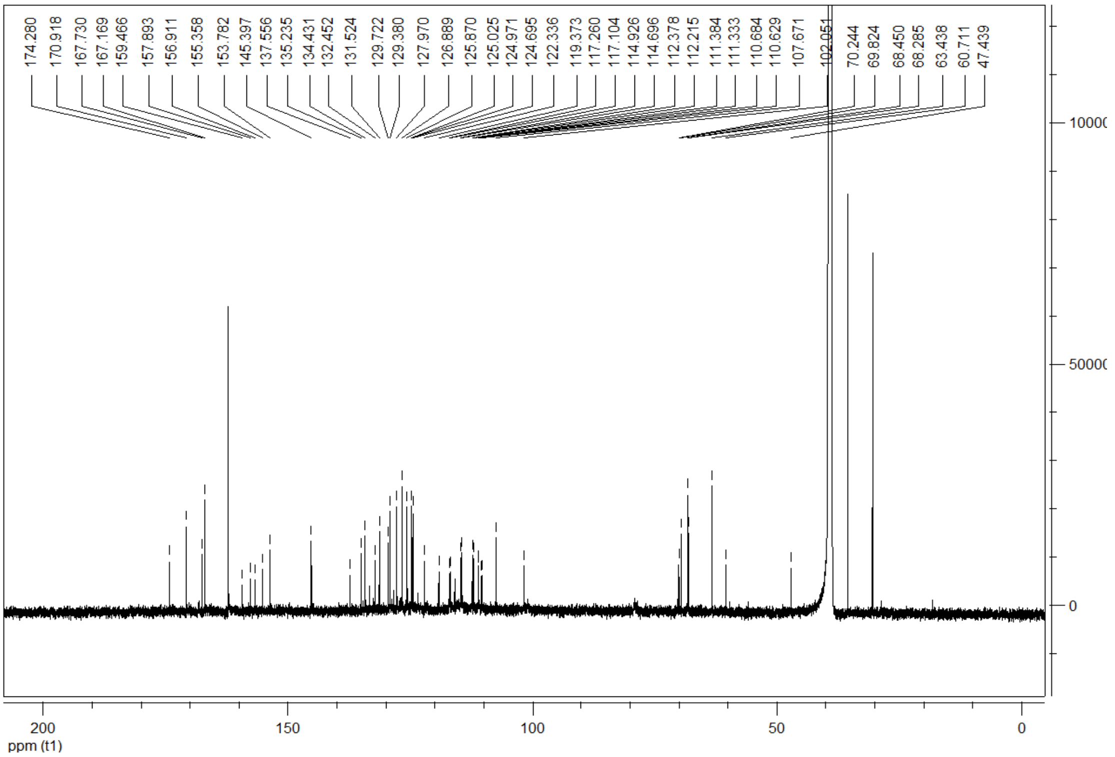

15-cyano-2-methyl-16-oxo-14b,15-dihydro-5a,15-(epiminomethano)indolo[2'',3'':2',3']pyrrolo[3',4':4,5]pyrrolo[2,1-a]isoquinolin-7(5H,6H,7aH)-ylidene)malononitrile (2c):

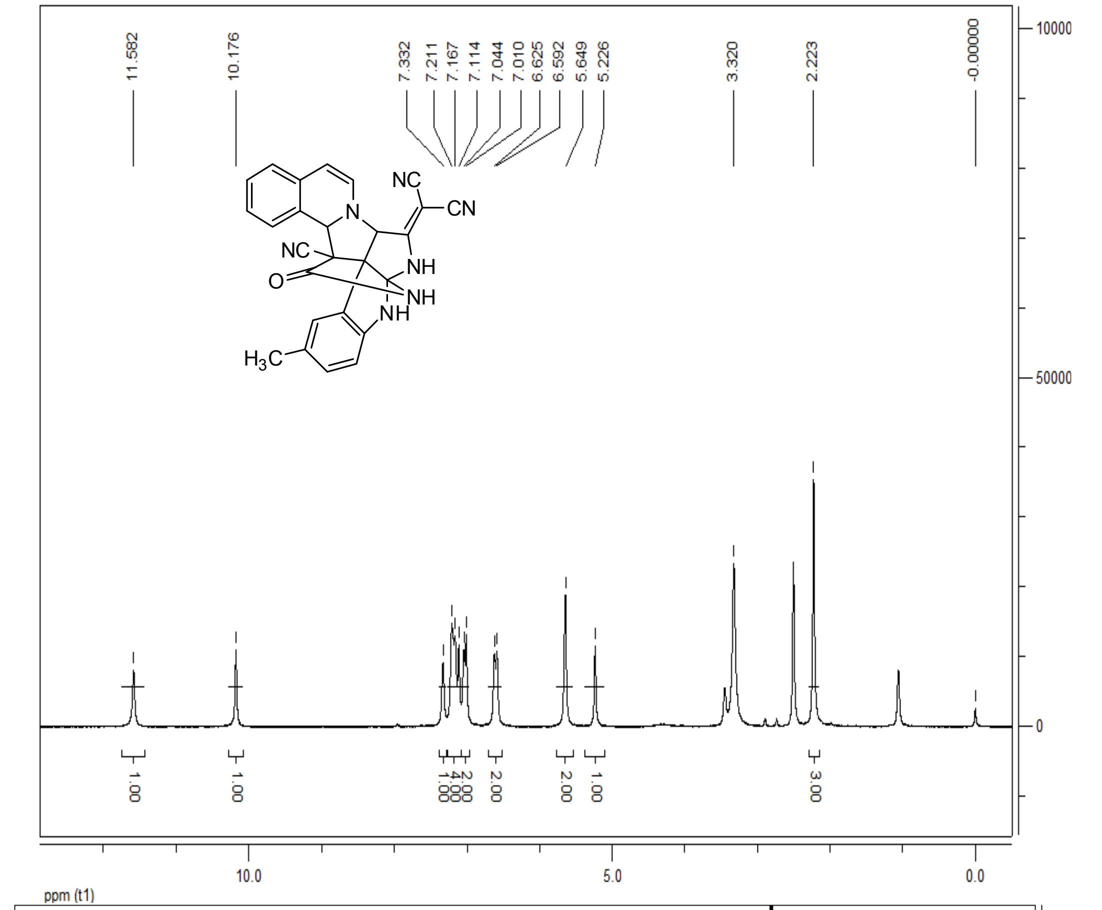

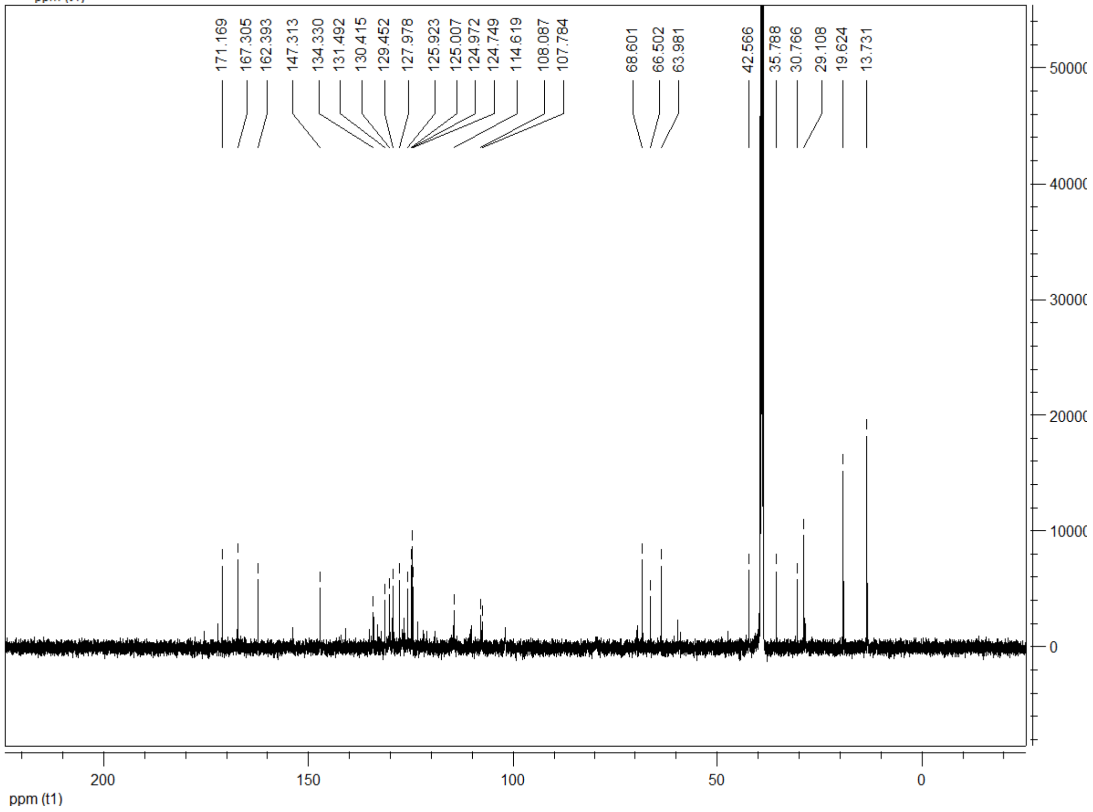

5-benzyl-15-cyano-16-oxo-14b,15-dihydro-5a,15-(epiminomethano)indolo[2'',3'':2',3']pyrrolo[3',4':4,5]pyrrolo[2,1-a]isoquinolin-7(5H,6H,7aH)-ylidene)malononitrile (2d):

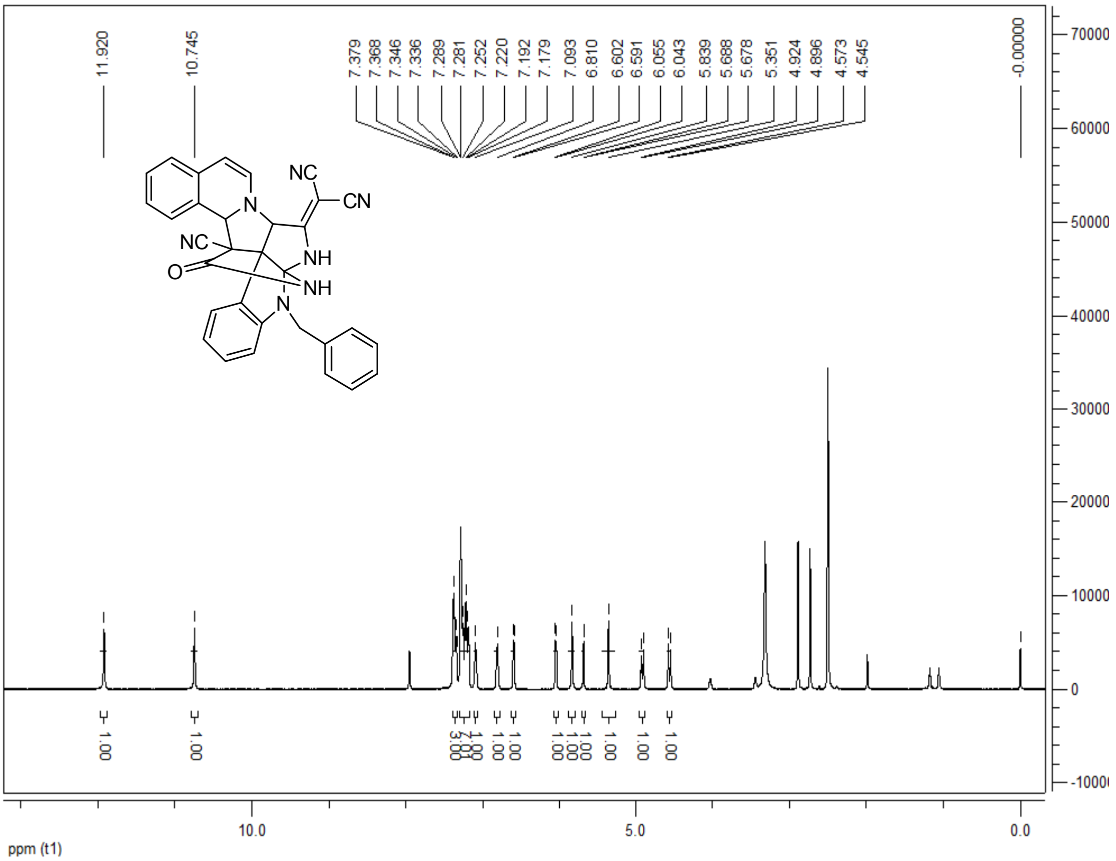

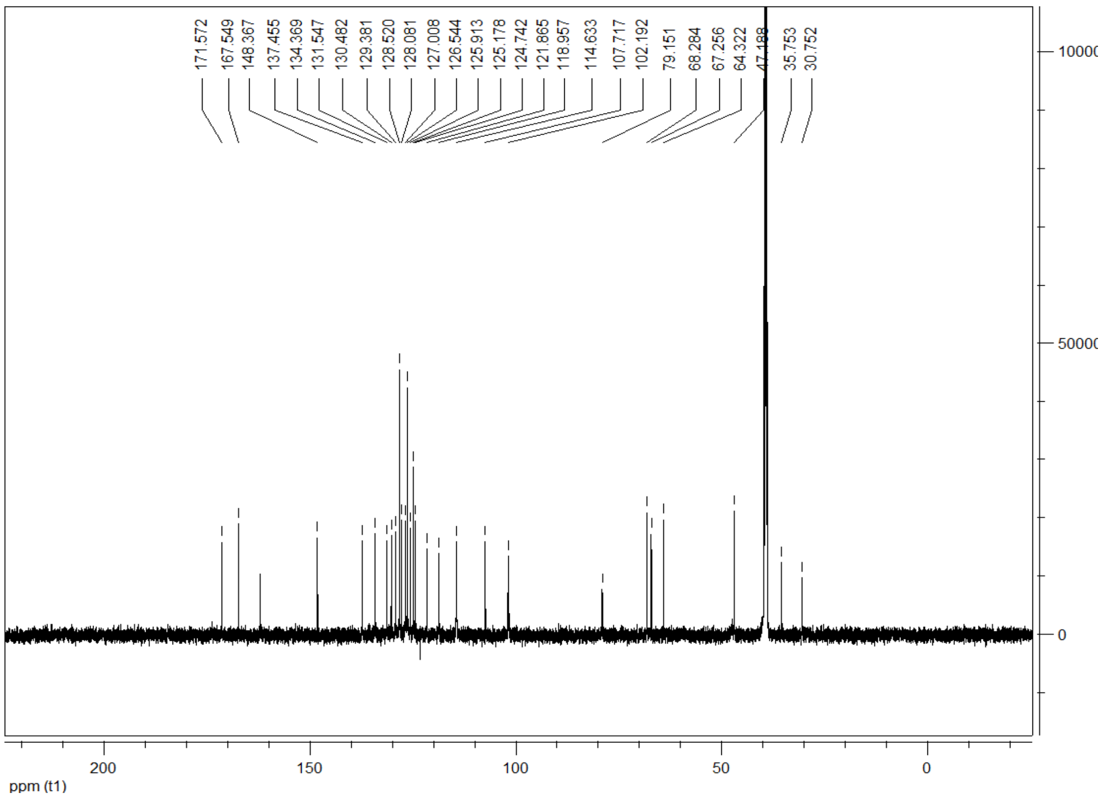

5-benzyl-2-chloro-15-cyano-16-oxo-14b,15-dihydro-5a,15-(epiminomethano)indolo[2'',3'':2',3']pyrrolo[3',4':4,5]pyrrolo[2,1-a]isoquinolin-7(5H,6H,7aH)-ylidene)malononitrile (2e):

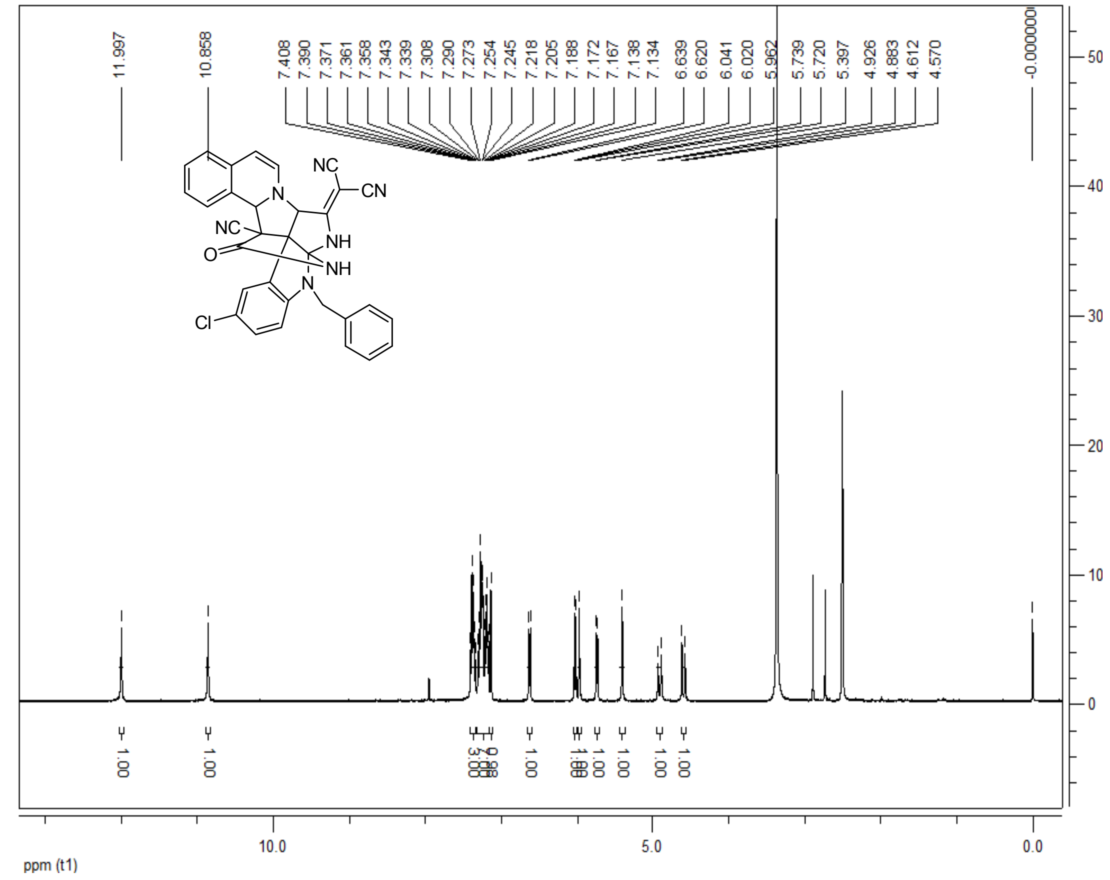

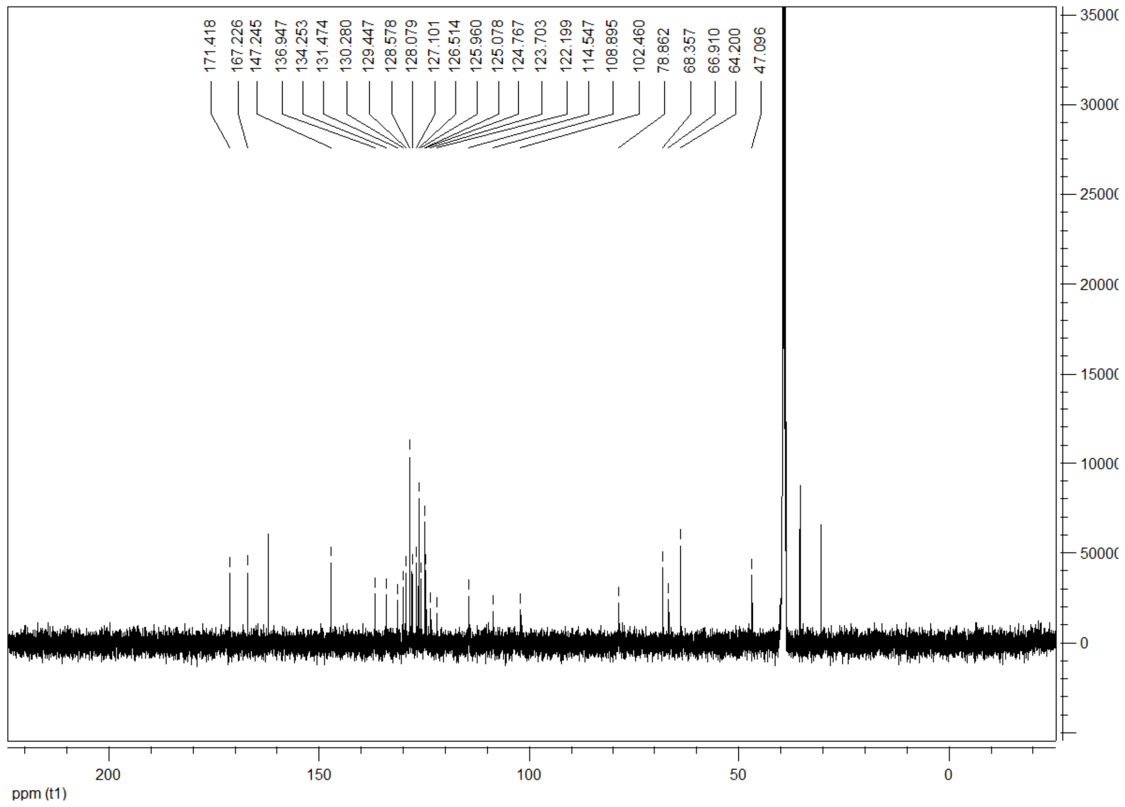

5-benzyl-15-cyano-2-fluoro-16-oxo-14b,15-dihydro-5a,15-(epiminomethano)indolo[2'',3'':2',3']pyrrolo[3',4':4,5]pyrrolo[2,1-a]isoquinolin-7(5H,6H,7aH)-ylidene)malononitrile (2f):

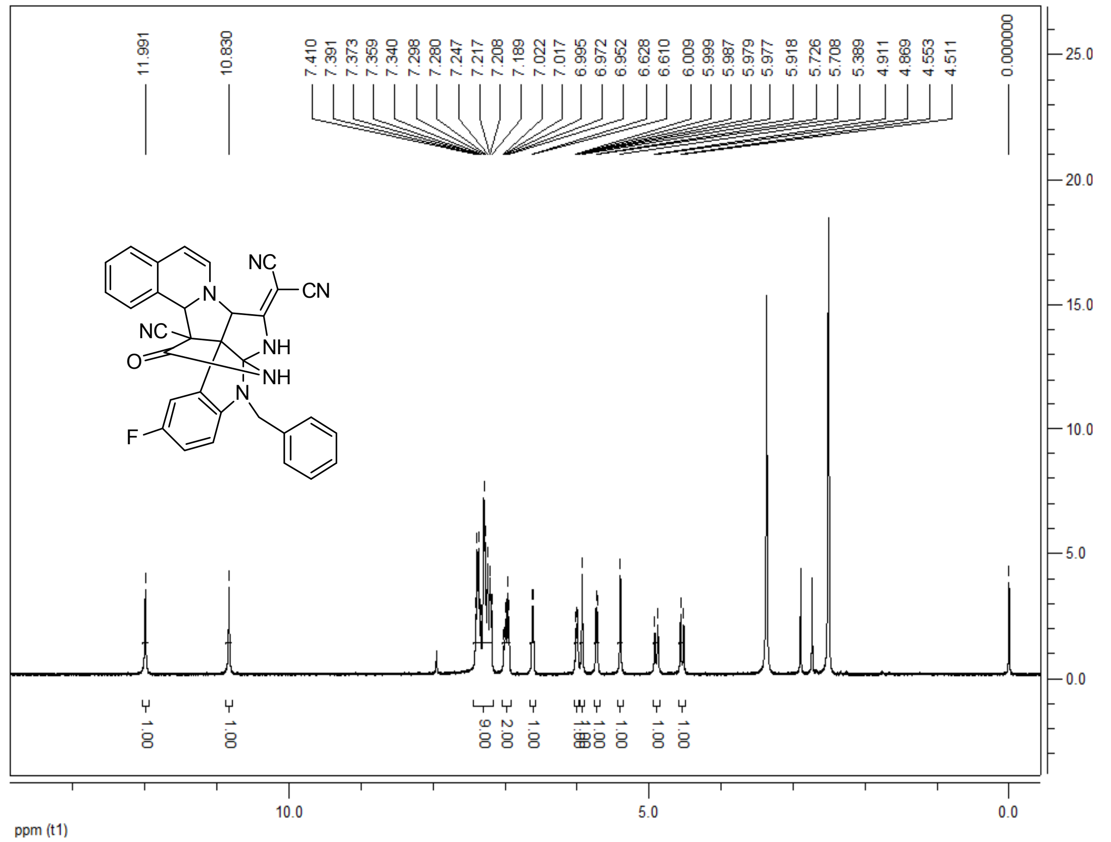

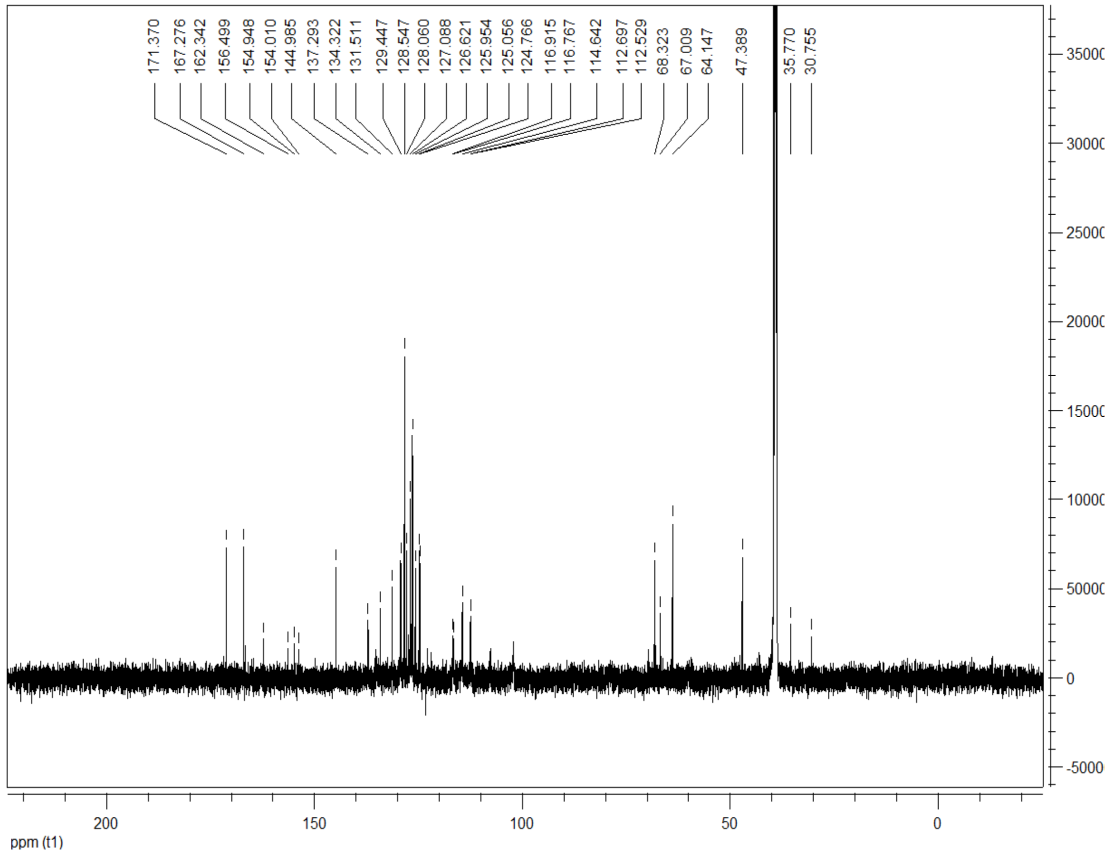

5-benzyl-15-cyano-2-methyl-16-oxo-14b,15-dihydro-5a,15-(epiminomethano)indolo[2'',3'':2',3']pyrrolo[3',4':4,5]pyrrolo[2,1-a]isoquinolin-7(5H,6H,7aH)-ylidene)malononitrile (2g):

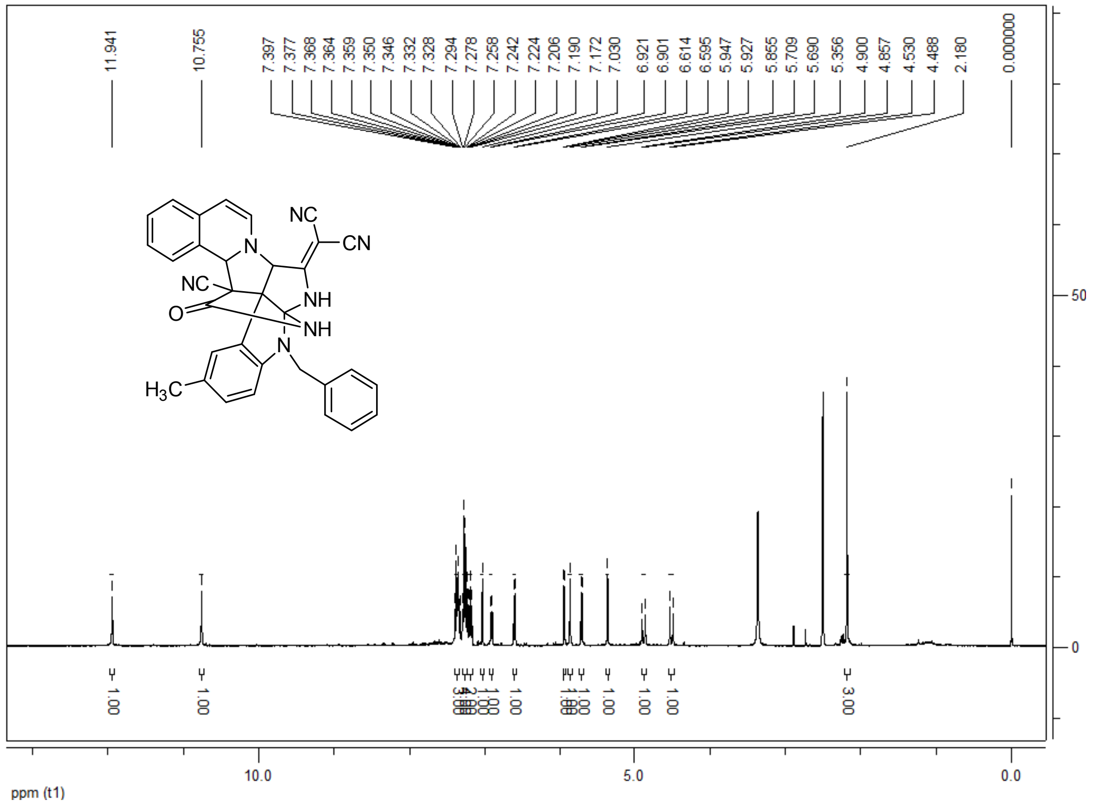

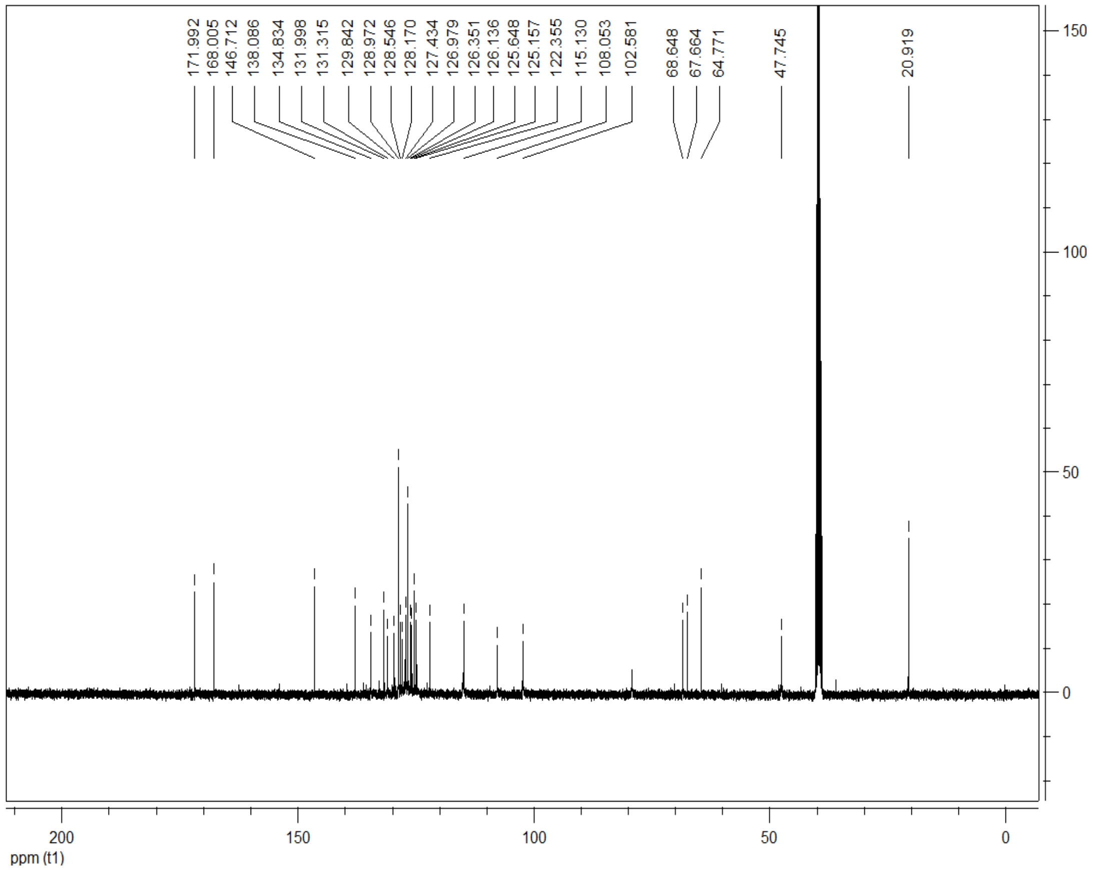

5-butyl-15-cyano-16-oxo-14b,15-dihydro-5a,15-(epiminomethano)indolo[2'',3'':2',3']pyrrolo[3',4':4,5]pyrrolo[2,1-a]isoquinolin-7(5H,6H,7aH)-ylidene)malononitrile (2h):

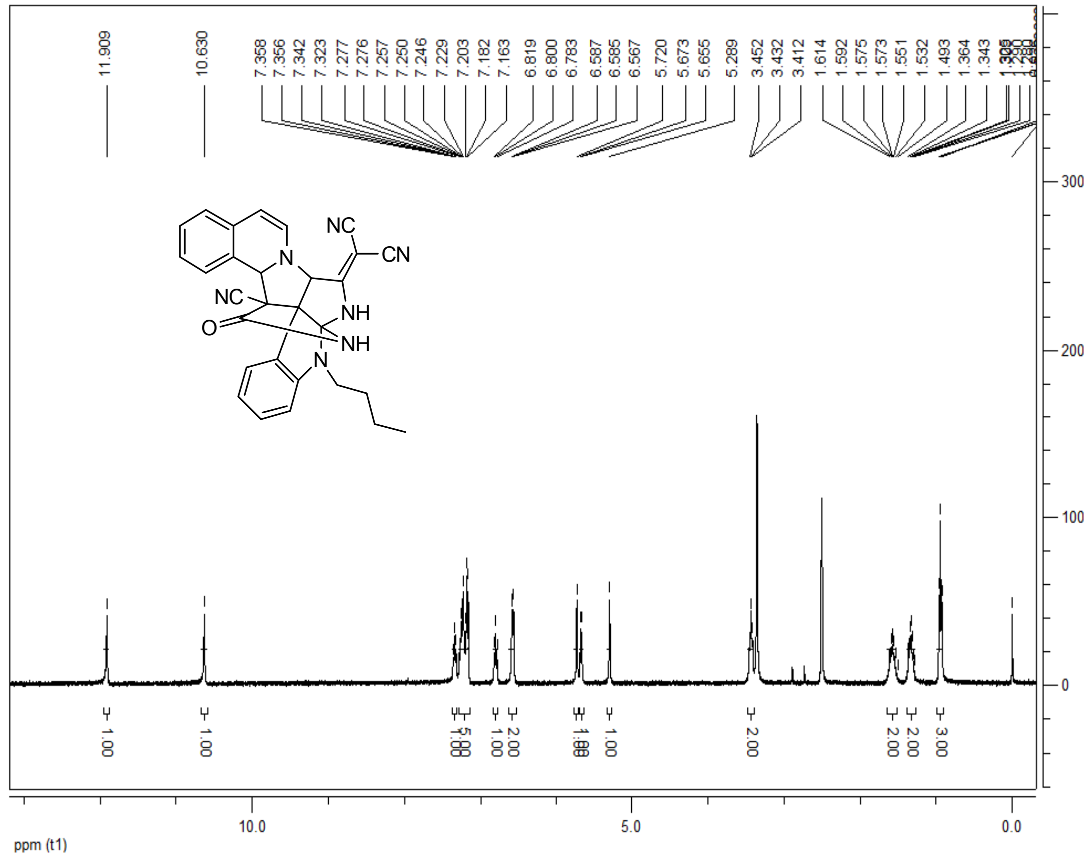

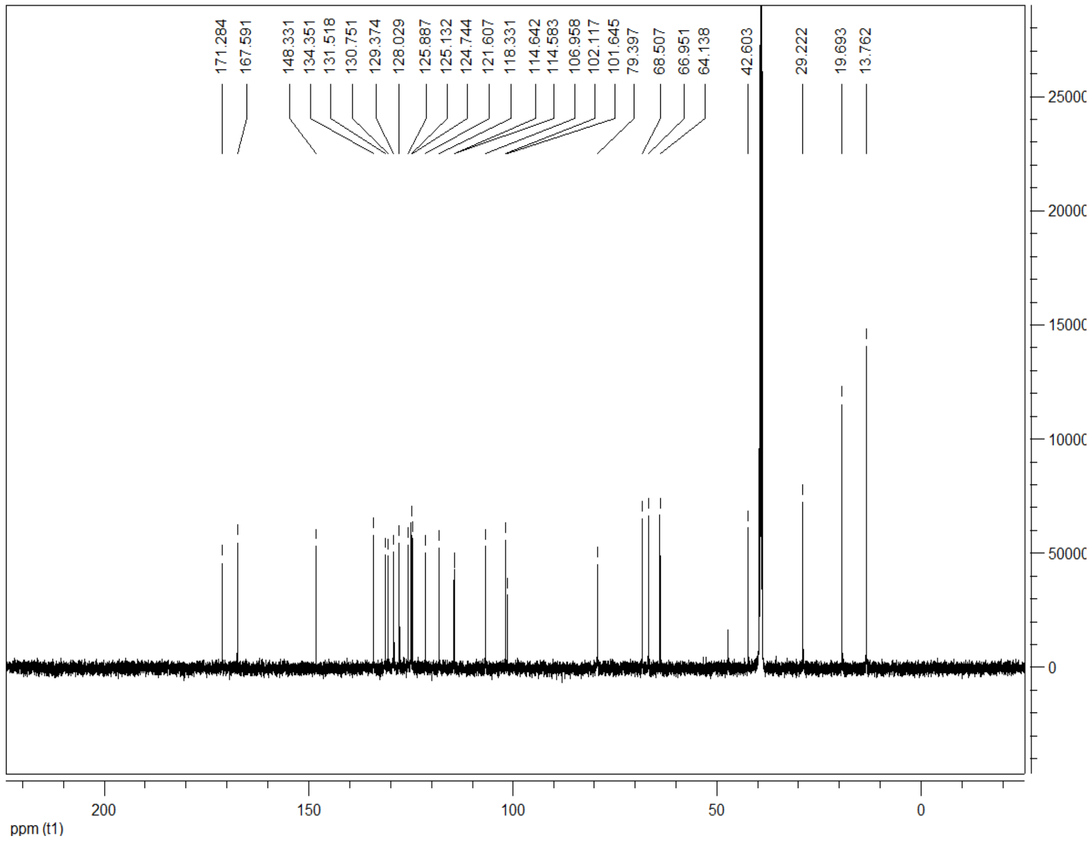

5-butyl-2-chloro-15-cyano-16-oxo-14b,15-dihydro-5a,15-(epiminomethano)indolo[2'',3'':2',3']pyrrolo[3',4':4,5]pyrrolo[2,1-a]isoquinolin-7(5H,6H,7aH)-ylidene)malononitrile (2i):

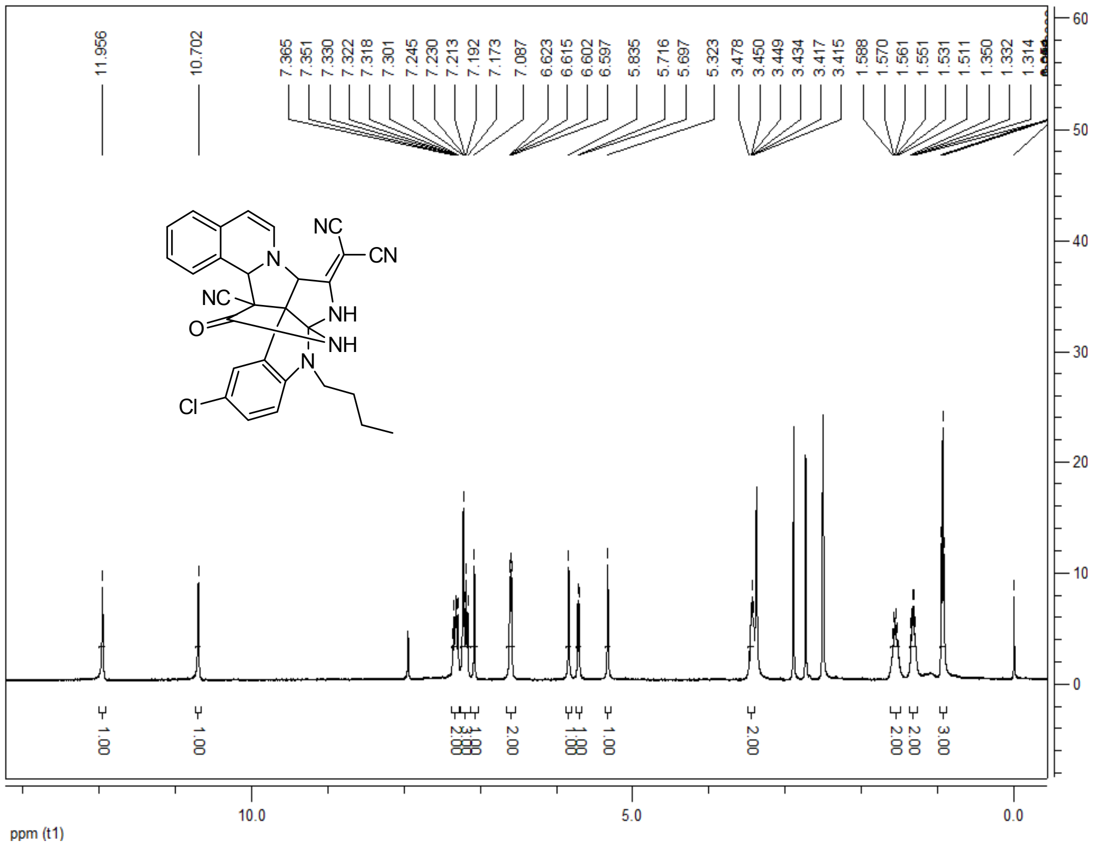

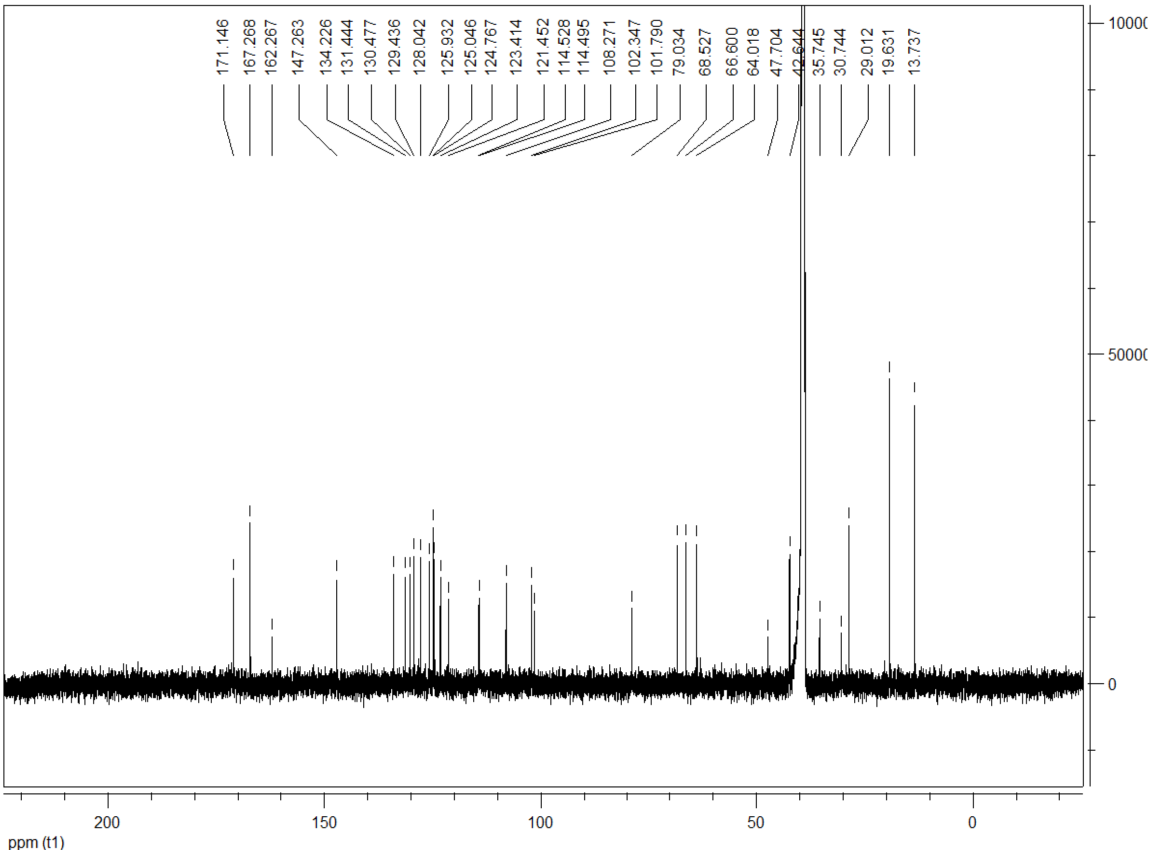

5-butyl-15-cyano-2-methyl-16-oxo-14b,15-dihydro-5a,15-(epiminomethano)indolo[2'',3'':2',3']pyrrolo[3',4':4,5]pyrrolo[2,1-a]isoquinolin-7(5H,6H,7aH)-ylidene)malononitrile (2j):

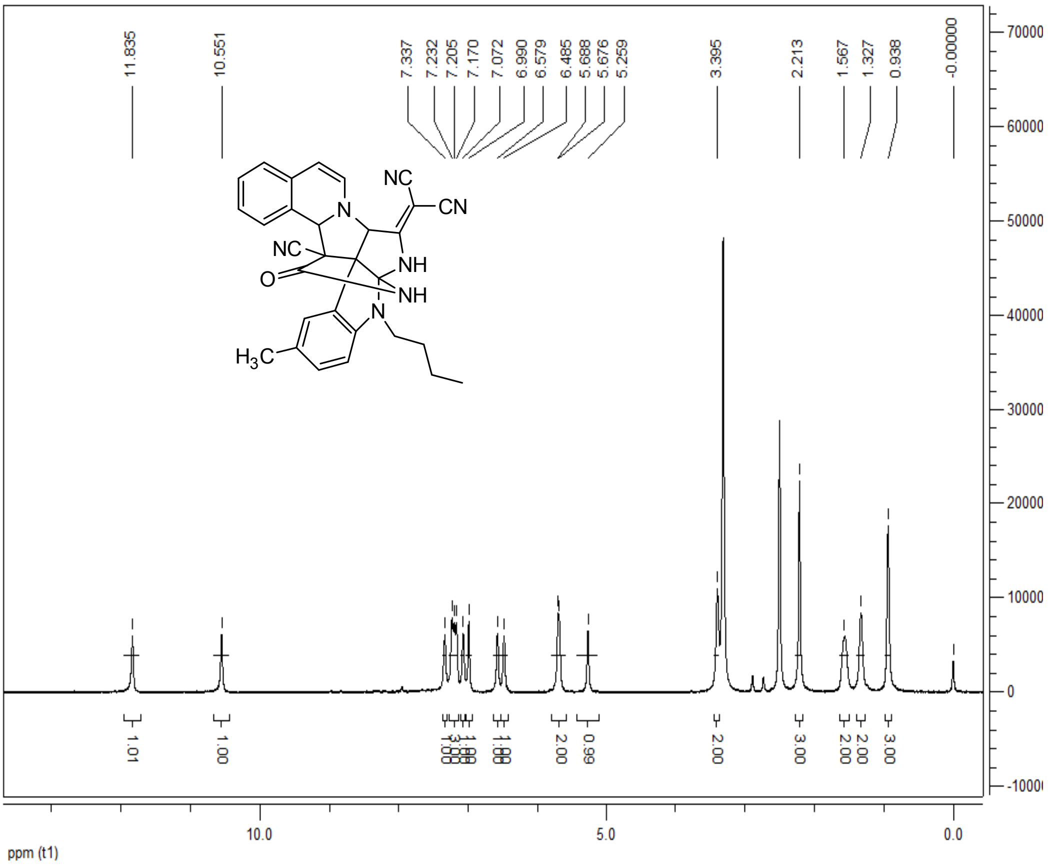

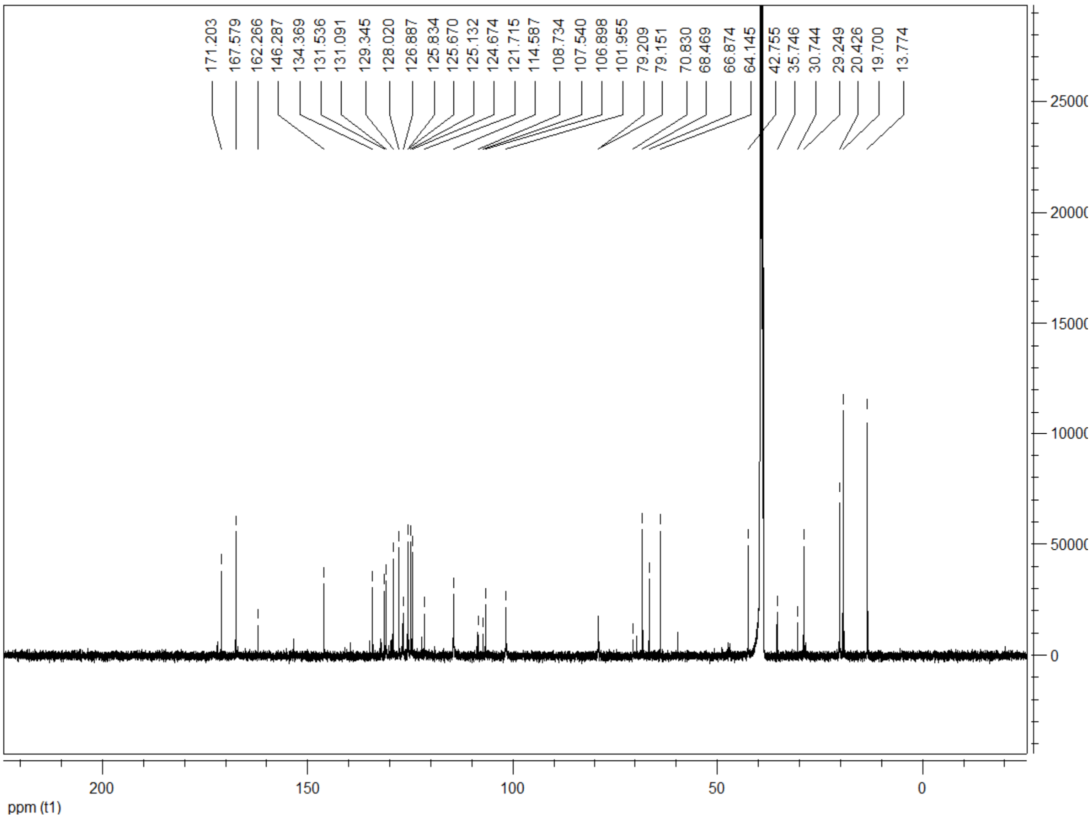

15-cyano-5-methyl-16-oxo-14b,15-dihydro-5a,15-(epiminomethano)indolo[2'',3'':2',3']pyrrolo[3',4':4,5]pyrrolo[2,1-a]isoquinolin-7(5H,6H,7aH)-ylidene)malononitrile (2k):

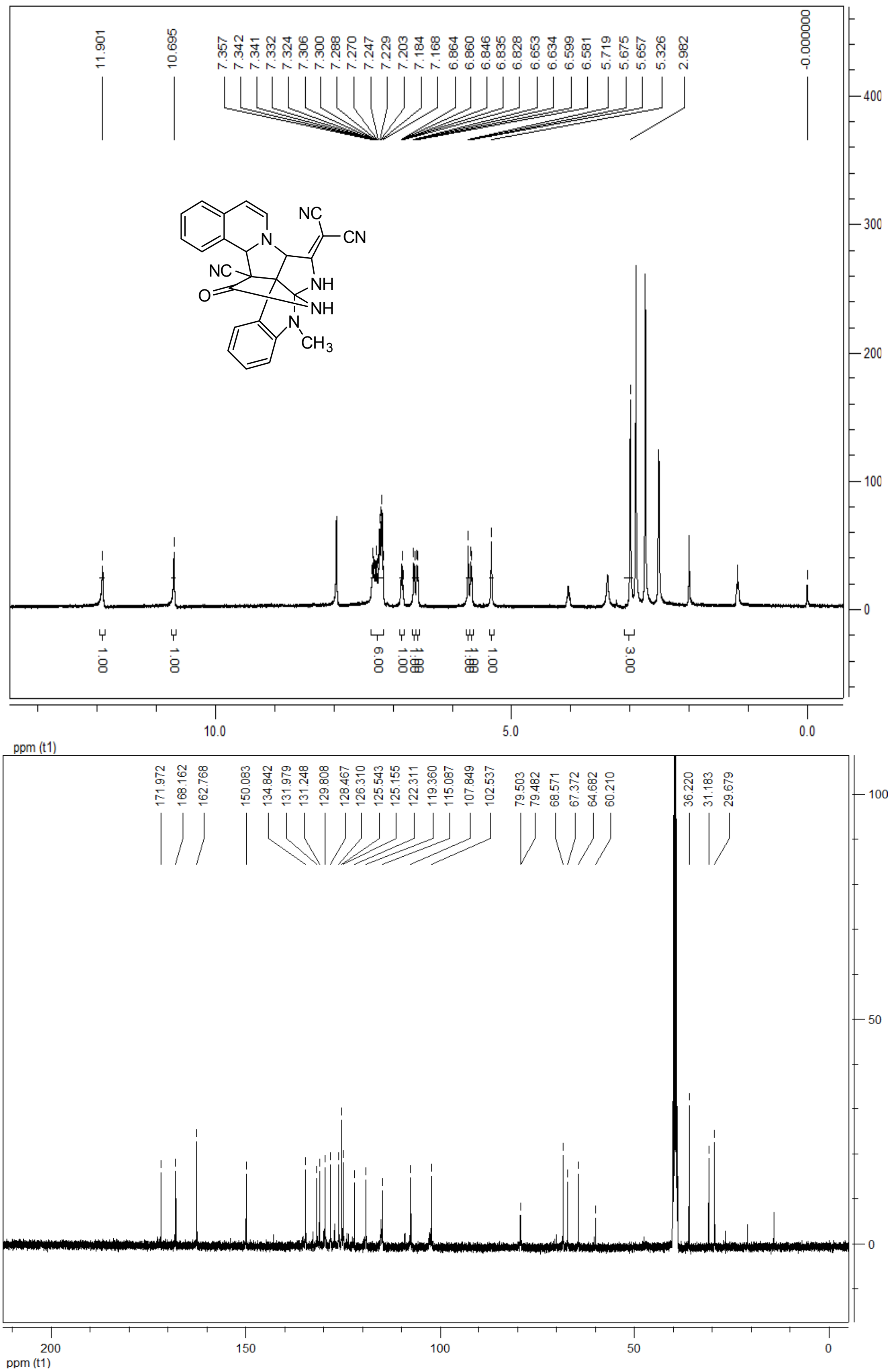

2-chloro-15-cyano-5-(4-methylbenzyl)-16-oxo-14b,15-dihydro-5a,15-(epiminomethano)indolo[2'',3'':2',3']pyrrolo[3',4':4,5]pyrrolo[2,1-a]isoquinolin-7(5H,6H,7aH)-ylidene)malononitrile (2l):

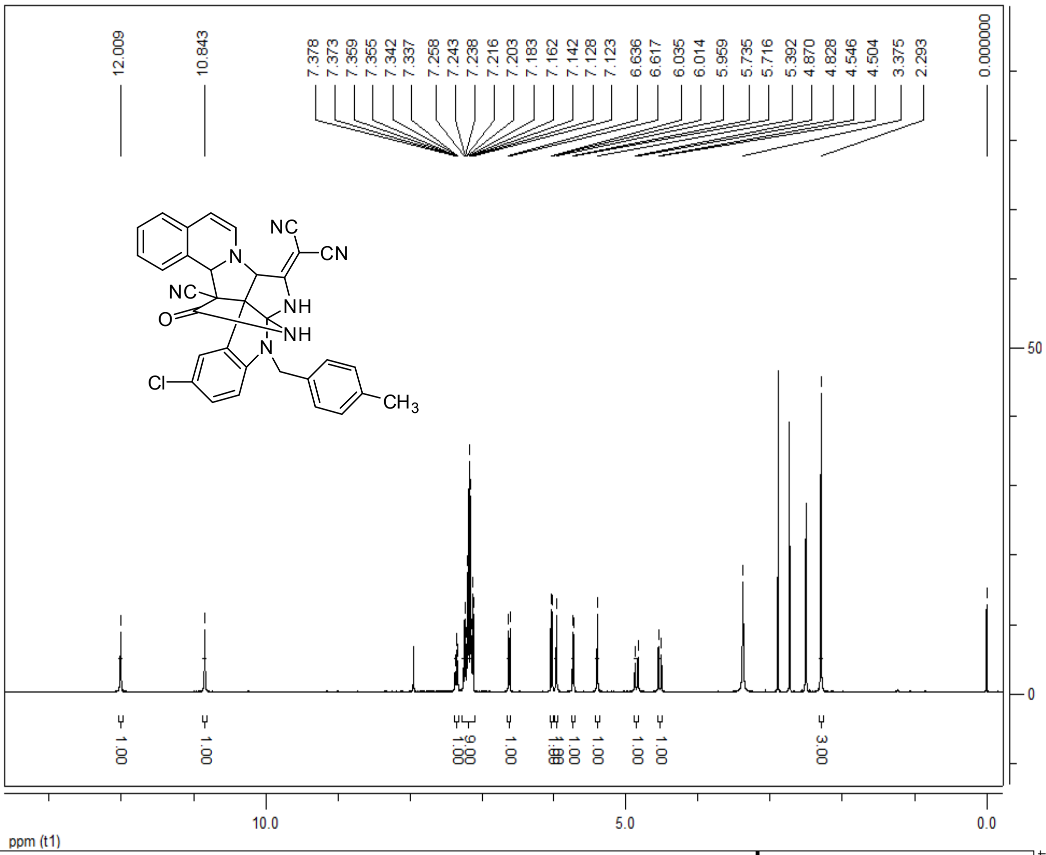

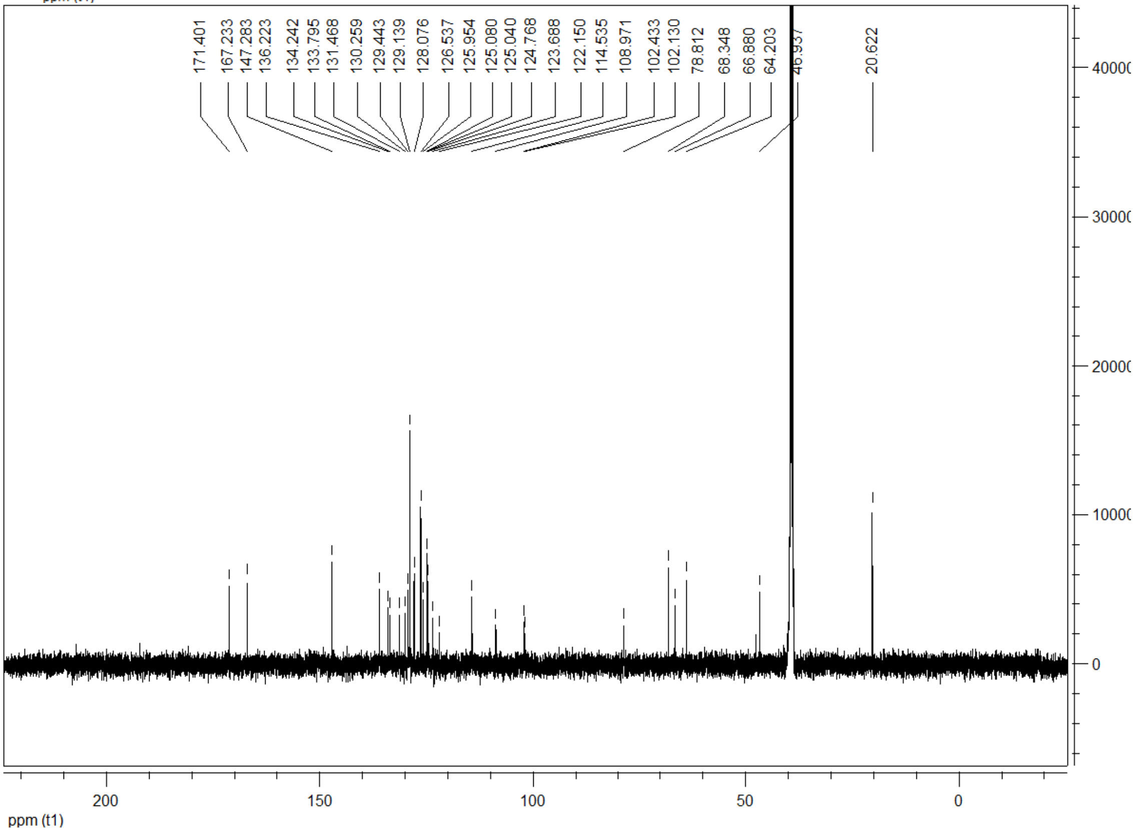

(6'-amino-1-benzyl-5'-cyano-3'-(isoquinolin-2-ium-2-yl)-5-methyl-2-oxo-3'H-spiro[indoline-3,4'-pyridin]-2'-yl)(cyano)(isocyano)methanide (3a):

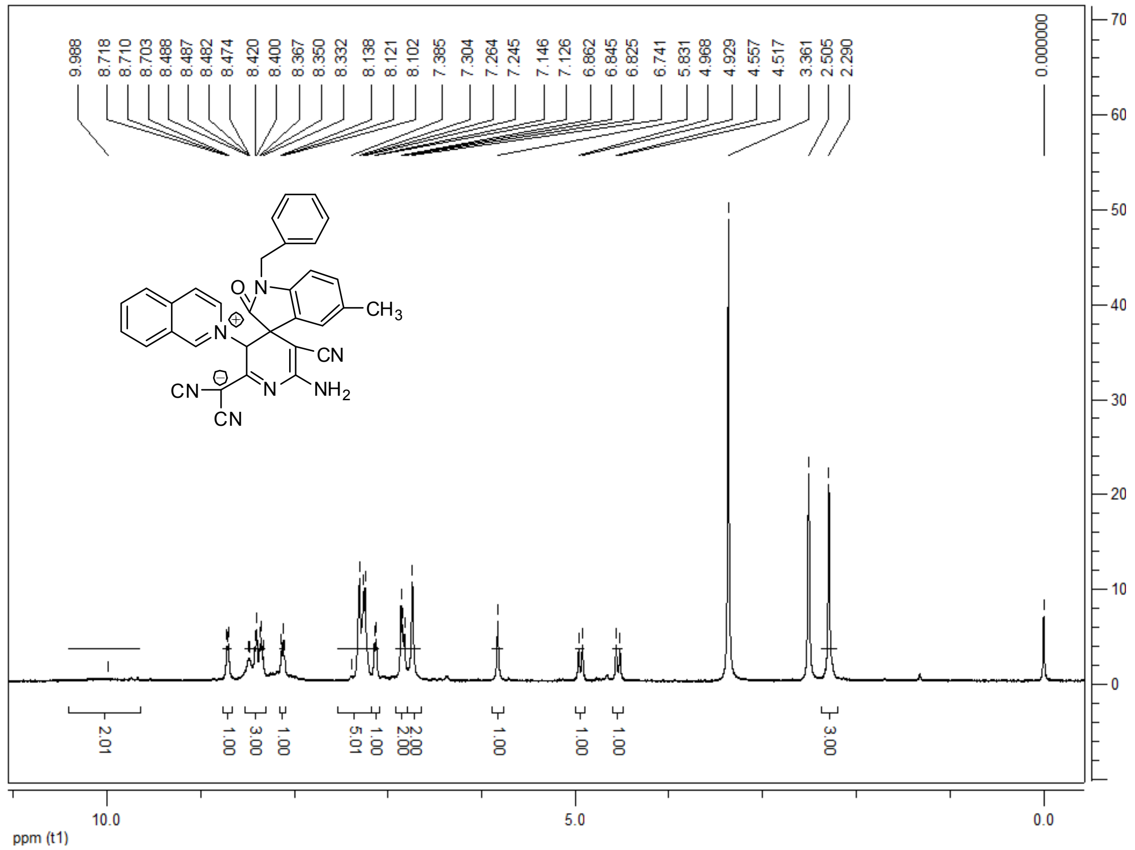

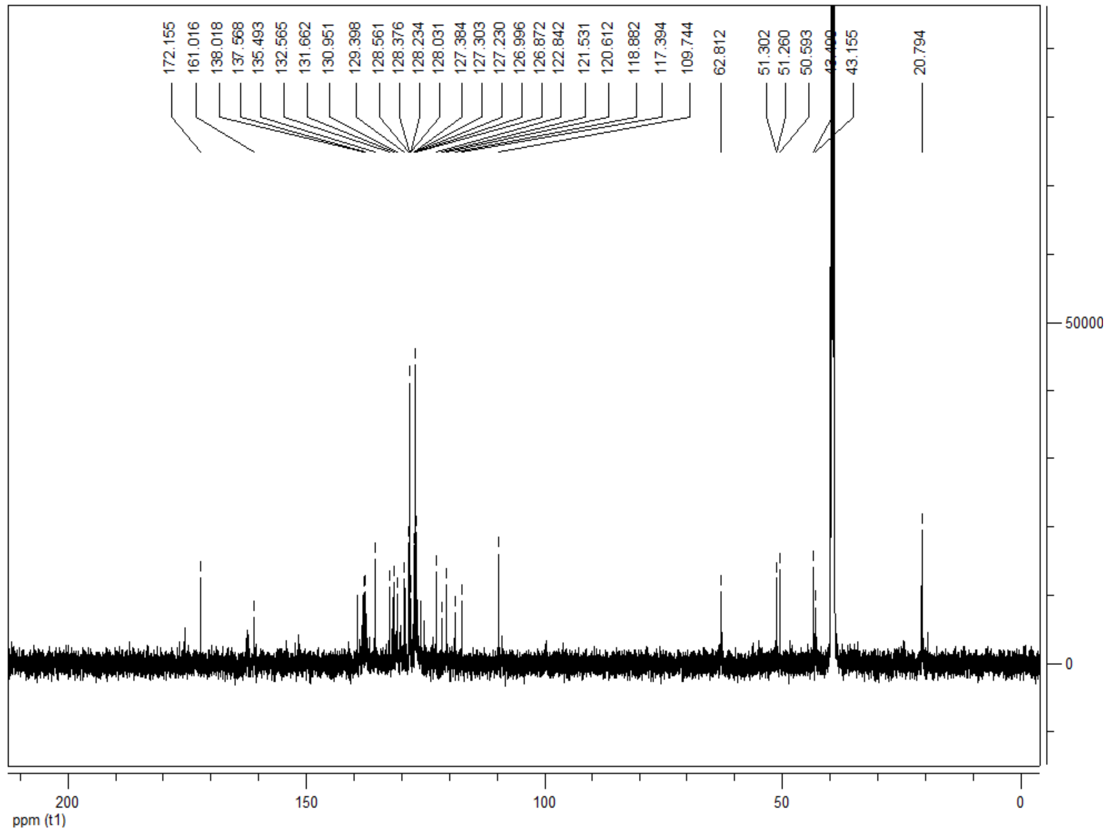

6'-amino-1-butyl-5'-cyano-3'-(isoquinolin-2-ium-2-yl)-5-methyl-2-oxo-3'H-spiro[indoline-3,4'-pyridin]-2'-yl)(cyano)(isocyano)methanide (3b):

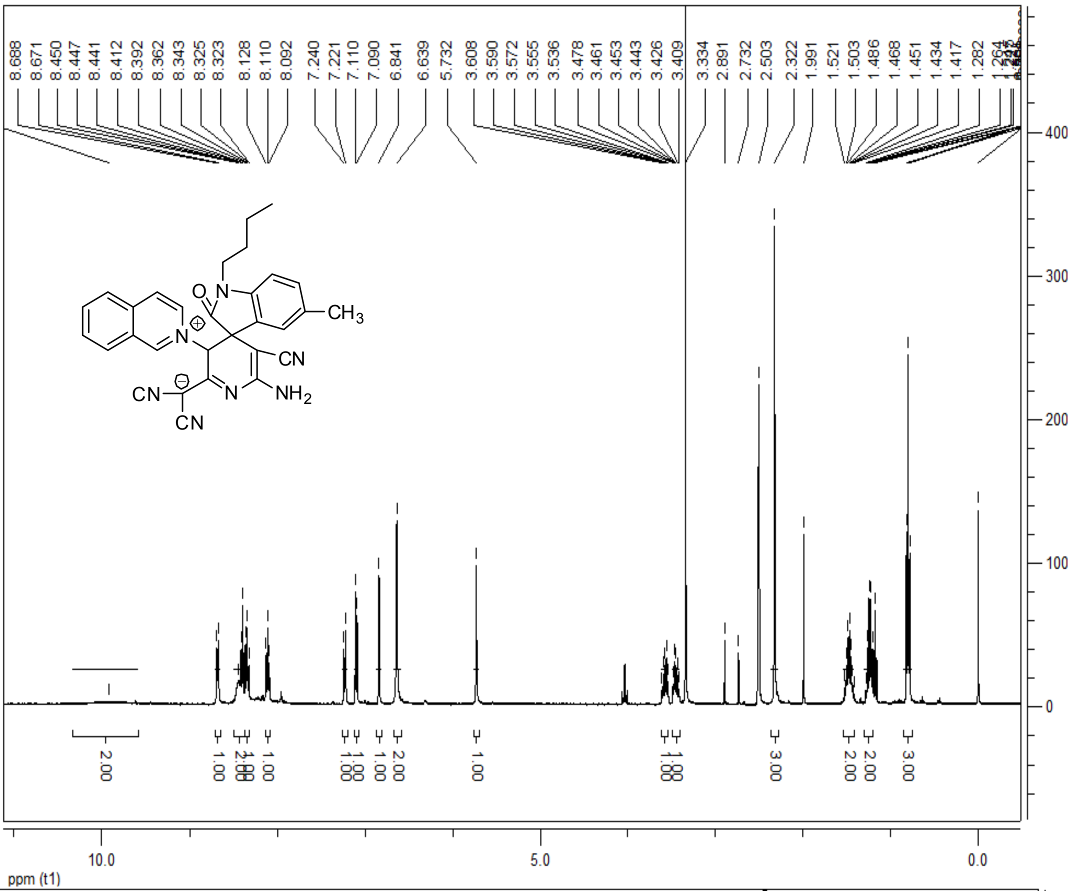

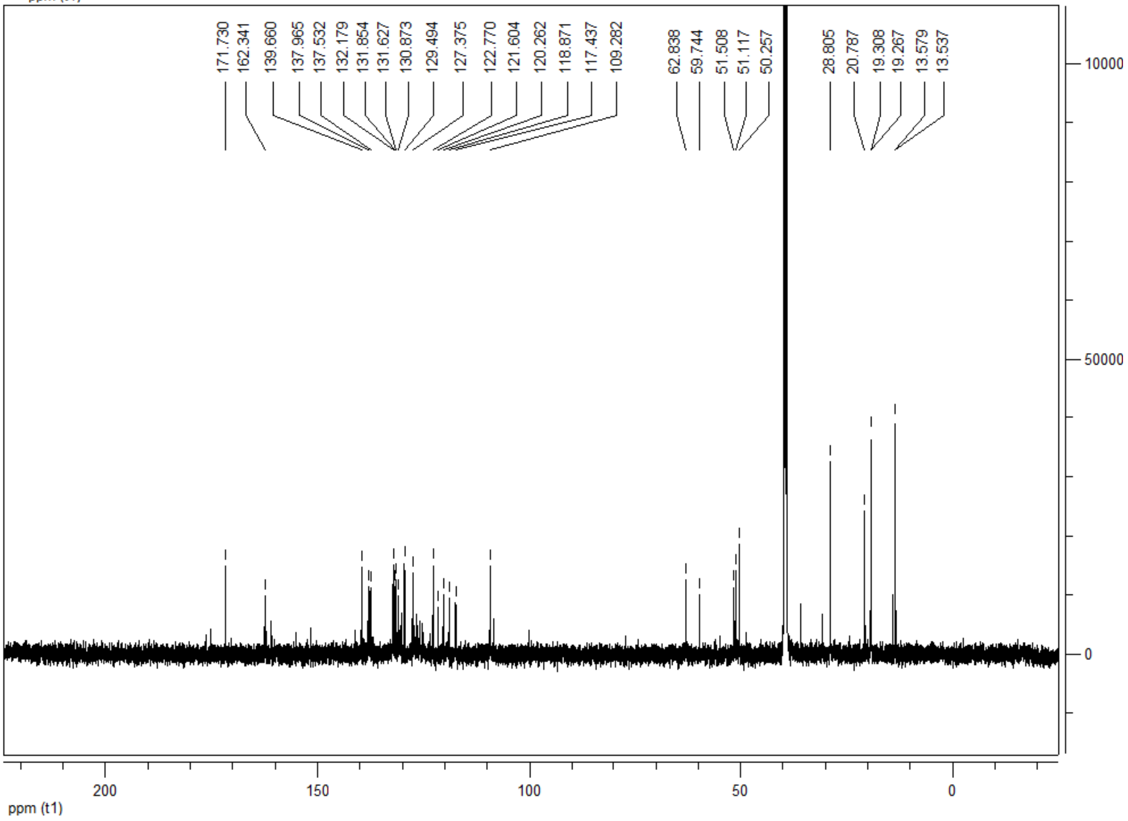

Ethyl 10'-amino-1-benzyl-12'-cyano-2-oxo-11'H-spiro[indoline-3,8'-pyrido[2',3':4,5]pyrrolo-[2,1-a]isoquinoline]-9'-carboxylate (4a):

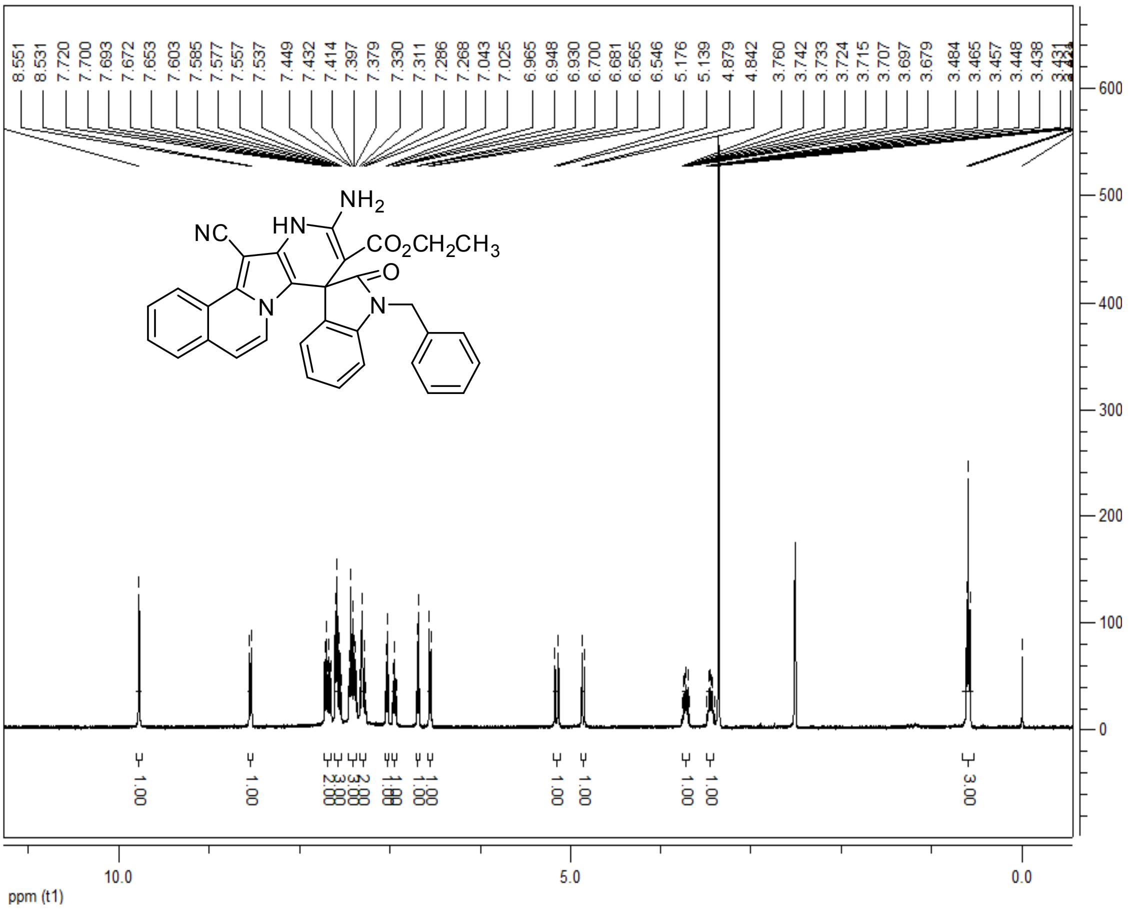

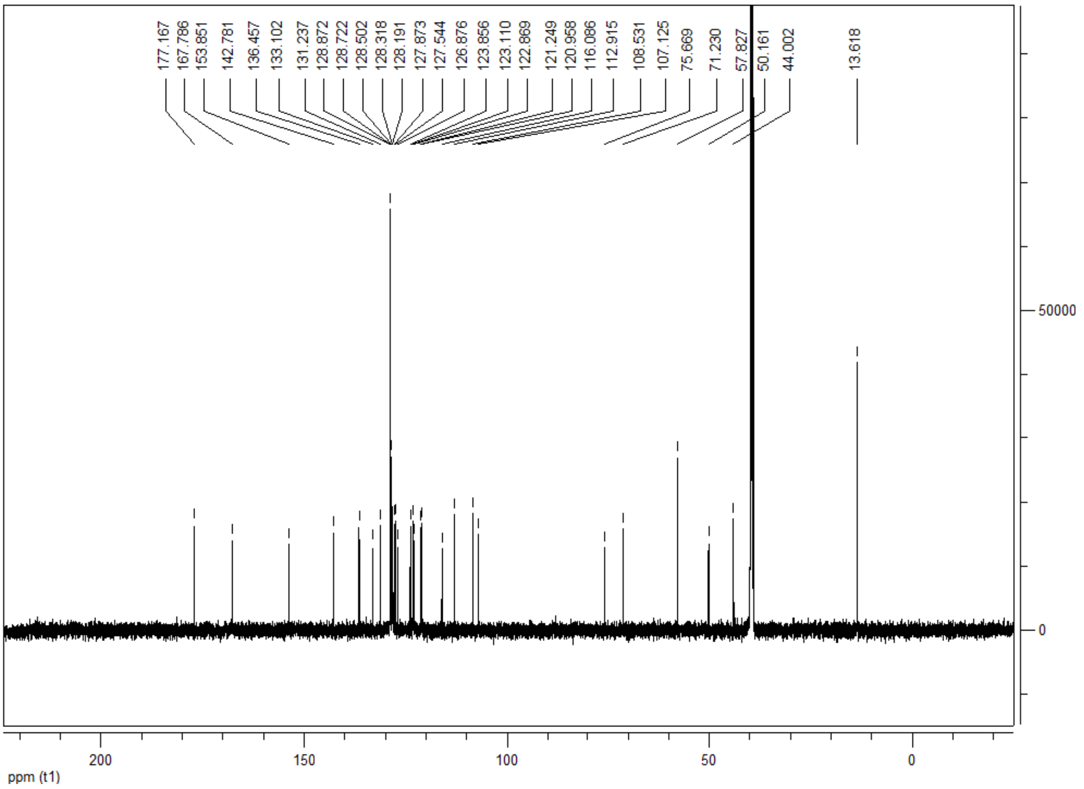

Ethyl 10'-amino-1-benzyl-12'-cyano-5-fluoro-2-oxo-11'H-spiro[indoline-3,8'-pyrido-[2',3':4,5]pyrrolo[2,1-a]isoquinoline]-9'-carboxylate (4b):

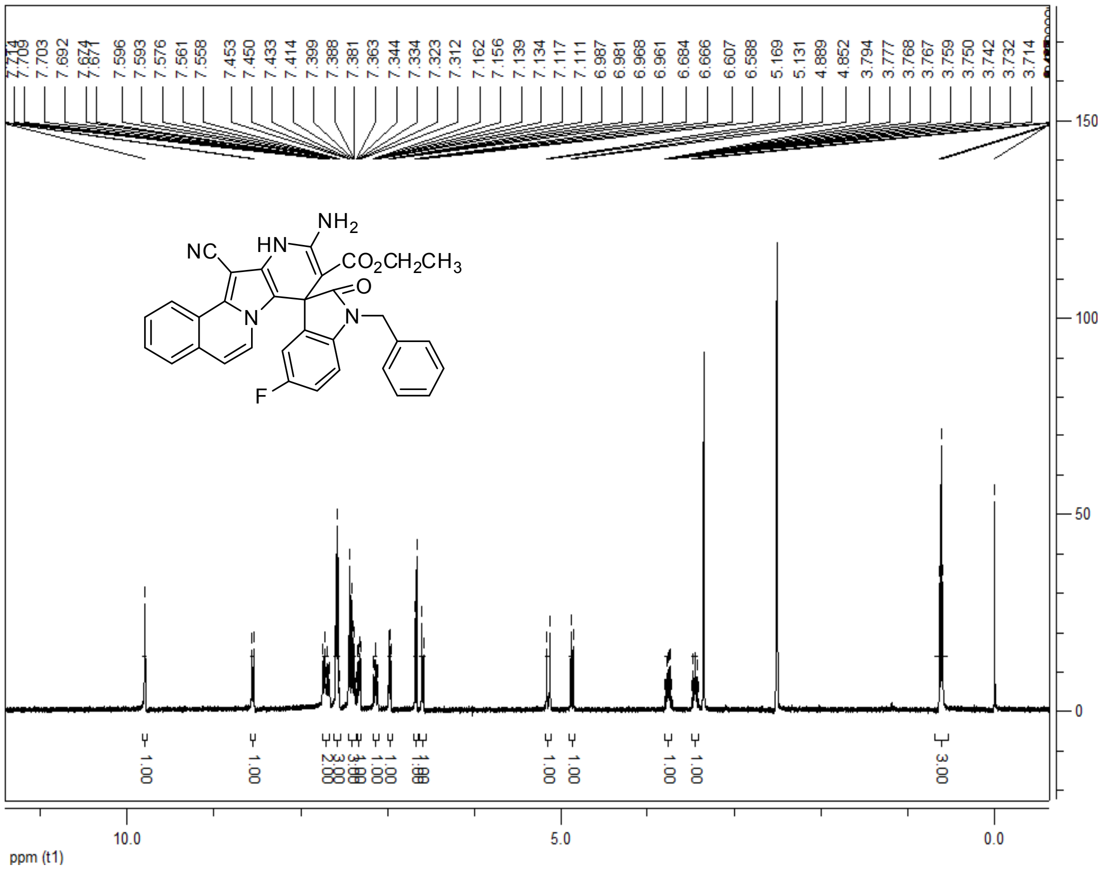

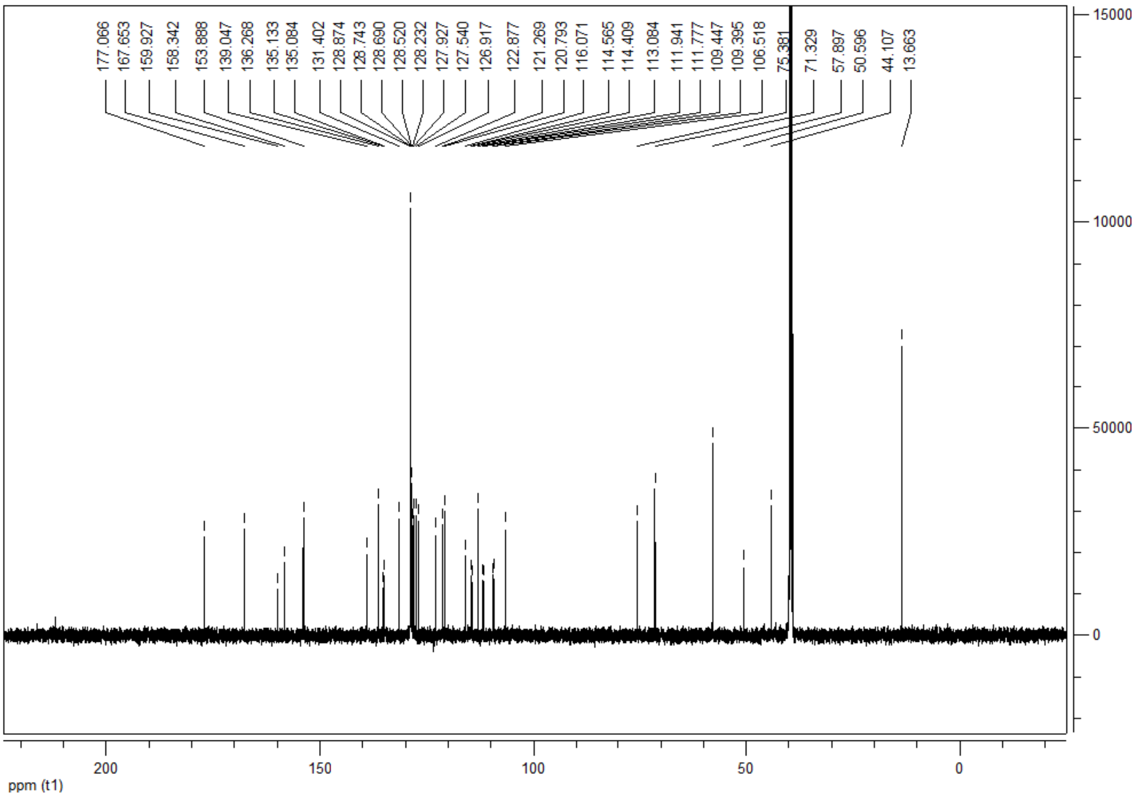

Ethyl 10'-amino-1-benzyl-5-chloro-12'-cyano-2-oxo-11'H-spiro[indoline-3,8'-pyrido-[2',3':4,5]pyrrolo[2,1-a]isoquinoline]-9'-carboxylate (4c):

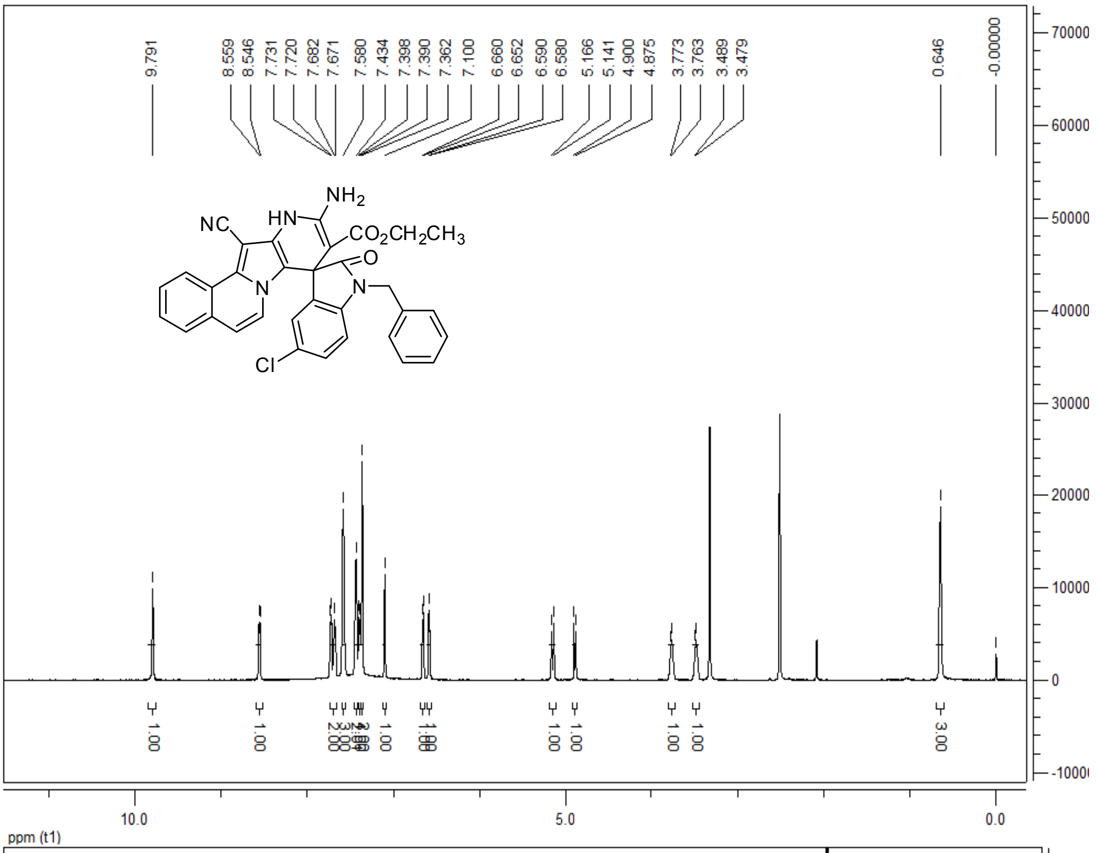

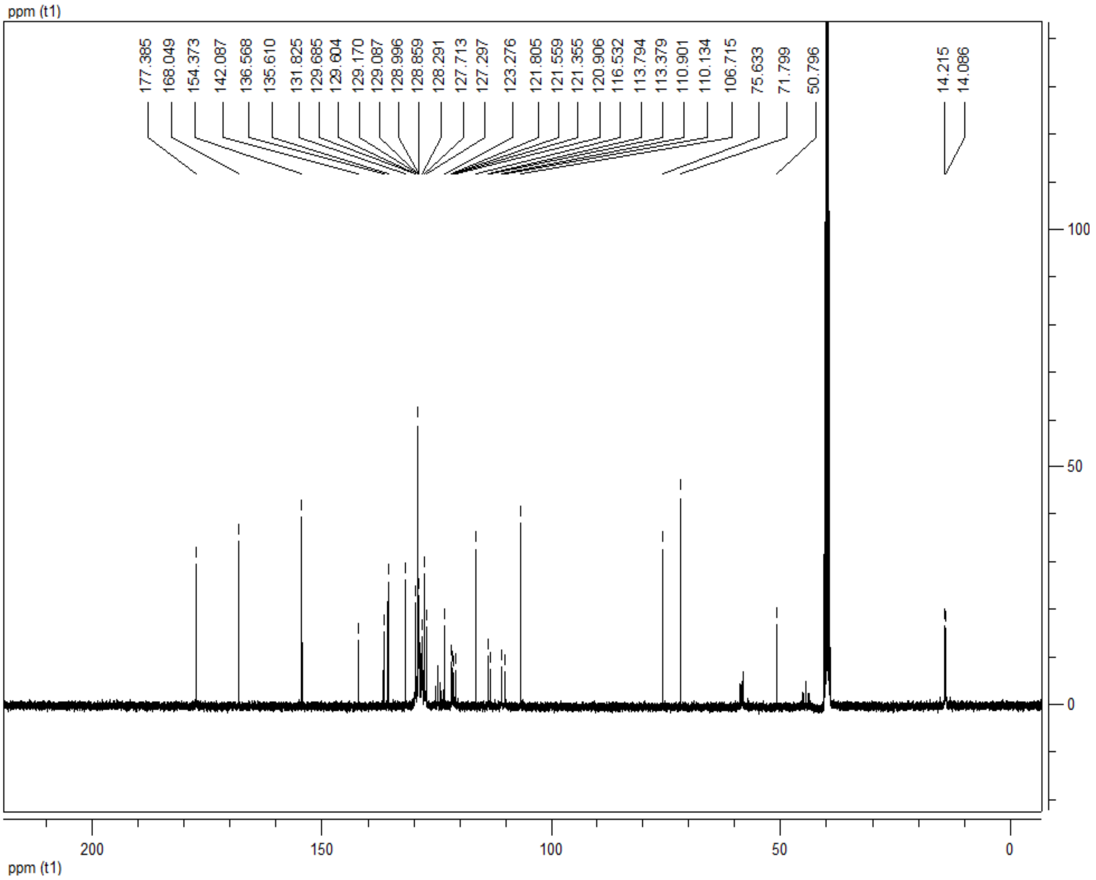

Ethyl 10'-amino-1-butyl-5-chloro-12'-cyano-2-oxo-11'H-spiro[indoline-3,8'-pyrido-[2',3':4,5]pyrrolo[2,1-a]isoquinoline]-9'-carboxylate (4d):

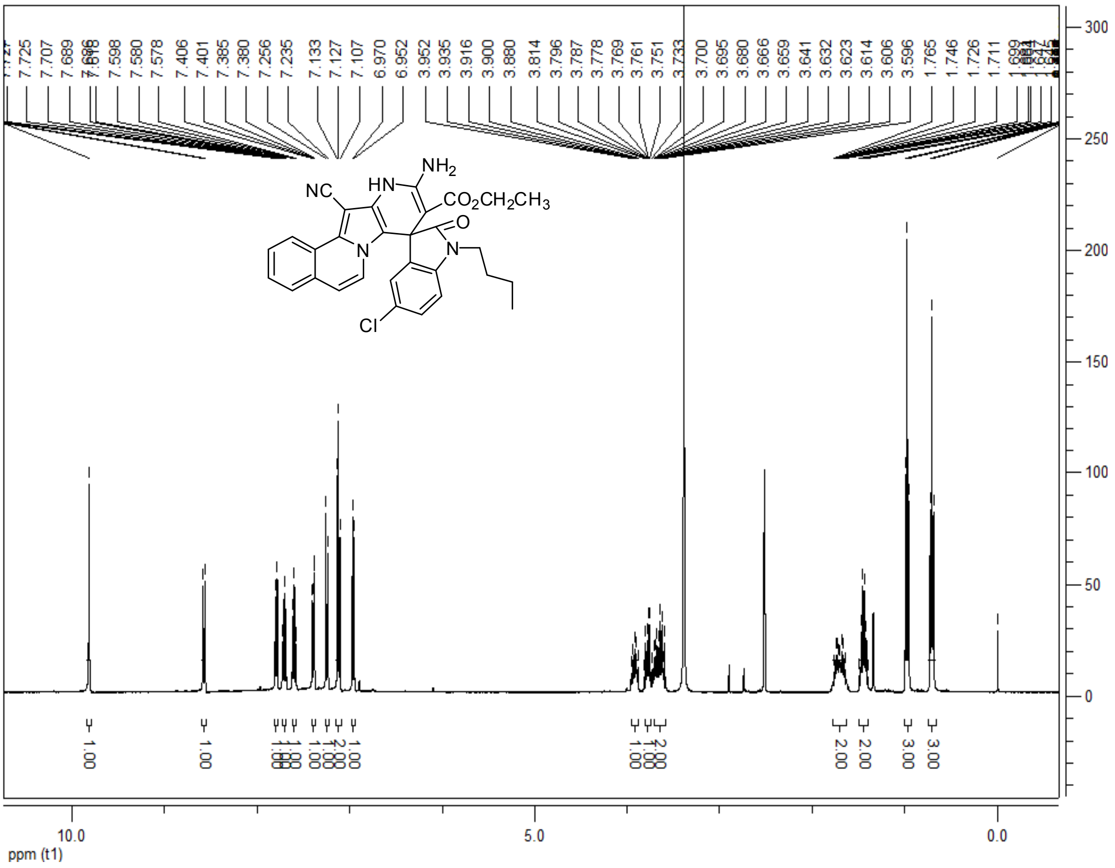

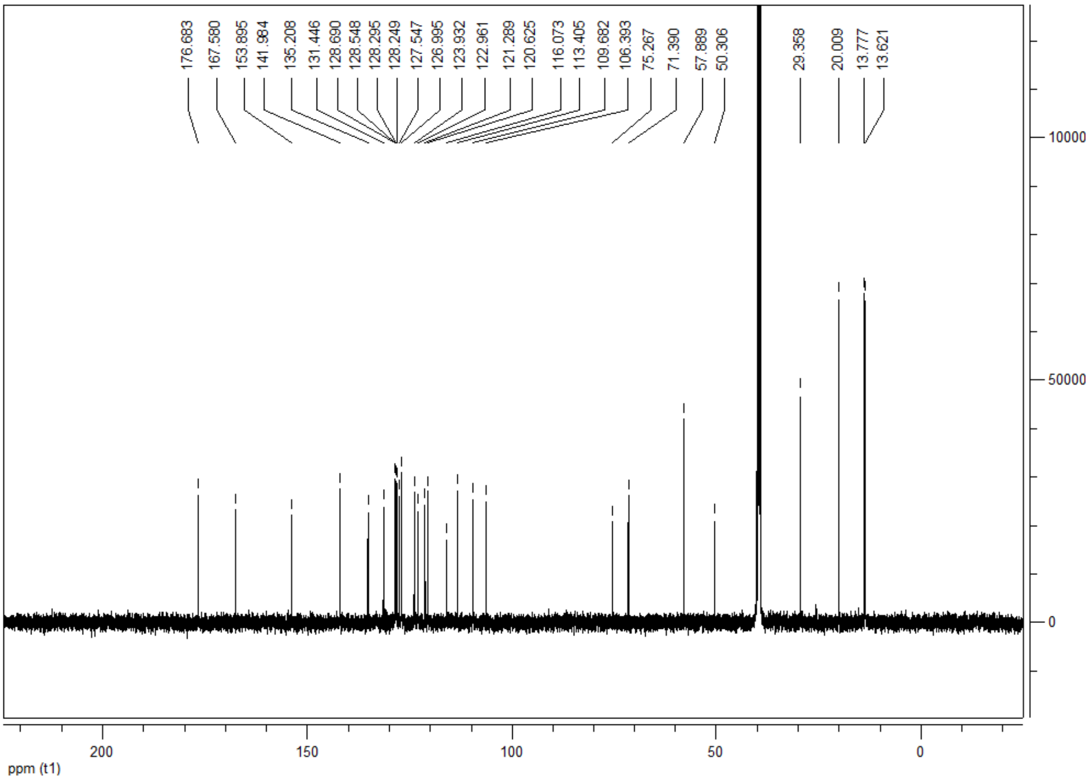

Ethyl 10'-amino-1-butyl-12'-cyano-5-methyl-2-oxo-11'H-spiro[indoline-3,8'-pyrido-[2',3':4,5]pyrrolo[2,1-a]isoquinoline]-9'-carboxylate (4e):

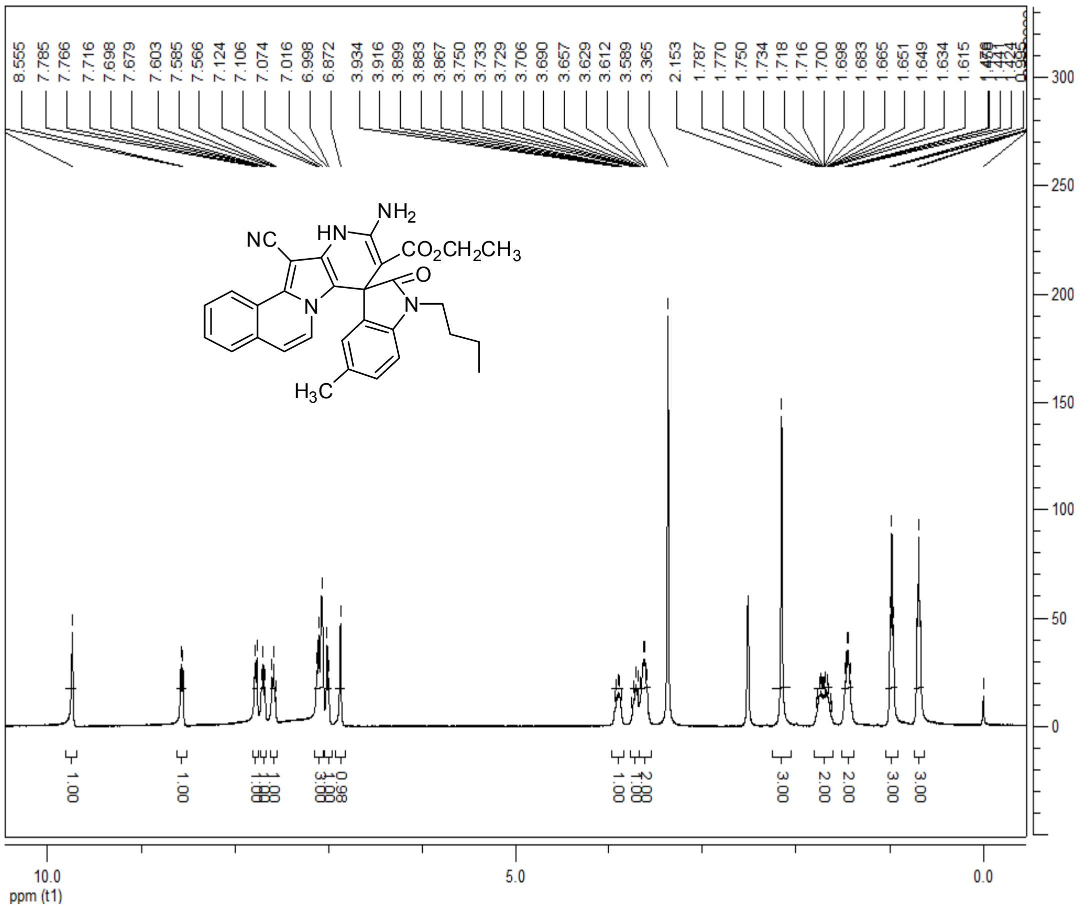

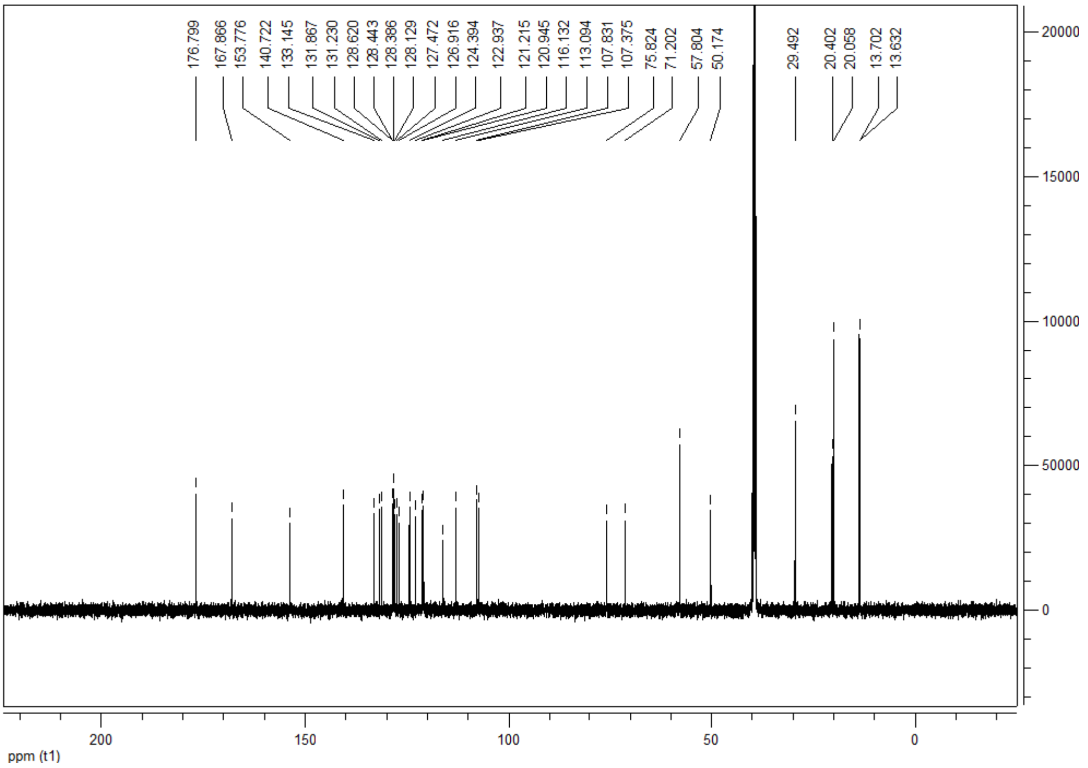

Methyl 10'-amino-1-benzyl-5-chloro-12'-cyano-2-oxo-11'H-spiro[indoline-3,8'-pyrido-[2',3':4,5]pyrrolo[2,1-a]isoquinoline]-9'-carboxylate (4f):

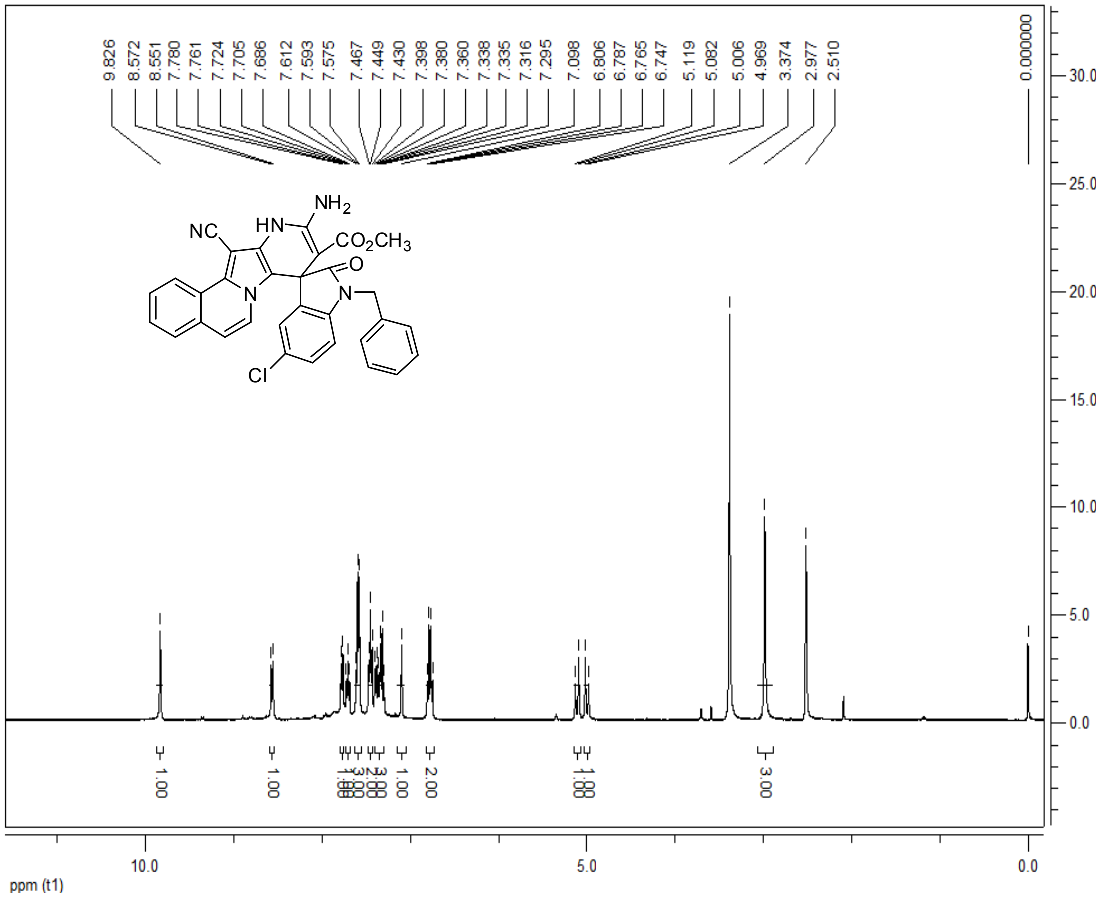

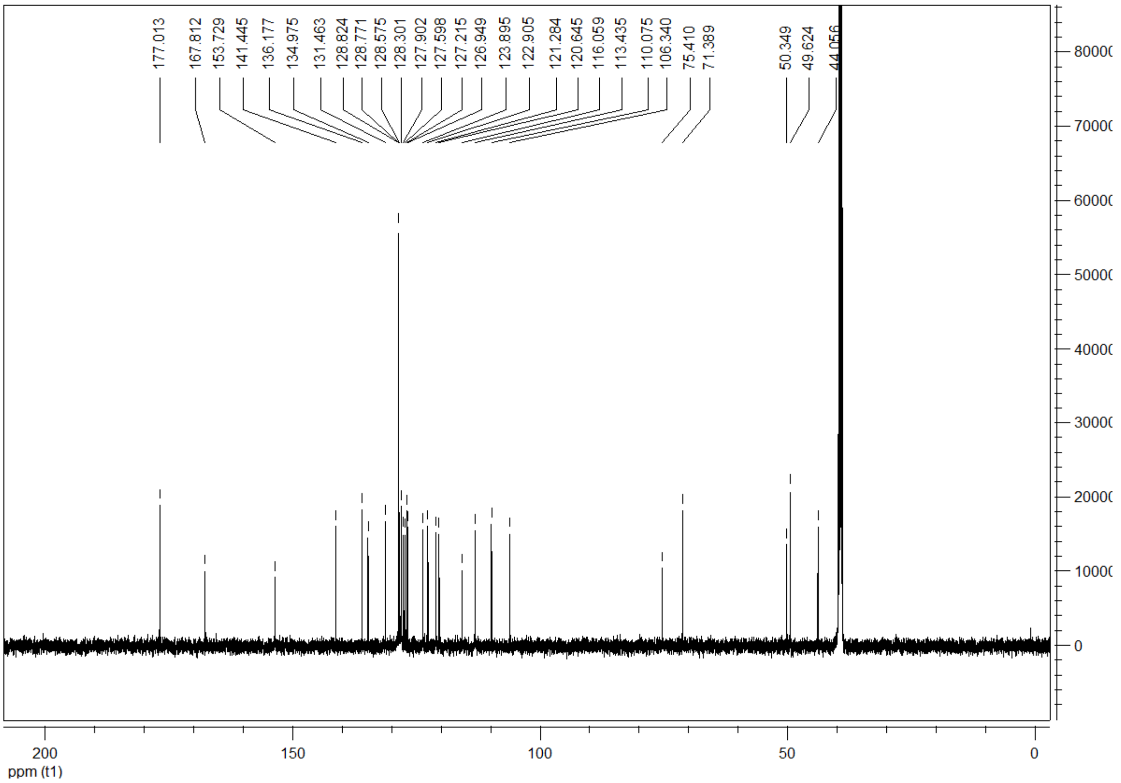

Methyl 10'-amino-1-benzyl-12'-cyano-5-methyl-2-oxo-11'H-spiro[indoline-3,8'-pyrido-[2',3':4,5]pyrrolo[2,1-a]isoquinoline]-9'-carboxylate (4g):

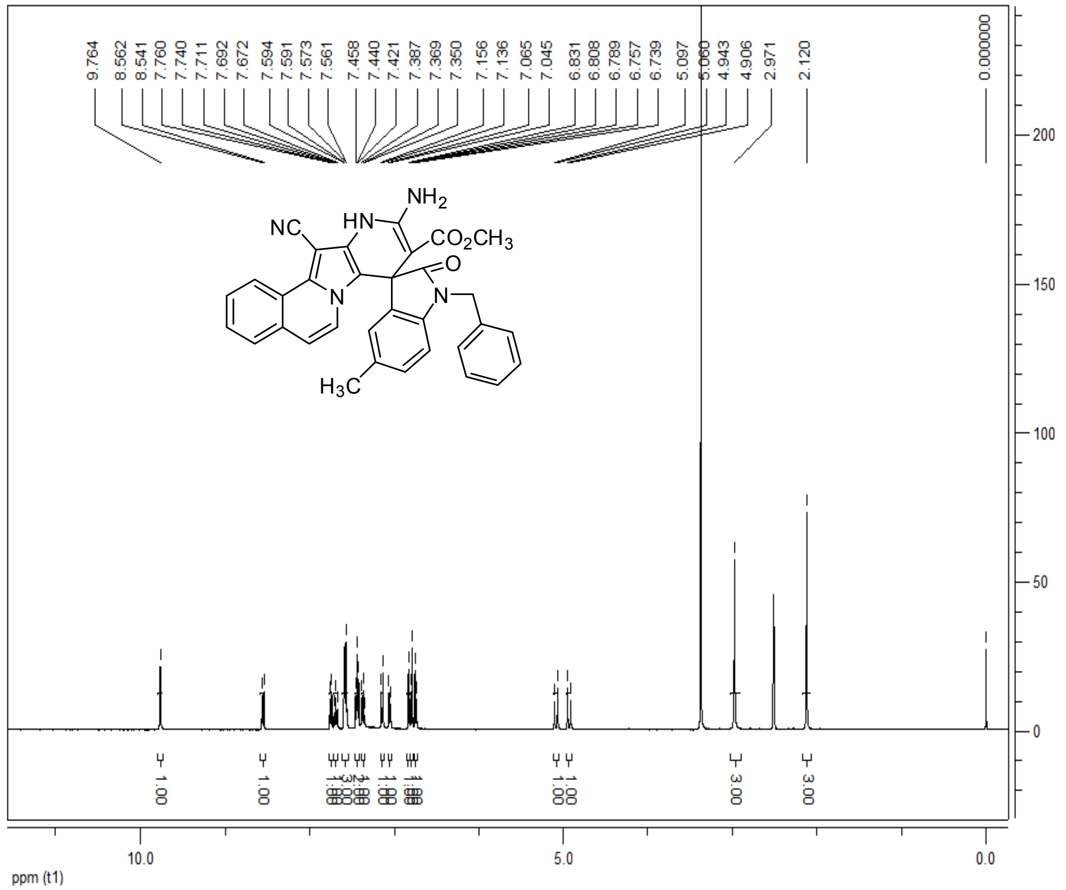

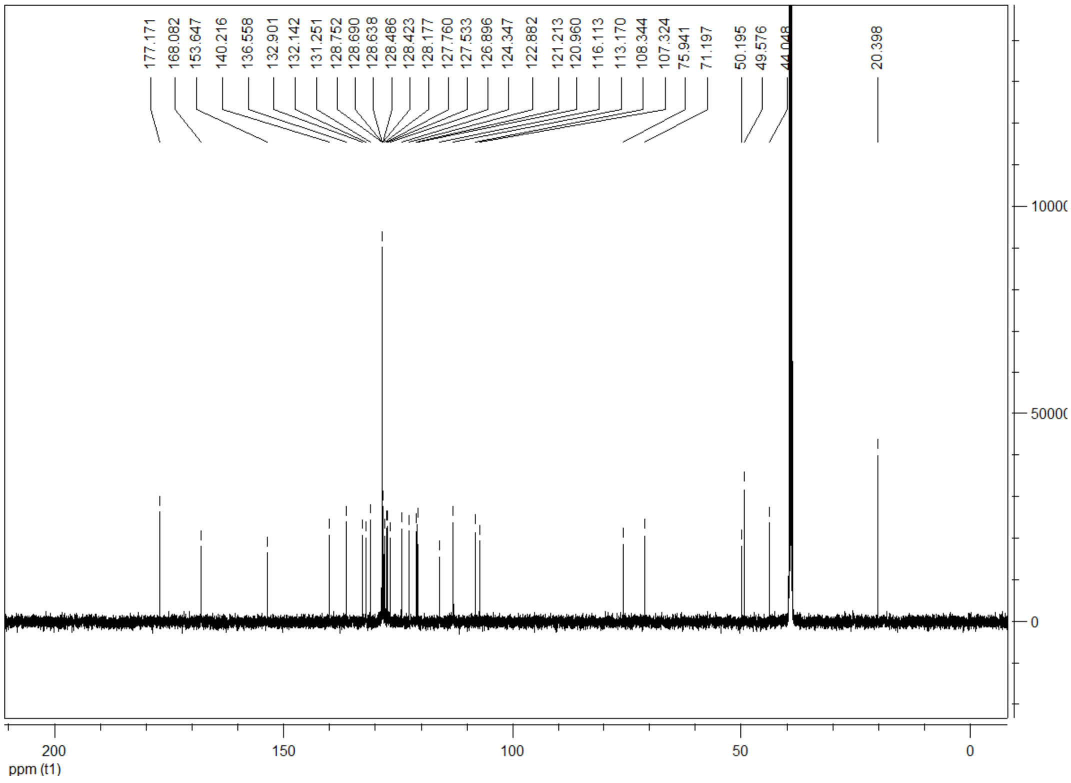

Supplement: Supplementary Information [file srep41024-s1.pdf]
